# Integration of aerobic oxidation and intramolecular asymmetric aza-Friedel–Crafts reactions with a chiral bifunctional heterogeneous catalyst†
†Electronic supplementary information (ESI) available: General procedures, materials, and instrumentation; synthesis, characterization and relevant spectra/charts; procedures and results for optimization and additional experiments. See DOI: 10.1039/c6sc03849b



**DOI:** 10.1039/c6sc03849b

**Published:** 2016-10-05

**Authors:** Hong-Gang Cheng, Javier Miguélez, Hiroyuki Miyamura, Woo-Jin Yoo, Shū Kobayashi

**Affiliations:** a Department of Chemistry , School of Science , The University of Tokyo , Hongo, Bunkyo-ku , Tokyo 113-0033 , Japan . Email: shu_kobayashi@chem.s.u-tokyo.ac.jp

## Abstract

A new class of chiral bifunctional heterogeneous materials composed of Au/Pd nanoparticles and chiral phosphoric acids as active orthogonal catalysts was prepared by utilizing a facile pseudo-suspension co-polymerization method. It was found that this heterogeneous catalyst was capable of facilitating the sequential aerobic oxidation-asymmetric intramolecular aza-Friedel–Crafts reaction between benzyl alcohols and *N*-aminoethylpyrroles. Moreover, the designed chiral heterogeneous catalyst could be recovered and reused several times without significant loss of activity or enantioselectivity.

## Abstract 2

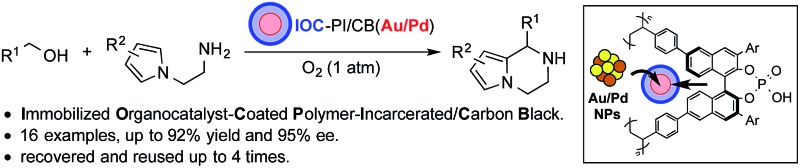
A chiral heterogeneous material was prepared and evaluated as a bifunctional catalyst for the sequential aerobic oxidation-asymmetric intramolecular aza-Friedel–Crafts reaction.

## Introduction

Multicatalyst-promoted asymmetric tandem reactions^1^ are an emerging subset in the family of one-pot processes^2^ that can provide access to complex organic substrates of high enantiopurity in an efficient and practical manner. Despite the promise of these reactions, one of the major challenges that has limited their development is the problem associated with catalyst incompatibility. A potential strategy to overcome this obstacle is by applying the principle of site separation to prevent mutual deactivation of catalysts, and successful tandem asymmetric processes have been achieved with chiral catalysts immobilized or encapsulated on polymers^3^ and sol–gel materials.^4^ Another challenge facing multicatalyst-promoted tandem reactions is the unselective interaction of starting materials and reaction intermediates with the catalysts to generate unwanted by-products. While it is difficult to achieve catalyst selectivity in one-pot reaction process, the use of heterogeneous catalysts in continuous-flow systems is a potential solution to overcome this problem.^5^

**Scheme 1:**
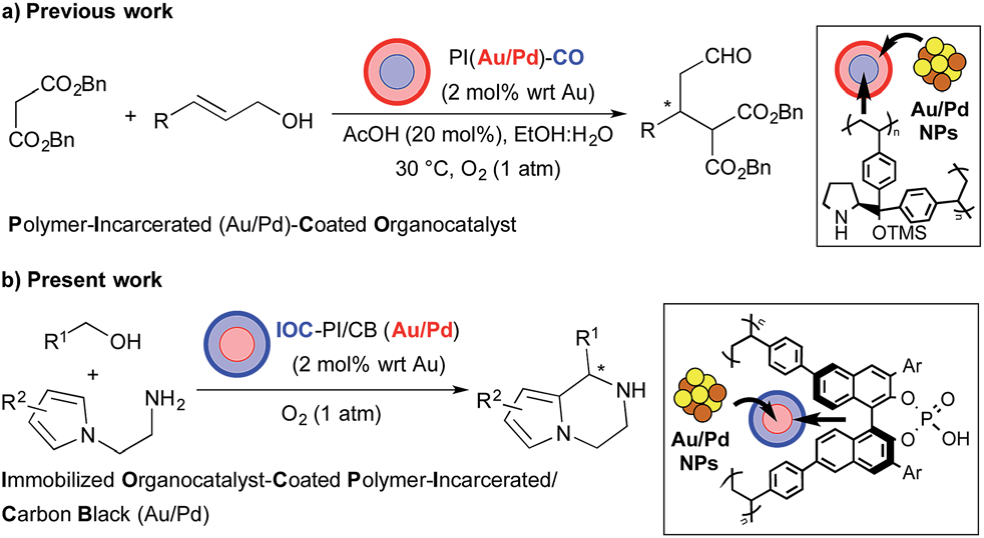
Chiral bifunctional heterogeneous catalysts for TOPs.

Our group has a long-standing interest in the immobilization of metal nanoparticles (NPs) onto polymer supports^6^ and its application to tandem oxidation processes (TOPs) with oxygen gas as the terminal oxidant.^7^ Previously, we reported the fabrication of a layered heterogeneous bifunctional chiral catalyst consisting of Au/Pd NPs and a Jørgensen–Hayashi-type organocatalyst supported on separate polymeric materials (PI(Au/Pd)–CO, polymer-incarcerated Au/Pd NP-coated organocatalyst), and its application as a catalyst for the sequential aerobic oxidation-asymmetric Michael reaction of primary allylic alcohols and dibenzyl malonate (Scheme 1a).^3*c*^ While we were able to demonstrate that our fabrication method prevented catalyst deactivation between the Au/Pd NPs and the chiral organocatalyst to enable the asymmetric TOP, it was discovered that the chiral secondary amine catalyst was deactivated under aerobic conditions and that the heterogeneous catalyst could not be reused. Based on our preliminary experimental studies and related literature,^8^ we concluded that the use of chiral heterogeneous secondary amines as organocatalysts for asymmetric TOPs was not a viable strategy due to the propensity of the covalent intermediates to undergo aerobic oxidation and become degraded. Therefore, we hypothesized that the use of chiral organocatalysts which activate the organic substrates through non-covalent interactions would lead to a more robust heterogeneous system that could be recovered and reused even under aerobic conditions.

Over the past decade, chiral phosphoric acids (CPAs) have been shown to be highly efficient catalysts for a wide range of asymmetric transformations.^9,10^ Of particular note is their ability to activate imine derivatives, *via* hydrogen bonding or ion pair interactions (non-covalent interactions), and to promote high levels of stereoinduction of the prochiral electrophiles. Based on these considerations, we rationalized that CPAs might represent the ideal chiral component for a recyclable heterogeneous catalyst that could facilitate asymmetric TOPs. Herein, we report the preparation of a heterogeneous chiral bifunctional catalyst, consisting of Au/Pd NPs and a CPA, and its application to the sequential aerobic oxidation-asymmetric aza-Friedel–Crafts (FC) reaction (Scheme 1b).

## Results and discussion

**Scheme 2:**
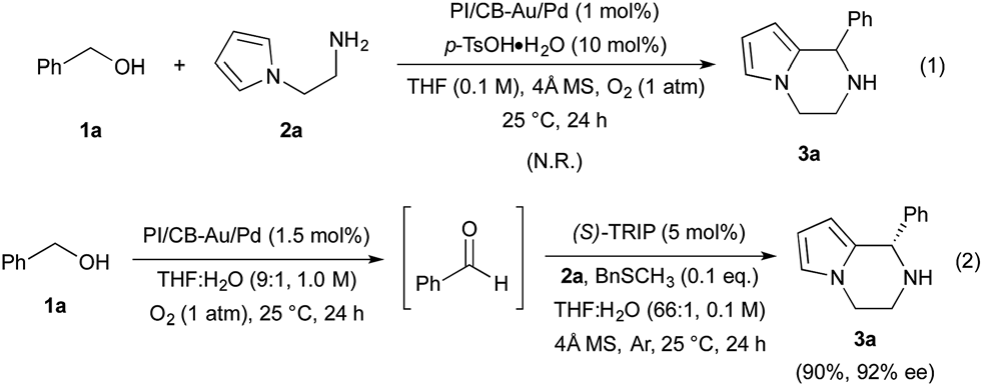
Reaction integration of aerobic oxidation with the aza-FC reaction.

We began our investigations by examining the feasibility of a TOP that integrated aerobic oxidation with the aza-FC reaction, using benzyl alcohol (**1a**) and *N*-aminoethylpyrrole (**2a**) as model substrates and PI/CB–Au/Pd and *p*-toluenesulfonic acid as co-catalysts (Scheme 2, eqn (1)). It was found that the expected piperazine **3a** was not obtained under our initial conditions. This was unexpected given that control studies showed that both the aerobic oxidation of **1a** and the aza-FC reaction between **2a** and benzaldehyde proceeded with good yields under these initial conditions.^11^ It was later revealed that the aerobic oxidation of **1a** did not occur in the presence of **2a**, most likely due to the strong coordination of the primary amine moiety of **2a** to the Au/Pd NPs, causing catalyst deactivation. To overcome this limitation, we found that performing the TOP through a one-pot, sequential addition process, in which the aerobic oxidation of **1a** was allowed to proceed prior to the addition of **2a**, was key to successfully obtaining **3a**. Optimization of the aerobic oxidation step and the asymmetric TOP using (*S*)-3,3′-bis(2,4,6-triisopropylphenyl)-1,1′-binaphthyl-2,2′-diyl hydrogenphosphate ((*S*)-TRIP)^12^ as the CPA was performed in order to improve the yield and enantioselectivity of the desired chiral piperazine **3a**. It was found that water was essential to promote the aerobic oxidation of **1a**, while the introduction of BnSCH_3_ as an additive to deactivate the Au/Pd NPs was important to prevent oxidation of **3a** to its imine form. Based on these two major modifications, the asymmetric TOP proceeded well to deliver chiral piperazine **3a** in high yield and enantioselectivity (Scheme 2, eqn (2)).

**Fig. 1:**
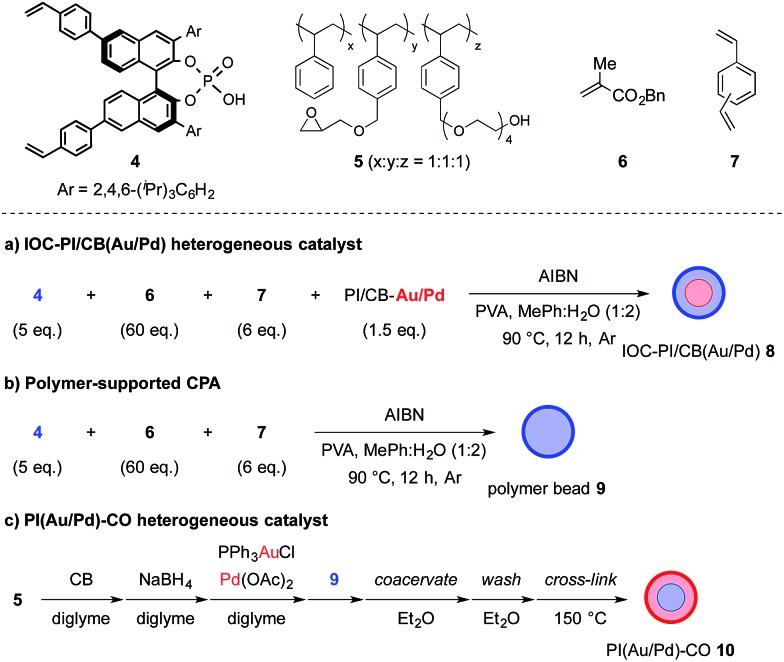
Fabrication procedures for (a) IOC–PI/CB(Au/Pd) **8**; (b) polymer-supported CPA **9**; and (c) PI(Au/Pd)–CO **10**. Blue represents the layer containing CPA while red represents the layer containing Au/Pd NPs.

**Scheme 3:**
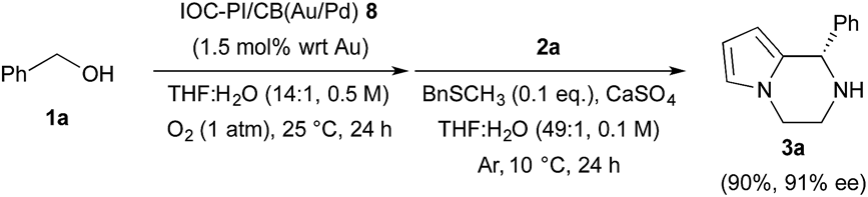
Sequential aerobic oxidation of **1a** and asymmetric aza-FC reaction with **2a** catalyzed by IOC–PI/CB(Au/Pd) **8**.

With these results in hand, we began the process of fabricating chiral bifunctional heterogeneous catalysts that would be capable of facilitating the sequential aerobic oxidation-asymmetric aza-FC reaction. We began by developing a reliable synthetic route for (*S*)-TRIP-type monomer **4**,^13^ and then utilized this as a chiral feedstock to construct the chiral composite material **8** through a pseudo-suspension co-polymerization method (Fig. 1a). With this heterogeneous bifunctional chiral catalyst in hand, we examined the asymmetric TOP between benzyl alcohol (**1a**) and *N*-aminoethylpyrrole **2a** (Scheme 3). It was found that after slight modification of the optimized reaction conditions previously determined for the combined catalyst system of PI/CB–Au/Pd and (*S*)-TRIP, the desired piperazine **3a** could be obtained in excellent yield and enantioselectivity. We also examined our model reaction using the layered heterogeneous catalyst **10** with inversed placement of the Au/Pd NPs and CPA (Fig. 1c), and it was found to catalyze the asymmetric TOP with similar results (85%, 88% ee of **3a**).

After establishing the optimal reaction conditions for the sequential aerobic oxidation-asymmetric aza-FC reaction, we examined the substrate scope for this one-pot process (Table 1). It was found that substituted benzyl alcohols **1a–h**, bearing electron-donating substituents, could be utilized for the asymmetric TOP to furnish the desired chiral piperazines **3a–h** in good yields and enantioselectivities (entries 1–8). On the other hand, when we examined 4-fluorobenzyl alcohol (**1i**) as a substrate, only a trace amount of the expected product **3i** was detected under the current reaction conditions. Control studies revealed that the origin of this problem was the aerobic oxidation step, and we found that Au/Pt NPs in DCM : H_2_O (1 : 1) were more effective for the aerobic oxidation of benzyl alcohols possessing electron-withdrawing substituents. Thus, we prepared a new chiral bifunctional heterogeneous catalyst, IOC–PI/CB(Au/Pt) **11**, and found that this new chiral composite material could effectively catalyze the sequential oxidation-asymmetric aza-FC reaction for 4-substituted benzyl alcohols with a wide range of functional groups, such as F, Cl, CF_3_, CO_2_Me and CN, to furnish the corresponding chiral piperazines **3i–n** in good yields and enantioselectivities (entries 9–14). We also examined the asymmetric TOP with methyl substituted *N*-aminoethylpyrroles **2b–c**, and it was found that these substrates were also suitable, providing chiral bicyclic heterocycles **3o–p** with high yields and enantioselectivities (entries 15 and 16).

**Table 1:** Substrate scope for the IOC–PI/CB(Au/Pd) **8** or IOC–PI/CB(Au/Pt) **11**-catalyzed asymmetric TOP between benzyl alcohols **1a–n** and *N*-aminoethylpyrroles **2a–c** ^*a*^

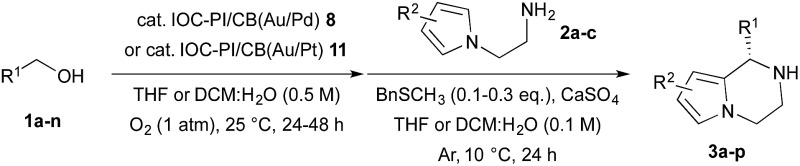				
Entry	**1**: R^1^; **2**: R^2^	**3**	Yield^*d*^ (%)	ee^*e*^ (%)
1	**1a**: Ph; **2a**: H	**3a**	89	94
2	**1b**: 4-Me–C_6_H_4_; **2a**: H	**3b**	89	84
3	**1c**: 2-Me–C_6_H_4_; **2a**: H	**3c**	91	92
4	**1d**: 4-MeO–C_6_H_4_; **2a**: H	**3d**	80	80
5^*b*^	**1e**: 3-MeO–C_6_H_4_; **2a**: H	**3e**	83	93
6^*b*^	**1f**: 2-MeO–C_6_H_4_; **2a**: H	**3f**	85	91
7	**1g**: 3,4-(OCH_2_O)–C_6_H_3_; **2a**: H	**3g**	84	73
8^*b*^	**1h**: 1-naphthyl; **2a**: H	**3h**	85	93
9^*c*^	**1i**: 4-F–C_6_H_4_; **2a**: H	**3i**	91	90
10^*c*^	**1j**: 2-F–C_6_H_4_; **2a**: H	**3j**	88	91
11^*c*^	**1k**: 4-Cl–C_6_H_4_; **2a**: H	**3k**	83	90
12^*c*^	**1l**: 4-CF_3_–C_6_H_4_; **2a**: H	**3l**	82	86
13^*c*^	**1m**: 4-CN–C_6_H_4_; **2a**: H	**3m**	89	95
14^*c*^	**1n**: 4-CO_2_Me–C_6_H_4_; **2a**: H	**3n**	84	90
15	**1a**: Ph; **2b**: 2-Me	**3o**	91	85
16	**1a**: Ph; **2c**: 2,4-(Me)_2_	**3p**	83	70

^*a*^Reaction conditions: benzyl alcohol **1** (0.3 mmol), IOC–PI/CB(Au/Pd) **8** (1.5 mol% wrt Au) in THF : H_2_O (v/v = 0.56 : 0.04 mL) under a balloon of oxygen gas at room temperature for 24 h (aerobic oxidation step). Then *N*-aminoethylpyrrole **2** (0.2 mmol), CaSO_4_ (200 mg), BnSCH_3_ (2.8 mg) and THF (1.4 mL) were added under a balloon of Ar at 10 °C for 24 h (aza-FC step).

^*b*^Standard reaction conditions except for the use of IOC–PI/CB(Au/Pd) **8** (3.0 mol% wrt Au) for 48 h (aerobic oxidation step) and BnSCH_3_ (5.6 mg) (aza-FC step).

^*c*^Standard reaction conditions except for the use of IOC–PI/CB(Au/Pt) **11** (3.0 mol% wrt Au) in DCM : H_2_O (v/v = 0.54 : 0.06 mL) (aerobic oxidation step) and BnSCH_3_ (5.6 mg) (aza-FC step).

^*d*^Isolated yield based on **2a–c** and determined by weight of the isolated product **3a–p**.

^*e*^The ee value was determined by chiral HPLC analysis.

**Scheme 4:**
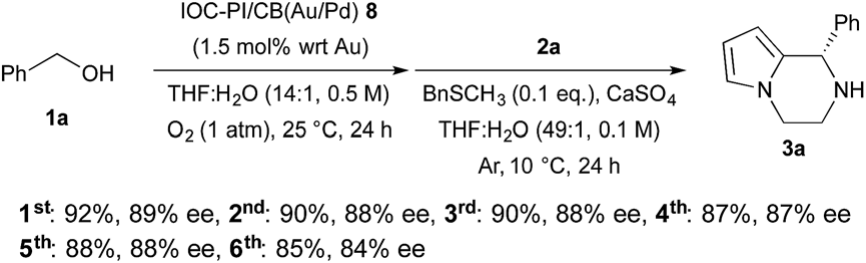
Recovery and reuse of IOC–PI/CB(Au/Pd) **8**.

Finally, we examined the possibility of recycling our chiral bifunctional heterogeneous catalyst, and found that it could be recovered and reused several times without significant loss of yield or enantioselectivity for the asymmetric TOP with benzyl alcohol **1a** and *N*-aminoethylpyrrole **2a** as substrates (Scheme 4). The key to recycling the heterogeneous catalyst was the treatment of the spent IOC–PI/CB(Au/Pd) **8** with an aqueous solution of H_2_O_2_ to remove BnSCH_3_ and reactivate the Au/Pd NP catalyst.

## Conclusions

In conclusion, we have developed a new chiral bifunctional heterogeneous catalyst, composed of metal NPs and CPAs, that was capable of facilitating the sequential one-pot aerobic oxidation-intramolecular asymmetric aza-FC reaction to provide chiral 1,2,3,4-tetrahydropyrrolo[1,2-*a*]pyrazines **3a–p** in high yields and enantioselectivities. Interestingly, we found a chemical system (H_2_O_2_/BnSCH_3_) to switch on/off the catalytic activity^14^ of the Au/Pd and Au/Pt NPs. By controlling the catalytic activity of the heterogeneous catalyst through the use of a chemical modifier, the undesired oxidation of chiral piperazines **3a–p** was avoided, and the facile reactivation of the deactivated catalyst allowed the chiral composite material to be recovered and reused several times without significant losses in yield or enantioselectivity of the desired product **3a**. Moreover, we were able to demonstrate that heterogeneous CPAs could retain their catalytic ability in the presence of metal NPs under oxidative conditions (O_2_ and H_2_O_2_). Further investigations into the use of these robust chiral heterogeneous catalysts in other types of asymmetric TOPs are currently underway in our laboratory.
