# Supplementary material for: Integration of aerobic oxidation and intramolecular asymmetric aza-Friedel–Crafts reactions with a chiral bifunctional heterogeneous catalyst
Source: Chem Sci. 2016 Oct 5;8(2):1356–9. doi: 10.1039/c6sc03849b (PMC5362052; doi:10.1039/c6sc03849b)
Supplement: Supplementary file 1 [file SC-008-C6SC03849B-s001.pdf]

Electronic Supplementary Material (ESI) for Chemical Science.
This journal is © The Royal Society of Chemistry 2016

Supporting Information

Integration of Aerobic Oxidation and Intramolecular Asymmetric aza-Friedel-Crafts Reaction with a Chiral Bifunctional Heterogeneous Catalyst

*Hong-Gang Cheng, Javier Miguélez, Hiroyuki Miyamura, Woo-Jin Yoo and Shū Kobayashi**

Department of Chemistry, School of Science, The University of Tokyo, 7-3-1, Hongo, Bunkyo-ku, Tokyo, 113-0033, Japan

Contents

1. General Information **S-2**

2. Optimization of the Sequential Aerobic Oxidation-Asymmetric *aza*-Friedel Crafts Reaction Using a Homogeneous Phosphoric Acid Catalyst **S-3**

3. Preparation and Characterization Data of the Chiral Bifunctional Catalyst **S-7**

   3.1 General Procedure for the Preparation of the CPA Monomer **S-7**
   3.2 Characterization Data **S-8**
   3.3 General Procedure for the Preparation of the Chiral Bifunctional Catalyst **S-9**
   3.4 Optimization of Reaction Conditions with IOC-PI/CB(Au/Pd) **8** **S-11**
   3.5 STEM and EDS Images of IOC-PI/CB(Au/Pd) **8** **S-12**

4 General Procedure for the Asymmetric TOP using the Chiral Bifunctional Catalyst **S-13**

   4.1 General Procedure for the Asymmetric TOP with IOC-PI/CB(Au/Pd) **8** **S-13**
   4.2 General Procedure for the Asymmetric TOP with IOC-PI/CB(Au/Pt) **11** **S-13**
   4.3 Characterization Data **S-14**
   4.4 Recovery and Reuse Studies **S-18**

5. Copies of the ^{1}H and ^{13}C NMR Spectra **S-20**

6. Copies of the HPLC Chromatograms **S-40**

7. References **S-48**

1. General Information

Unless otherwise noted, commercial reagents were purchase from Tokyo Chemical Industry Co. Ltd and purified by distillation or recrystallization before use. All organic solvents were purified by distillation under dry argon atmosphere or purchased as anhydrous solvent from Wako Pure Chemical Industries, Ltd. ^{1}H, ^{13}C and ^{31}P NMR spectra were recorded on a JEOL ECX-400, JEOL ECX-500 or JEOL ECX-600 in $CDCl_3$. Chemical shifts of ^{1}H and ^{13}C were reported in parts per million (ppm) from tetramethylsilane using the solvent resonance as the internal standard ($CDCl_3$: 7.24 ppm for ^{1}H NMR and 77.0 ppm for ^{13}C NMR). Chemical shifts of ^{31}P were reported in parts per million (ppm) from H_3PO_4 using triphenylphosphine oxide (23.0 ppm) as the internal standard. IR spectra were measured on a JASCO FT/IR-610 spectrometer. High-resolution mass spectrometry was carried out using a JEOL JMS-T100TD (ESI and DART). Column chromatography and preparative thin-layer chromatography (PTLC) were carried out using Wako gel B-5F from Wako Pure Chemical Industries, Ltd. or Merck & Co. (normal type). Enantiomeric ratios were determined by chiral HPLC with chiral columns (chiralpak AS-H column, chiralpak AD-H column, chiralpak OJ-H column, chiralpak IC-H column or chiralcel OD-H column) with hexane and ^{i}PrOH as solvents. Optical rotations were measured with a polarimeter. Inductively Coupled Plasma (ICP) analysis was performed on Shimadzu ICPS-7510 equipment.

2. Optimization of the Sequential Aerobic Oxidation-Asymmetric aza-Friedel Crafts Reaction Using a Homogeneous Phosphoric Acid Catalyst

In order to evaluate the possibility of integrating an aerobic oxidation process with a FC reaction, we examined the TOP between benzyl alcohol (**1a**) and *N*-aminoethylpyrrole **2a** using PI/CB-Au/Pd and *p*-toluenesulfonic acid as co-catalysts (Scheme 1S, eqn 1).

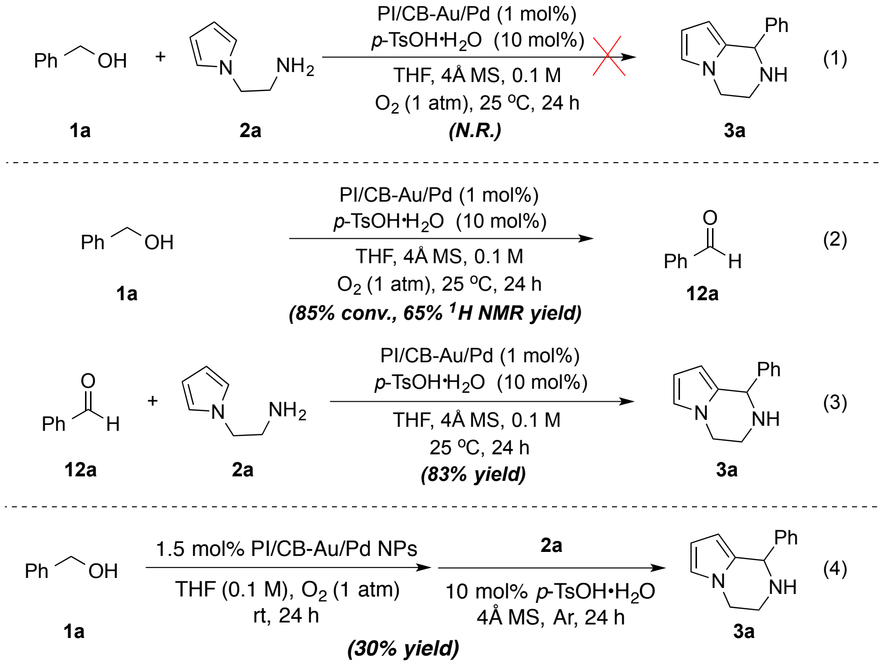

Scheme 1S. Initial Attempt and Control Experiments for the Sequential Oxidation-FC Process

However, the desired product **3a** was not observed. We performed control experiments and it was found that the aerobic oxidation of **1a** occurs with a moderate yield (Scheme 1S, eqn 2) and the *aza*-FC reaction occurs in a good yield (Scheme 1S, eqn 3) under our initial reaction conditions. Since the individual steps occur under the initial reaction conditions, it was assumed that the starting reagents might not be compatible to the two catalysts used in the reaction system. As such, when the reaction was performed in a one-pot, stepwise manner, the desired cyclic amine **3a** was obtained in a low yield (Scheme 1S, eqn 4). This result confirmed our initial suspicion and began the optimization process for this TOP.

Since we were able to obtain only moderate yield of the desire aldehyde **12a**, we began by optimizing the aerobic oxidation step (Table 1S).

Table 1S. Optimization of the Aerobic Oxidation of **1a** with PI/CB-Au/Pd[a]

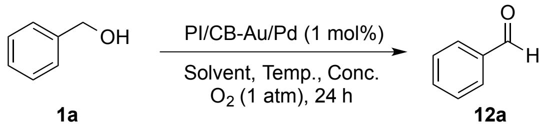

Entry	Solvent	Temp. (°C)	Conc.	Conv.[b]	Yield (%)[b]
1	THF	25	0.1 M	30	25

2	THF	25	1.0 M	49	45
3	THF	60	1.0 M	100	59
4	THF:H_2O (v:v = 1:1)	25	1.0 M	100	85
5	***THF:H_2O (v:v = 9:1)***	***25***	***1.0 M***	***99***	***85***

[a] Unless otherwise noted, reactions were carried out with **1a** (0.30 mmol) and PI/CB-Au/Pd (1 mol% wrt Au) under a balloon of oxygen gas. [b] Determined by GC analysis, with dodecane as internal standard.

It was found that by increasing the concentration of the solvent, in conjugation with using an aqueous THF solution provided the desired aldehyde **12a** in an excellent yield (entry 5). Once we optimized the oxidation step of the sequential reaction, we focused on the TOP using *(S)*-TRIP as a CPA for the asymmetric *aza*-FC reaction (Table 2S).

Table 2S. Optimization for the Tandem Aerobic Oxidation-Asymmetric *aza*-FC Reaction with *(S)*-TRIP as the CPA Catalyst[a]

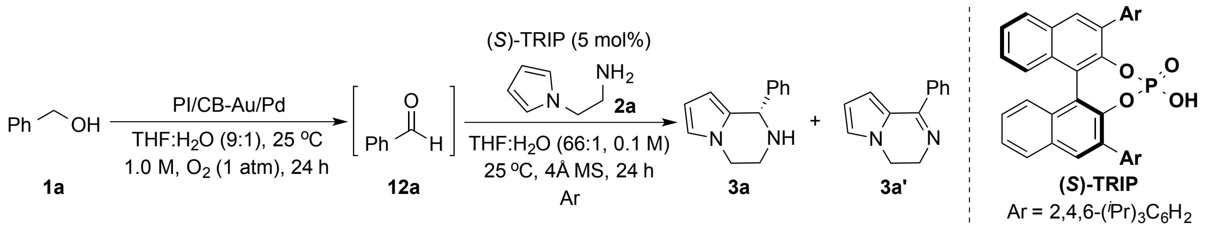

Entry	Ratio (**1a**/**2a**)	Ratio (**3a**/**3a'**)[b]	4Å MS	Yield of **3a** (%)[c]	Ee of **3a** (%)[d]
1	1.5:1	4:1	100 mg	72	91
2	1.2:1	5.5:1	100 mg	74	88
3	1:1	>95:5	100 mg	75	84
4	1:1.2	>95:5	100 mg	68	80
5	1:1.5	>95:5	100 mg	70	75
6	1.5:1	3.3:1	-	70	90
7	***1.5:1***	***4.5:1***	***200 mg***	***76***	***93***

[a] Unless otherwise noted, reactions were carried out with **1a** (0.2-0.3 mmol), **PI/CB-Au/Pd** in THF:H_2O (v:v = 0.27:0.03 mL) under a balloon of oxygen at 25 °C for 24 h, (aerobic oxidation step). Then **(*S*)-TRIP** (5 mol%), **2a** (0.2-0.3 mmol), 4Å MS (0-200 mg) and THF (1.7 mL) were added under a balloon of Ar at 25 °C for 24 h (*aza*-FC step). [b] Determined by crude ^{1}H NMR. [c] Yield of isolated product. [d] The ee values were determined by chiral HPLC analysis.

We examined the effect of the ratio of reagents and the use of molecular sieves, and we were able to determine using an excess of **1a** and 200 mg of 4Å MS provided the desired product in good yield and excellent enantioselectivity (entry 7). While the yield of the asymmetric TOP was somewhat acceptable, we found that **3a** can also undergo oxidation to generate the undesired by-product **3a'**. A hint to overcome this problem was revealed in our optimization studies. It was found that when an excess of the *N*-aminoethylpyrrole **2a** was used, the formation of **3a'** was minimized (entries 3-5). Therefore, we hypothesized **2a** may act as a catalyst poison to the Au/Pd NPs and we believed that the introduction of Lewis basic additives could improve the overall reaction. We tested this hypothesis by examining the effect of Lewis basic compounds for the oxidation of **3a** (Table 3S) and found that both PPh_3 (entry 4) and BnSMe (entry 6) could be used to completely suppress this oxidation process.

Table 3S. Effect of Lewis Basic Additives for the Conversion of **3a** to **3a'**[a]

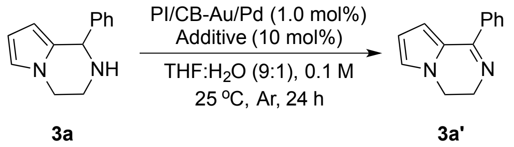

Entry	Additive	Conv. (%)[b]	Yield (%)[b]
1	Amine **2a**	0	0
2	$PhCONH_2$	32	24
3	DMF	25	19
4	***PPh_3***	***0***	***0***
5	TMEDA	11	7
6	***$BnSCH_3$***	***0***	***0***

[a] Unless otherwise noted, reactions were carried out with **3a** (0.30 mmol), **PI/CB-Au/Pd** (1 mol% wrt Au) in THF:H_2O (v:v = 2.7:0.3 mL) at 25 °C under a balloon of Ar. [b] Determined by crude ^{1}H NMR.

With these results in hand, we examined the effect of these two additives for the asymmetric TOP (Table 4S). It was found that both additives improved the yield of **3a** significantly and minimized the formation of **3a'**. However, we chose $BnSCH_3$ as the additive since the use of PPh_3 led to significant metal leaching (entry 2).

Table 4S. Sequential Aerobic Oxidation-Asymmetric aza-FC Reaction with Additives[a]

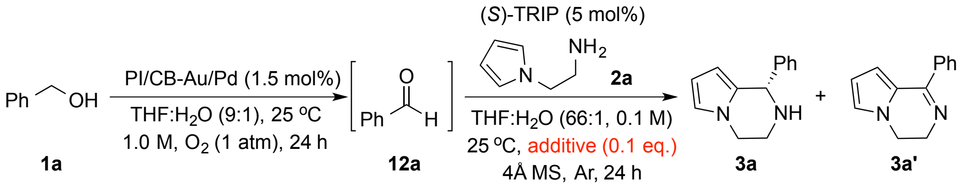

Entry	Additive	Ratio (**3a**/**3a'**)[b]	Yield of **3a** (%)[c]	Ee of **3a** (%)[d]	Metal Leaching (Au/Pd)[e]
1	PPh_3	>95:5	91	93	8.4%/5.5%
2	***$BnSCH_3$***	***>95:5***	***90***	***92***	***ND[f]/0.22%***

[a] Unless otherwise noted, reactions were carried out with **1a** (0.3 mmol), **PI/CB-Au/Pd** (1.5 mol% wrt Au) in THF:H_2O (v:v = 0.27:0.03 mL) under a balloon of oxygen at 25 °C for 24 h (aerobic oxidation step). Then **(*S*)-TRIP** (5 mol%), **2a** (0.2 mmol), 4Å MS (200 mg), additive (0.02 mmol) and THF (1.7 mL) were added under a balloon of Ar at 25 °C for 24 h (*aza*-FC step). [b] Determined by crude ^{1}H NMR. [c] Yield of isolated product. [d] The ee values were determined by chiral HPLC analysis. [e] Detected with ICP analysis. Detection limit: Au/Pd = 0.009/0.008 ppm. [f] ND = below the detection limit of the ICP equipment.

While we were able to determine the optimized reaction conditions for the asymmetric TOP using a homogeneous CPA as a co-catalyst, in our actual system using a bifunctional catalyst, we need to separate 4Å MS if we wish to recycle the heterogeneous catalyst. Therefore, we examined possible water-soluble dehydrating reagents as replacements for 4Å MS (Table 5S).

Table 5S. Investigation of Various Dehydrating Reagents for the *aza*-FC Reaction[a]

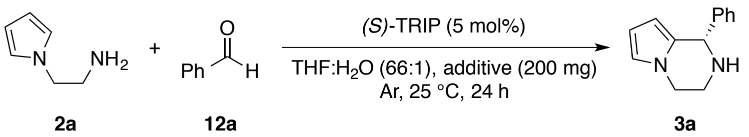

Entry	Additive	Yield (%)[b]	Ee (%)[c]
1	4Å MS	90	93
2	Na_2SO_4	87	89
3	$MgSO_4$	89	90
4	$CaCl_2$	92	57
5[d]	$MgSO_4$	88	91
6	***$CaSO_4$***	***94***	***92***

[a] Unless otherwise noted, reactions were carried out with **2a** (0.2 mmol), **12a** (0.3 mmol), **(*S*)-TRIP** (5 mol%), additive (200 mg) in THF:H_2O (v:v = 1.97:0.03 mL) at 25 °C under a balloon of Ar. [b] Isolated yield. [c] The ee values were determined by chiral HPLC analysis. [d] Add 300 mg of $MgSO_4$.

Among the various dehydrating reagents tested, $CaSO_4$ provided the best result (entry 6) and was selected as an additive for the asymmetric TOP catalyzed by a chiral bifunctional heterogeneous catalyst.

In addition, we also examined the possibility of performing the tandem process using two separate solid catalysts, PI/CB-Au/Pd and the immobilized *(S)*-TRIP (Scheme 2S). It was found that the tandem process occurs well to provide the desired amine in similar results to the bifunctional catalyst. However, it should be noted that our bifunctional heterogeneous catalyst may be more reliable for recycling the catalyst since the ratio of the Au/Pd NPs and the CPA will be constant throughout the reuse and recovery cycle.

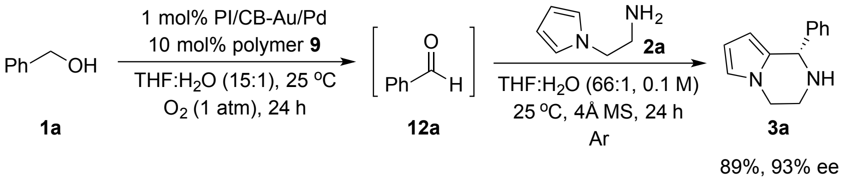

Scheme 2S. The Sequential Oxidation-asymmetric aza-FC Process by Utilizing Two Separate Immobilized Catalyst

3. Preparation and Characterization Data of the Chiral Bifunctional Heterogeneous Catalyst

3.1 General Procedure for the Preparation of the CPA Monomer

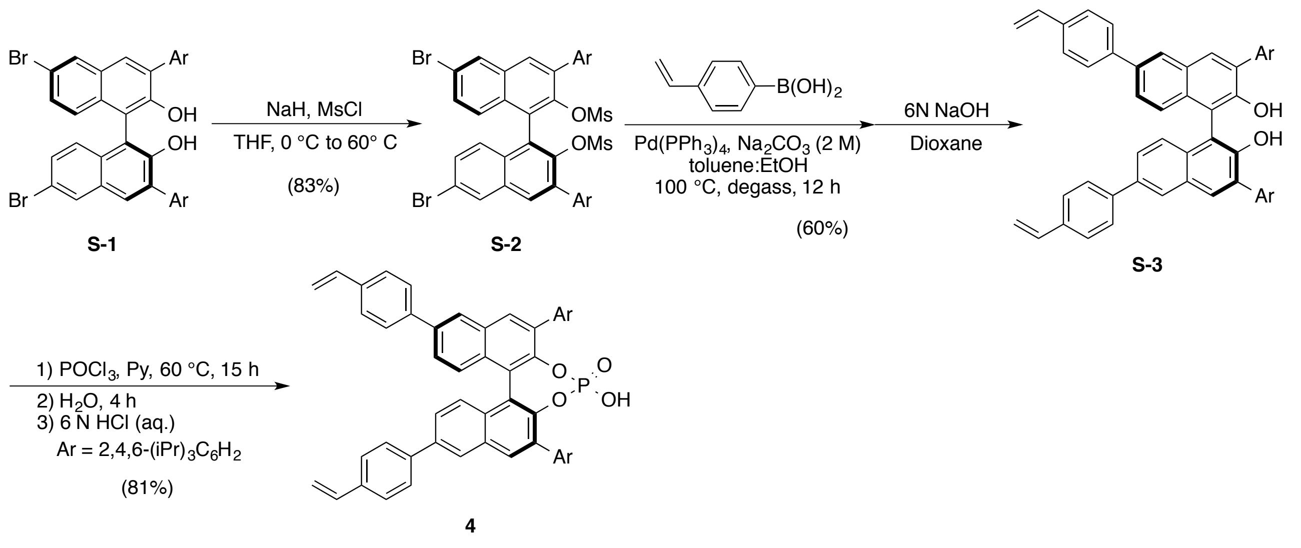

Scheme 3S. Synthesis of CPA Monomer **4**

Compound **S-1**[1] and *p*-vinylphenylboronic acid[2] were prepared according to literature procedures.

Synthesis of Intermediate S-2

To a solution of diol **S-1** (1.6 g, 1.9 mmol) in THF (15 mL) at 0 °C was added NaH (55% w/w, 33 mg, 0.76 mmol). The reaction mixture was stirred for 30 min at 0 °C, and then MsCl (400 μL, 5.7 mmol) was added. Next, the reaction mixture was stirred at 60 °C for 12 h, then was allowed to cool to room temperature. The mixture was quenched with saturated aqueous $NaHCO_3$ solution, and then extracted with EtOAc. The combined organic fractions were washed with brine, dried over Na_2SO_4, filtered, and finally concentrated under reduced pressure. The crude reaction mixture was purified by column chromatography (eluent: 10% DCM in hexane) to provide **S-2** as a yellow foam (83%).

Synthesis of Intermediate S-3

To a solution of aryl bromide **S-2** (500 mg, 0.50 mmol) in MePh (2 mL) and EtOH (1 mL) was added an aqueous solution of Na_2CO_3 (2M, 1 mL), followed by the addition of $Pd(PPh_3)_4$ (60 mg, 0.05 mmol) and *p*-vinylphenylboronic acid (400 mg, 2.7 mmol). The reaction mixture was degassed, then heated at 100 °C for 24 h. After cooling to room temperature, the reaction mixture was extracted with DCM and the combined organic extracts were dried over Na_2SO_4 and concentrated under reduced pressure. The crude product was then dissolved in dioxane (15 mL) and a solution of NaOH (6 N, 15 mL) was then added. After stirring the reaction mixture at 60 °C overnight, the reaction was allowed to cool to room temperature. The reaction was quenched with $NaHCO_3$ until the pH of the mixture was approximately 9, then was extracted with EtOAc, dried over Na_2SO_4, and concentrated under reduced pressure. The crude residue was purified by column chromatography (eluent: 5% DCM in hexane) to provide diol **S-3** (60% over two steps).

Synthesis of Intermediate S-4

In a flame-dried round bottom flask, monomeric diol **S-3** (1.5 g, 1.67 mmol) was dissolved in pyridine (22 mL) under an Ar atmosphere. To this solution was added phosphrous oxychloride (1.28 g, 8.35 mmol) and the reaction mixture was stirred at 60 °C for 15 h. After cooling to room temperature, H_2O (11 mL) was added and the resulting suspension was stirred at 60 °C for an additional 4 h. After cooling to room temperature, the reaction mixture was poured into aqueous HCl (6 N, 100 mL) and extracted with DCM. The organic fraction was washed with aqueous HCl (6 N), dried over Na_2SO_4, and concentrated under reduced pressure. The crude was purified by column chromatography (eluent: 1% MeOH in DCM) and the resulting white solid was dissolved in DCM, washed with aqueous HCl (6 N), dried over Na_2SO_4, and concentrated under reduced pressure to provide CPA monomer **4** (81%).

3.2 Characterization Data

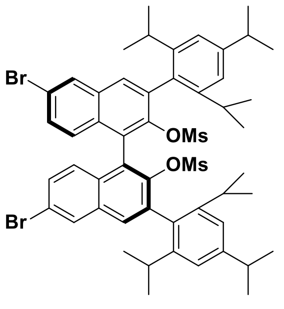

(1*S*,3'*s*)-6,6'-Dibromo-3,3'-bis(2,4,6-triisopropylphenyl)-[1,1'-binaphthalene]-2,2'-diyl dimethanesulfonate (S-2)

^{1}H NMR (500 MHz, $CDCl_3$) δ 7.87-7.86 (m, 2H), 7.67 (d, *J* = 4.8 Hz, 2H), 7.39-7.36 (m, 2H), 7.22 (d, *J* = 1.3 Hz, 2H), 7.13-7.09 (m, 4H), 3.79-3.64 (m, 6H), 2.92-2.90 (m, 6H), 2.47-2.39 (m, 24H), 2.36 (d, *J* = 5.0 Hz, 6H), 2.15-2.13 (m, 6H). **^{13}C NMR** (125 MHz, $CDCl_3$) δ 149.88, 148.53, 148.13, 145.42, 134.03, 132.68, 131.65, 131.28, 131.01, 130.20, 129.92, 129.15, 127.14, 121.27, 121.02, 120.76, 38.02, 34.39, 30.69, 30.56, 26.78, 25.25, 24.17, 23.20, 23.09. MS (EI) m/z = 1004.2992. HRMS (DART) calcd for $C_{52}H_{61}Br_2O_6S_2$ ($[M+H])^+$:1003.2276; found 1003.2272. IR (KBr) ν = 796, 944, 1163, 1368, 1465, 2360, 2874, 2964, 3524 cm^{-1}.

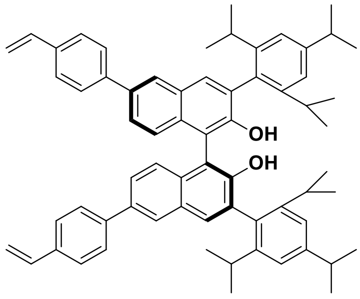

(1*S*,3'*s*)-3,3'-Bis(2,4,6-triisopropylphenyl)-6,6'-bis(4-vinylphenyl)-[1,1'-binaphthalene]-2,2'-diol (S-3)

^{1}H NMR (600 MHz, $CDCl_3$) δ 8.00 (s, 2H), 7.76 (s, 2H), 7.60 (d, *J* = 8.2 Hz, 4H), 7.54-7.52 (m, 2H), 7.43 (d, *J* = 8.2 Hz, 4H), 7.33 (d, *J* = 8.8 Hz, 2H), 7.09-7.07 (m, 4H), 6.71-6.67 (m, 2H), 5.72 (d, *J* = 17.6 Hz, 2H), 5.20 (d, *J* = 10.9 Hz, 2H), 4.90 (s, 2H), 2.90 (dt, *J* = 13.7, 6.8 Hz, 2H), 2.82 (dt, *J* = 13.7, 6.8 Hz, 2H), 2.66 (dt, *J* = 13.5, 6.7 Hz, 2H), 1.25 (d, *J* = 6.9 Hz, 12H), 1.15 (d, *J* = 6.7 Hz, 6H), 1.07-1.03 (m, 12H), 0.98 (d, *J* = 6.8 Hz, 6H). **^{13}C NMR** (150 MHz, $CDCl_3$) δ 150.72, 149.29, 147.85, 147.79, 140.43, 136.44, 136.39, 136.17, 132.71, 130.84, 130.07, 129.60, 129.25, 127.32, 126.70, 126.14, 125.99, 125.15, 121.32, 121.25, 113.83, 113.21, 34.34, 30.93, 30.85, 24.34, 24.32, 24.06, 23.99, 23.96, 23.74. HRMS (ESI) calcd for $C_{66}H_{69}O_2$ $[M-H]^+$:893.5298; found 893.5295. IR (KBr) ν = 740, 824, 1439, 1496, 1604, 2961, 3519 cm^{-1}.

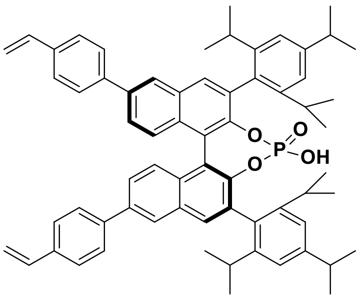

(2*s*,11b*S*)-4-Hydroxy-2,6-bis(2,4,6-triisopropylphenyl)-9,14-bis(4-vinylphenyl)dinaphtho[2,1-d:1',2' f][1,3,2]dioxaphosphepine 4-oxide (4)

^{1}H NMR (600 MHz, $CDCl_3$) δ 8.00 (s, 2H), 7.81 (s, 2H), 7.62 (d, *J* = 8.2 Hz, 4H), 7.55 (d, *J* = 8.9 Hz, 2H), 7.46 (d, *J* = 8.2 Hz, 4H), 7.38 (d, *J* = 8.8 Hz, 2H), 6.89 (s, 4H), 6.71 (dd, *J* = 17.5, 10.9 Hz, 2H), 5.75 (d, *J* = 17.7 Hz, 2H), 5.23 (d, *J* = 10.9 Hz, 2H), 2.80-2.76 (m,

2H), 2.56-2.53 (m, 4H), 1.18-1.15 (m, 12H), 1.01 (d, $J = 5.7$ Hz, 6H), 0.95 (d, $J = 6.1$ Hz, 6H), 0.86 (d, $J = 6.7$ Hz, 6H), 0.74 (s, 6H). **^{13}C NMR** (150 MHz, $CDCl_3$) δ 148.23, 147.77, 147.15, 139.89, 137.91, 136.88, 136.28, 132.86, 131.45, 131.23, 127.95, 127.40, 126.80, 125.71, 125.62, 121.86, 121.20, 120.23, 114.15, 34.15, 30.96, 30.73, 26.41, 25.00, 24.03, 23.38, 23.17. **^{31}P NMR** (243 MHz, $CDCl_3$) δ 2.11. HRMS (ESI) calcd for $C_{66}H_{68}O_4P$ $[M-H]^+$:955.4855; found 955.4838. IR (KBr) ν = 743, 764, 1021, 1265, 1495, 1605, 2870, 2930 cm^{-1}.

3.3 General Procedure for the Preparation of the Chiral Bifunctional Catalyst

The polymer-incarcerated Au/M NPs used in these studies were prepared according to known literature procedures.[3]

Synthesis of IOC-PI/CB(Au/Pd) 8

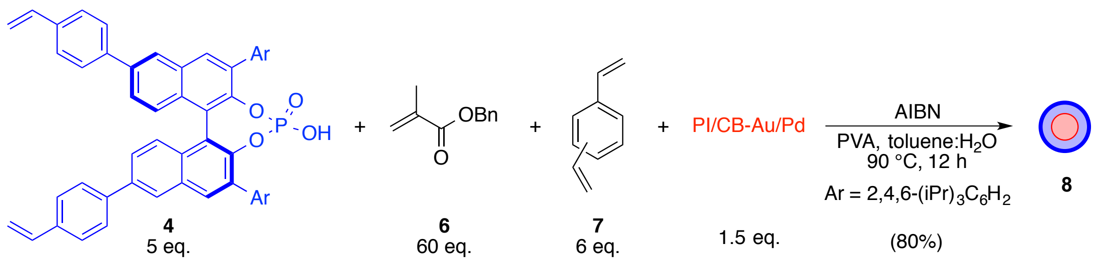

In a 20 mL test tube, chiral monomer **4** (191 mg, 0.2 mmol), benzyl methacrylate **6** (423.0 mg, 2.4 mmol), divinyl benzene **7** (62.5 mg, 0.48 mmol), PI/CB-Au/Pd (Au/Pd: 0.234/0.218 mmol/g, 256.0 mg, 0.06 mmol), poly(vinyl alcohol) (19.2 mg) and 2,2'-azo bisisobutyronitrile (AIBN) (19.2 mg) were dissolved/suspended in a MePh:H_2O solvent mixture (v:v = 1.6:3.2 mL). The solution was degassed and heated to 90 ºC under an atmosphere of Ar. After 12 h, the suspension was allowed to cool to room temperature and the solid materials were collected by vacuum filtration. The cross-linked polymer was washed successively with water, MeOH, and DCM, and dried overnight under high vacuum to provide IOC-PI/CB (Au/Pd) **8** as a greyish black powder (785.2 mg). The loadings of Au (0.063 mmol/g), Pd (0.059 mmol/g), and P (0.22 mmol/g) were determined by ICP analysis.

Polymer-supported CPA 9

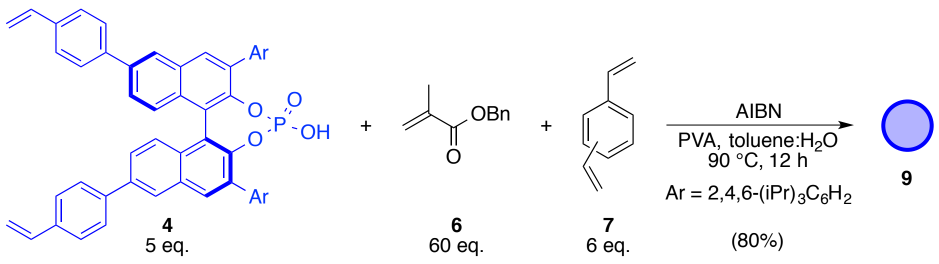

In a 10 mL test tube, chiral monomer **4** (48 mg, 0.05 mmol), benzyl methacrylate **6** (106 mg, 0.6 mmol), divinyl benzene **7** (18 mg, 0.12 mmol), poly(vinyl alcohol) (10 mg) and 2,2'-azo bisisobutyronitrile (AIBN) (4.8 mg) were dissolved/suspended in a MePh:H_2O solvent mixture (v:v = 1: 2 mL). The solution was degassed and heated to 90 ºC under an atmosphere of Ar. After 12 h,

the suspension was allowed to cool to room temperature and the solid materials were collected by vacuum filtration. The cross-linked polymer was washed successively with acetone and DCM, and dried overnight under high vacuum to provide CPA-based polymer **9** as a white powder (129 mg). The loadings of P (0.27 mmol/g) were determined by ICP analysis.

Synthesis of PI(Au/Pd)-CO 10

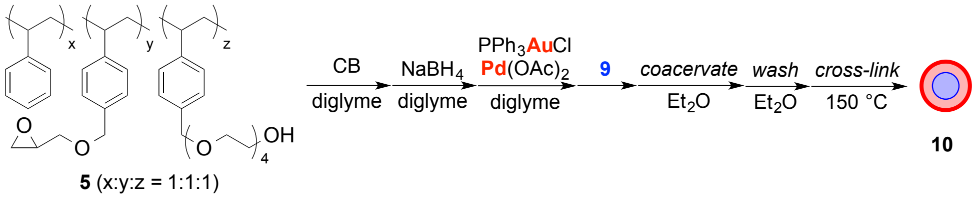

To a solution of polymer **5** (150 mg) in diglyme (5 mL) was added carbon black (150 mg) and $NaBH_4$ (18 mg, 0.48 mmol). After stirring for 5 min, a solution of PPh_3AuCl (24 mg, 0.048 mmol) and $Pd(OAc)_2$ (11 mg, 0.048 mmol) in THF (1 mL) was added dropwise and stirred for 12 h. Next, the CPA polymer **9** (120 mg) was added to the NP solution and was allowed to stir for 1 h. Then, Et_2O (50 mL) was added dropwise and the resulting solid was filtered and washed with Et_2O. The collected material was dried under high vacuum for 1h, then heated at 150 °C under an Ar atmosphere for 6 h. Next, the cross-linked polymer was suspended in THF (5 mL) and HCl (3 N, 5 mL) and was stirred overnight at room temperature. The suspension was filtered and washed with water, THF, DCM, and acetone, and the resulting polymer was dried under high vacuum for 24 h to provide PI(Au/Pd)-CO **10** (389 mg). The loadings of Au (0.099 mmol/g), Pd (0.092 mmol/g), and P (0.083 mmol/g) were determined by ICP analysis.

Synthesis of IOC-PI/CB(Au/Pt) 11

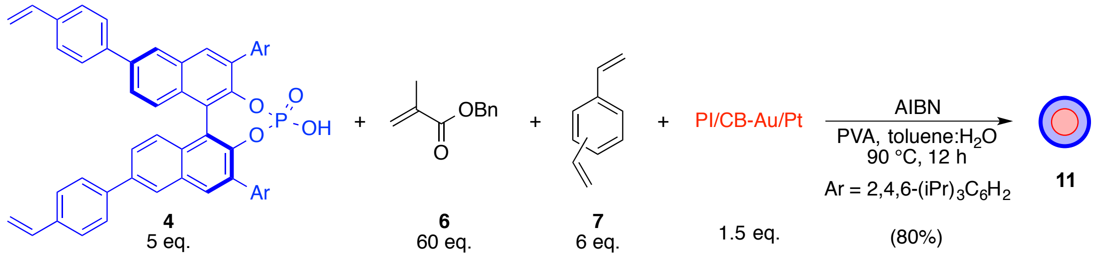

In a 20 mL test tube, chiral monomer **4** (191 mg, 0.2 mmol), benzyl methacrylate **6** (423.0 mg, 2.4 mmol), divinyl benzene **7** (62.5 mg, 0.48 mmol), PI/CB-Au/Pt (Au/Pt: 0.163/0.258 mmol/g, 368 mg, 0.06 mmol), poly(vinyl alcohol) (19.2 mg) and 2,2'-azo bisisobutyronitrile (AIBN) (19.2 mg) were dissolved/suspended in a MePh:H_2O solvent mixture (v:v = 1.6:3.2 mL). The solution was degassed and heated to 90 ºC under an atmosphere of Ar. After 12 h, the suspension was allowed to cool to room temperature and the solid materials were collected by vacuum filtration. The cross-linked polymer was washed successively with water, MeOH, and DCM, and dried overnight under high vacuum to provide IOC-PI/CB (Au/Pt) **11** as a greyish black powder (829 mg). The loadings of Au (0.065 mmol/g), Pt (0.103 mmol/g), and P (0.19 mmol/g) were determined by ICP analysis.

3.4 Optimization of Reaction Conditions with IOC-PI/CB(Au/Pd) **8**

We examined the influence of the co-monomers for the asymmetric TOP and prepared various chiral bifunctional catalysts following the method described for IOC-PI/CB(Au/Pd) **8** (Table 6S). Based on these results, we further optimize the reaction conditions for the asymmetric TOP using IOC-PI/CB(Au/Pd) **8**.

Table 6S. Preparation and Evaluation of Chiral Bifunctional Catalysts[a]

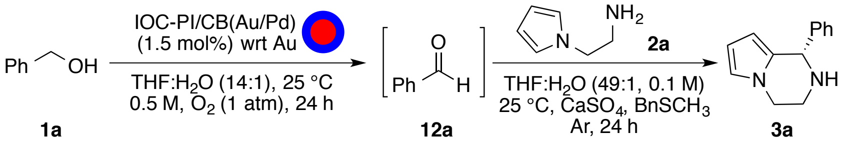

Entry	IOC-PI/CB(Au/Pd)	Loadings of Au/Pd/P (mmol/g)	Co-monomer	Yield (%)[b]	Ee (%)[c]
1	**8**	0.063/0.059/0.22	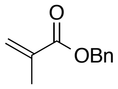	89	88
2	**8'**	0.11/0.090/0.26	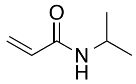	88	82
3	**8''**	0.10/0.091/0.23	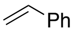	65	70

[a] Unless otherwise noted, reactions were carried out with **1a** (0.3 mmol), IOC-PI/CB(Au/Pd) (1.5 mol% wrt Au) in THF:H_2O (v:v = 0.27:0.03 mL) under a balloon of oxygen at 25 °C for 24 h (aerobic oxidation step). Then **2a** (0.2 mmol), $CaSO_4$ (200 mg), $BnSCH_3$ (2.8 mg) and THF (1.7 mL) were added under a balloon of Ar at 25 °C for 24 h (*aza*-FC step). [b] Isolated yield. [c] The ee values were determined by chiral HPLC analysis.

Since the yield and ee of chiral piperazine **3a** using IOC-PI/CB(Au/Pd) **8** as a catalyst was slightly lower than the results obtained using PI/CB-Au/Pd and *(S)*-TRIP as co-catalysts, we optimized the reaction temperature for the *aza*-FC step (Table 7S).

Table 7S. Screening of Temperature of Asymmetric *aza*-Friedel-Crafts Reaction[a]

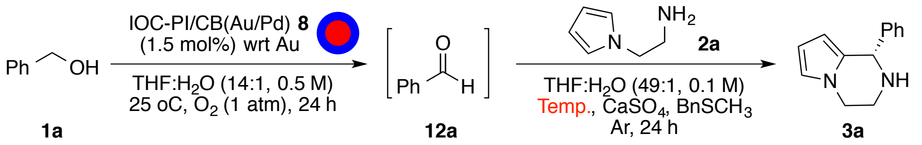

Entry	Temp. (°C)	Yield (%)[b]	Ee (%)[c]
1	35	88	84
2	30	90	87
3	25	88	89
4	20	88	90
5	15	89	91
6	***10***	***90***	***91***
7	5	87	83
8	0	84	77
9[d]	10	90	91
10[d]	5	91	90
11[d]	0	87	88

[a] Unless otherwise noted, reactions were carried out with **1a** (0.3 mmol), IOC-PI/CB(Au/Pd) **8** (1.5 mol%, wrt Au) in THF:H_2O (v:v = 0.56:0.04 mL) under a balloon of oxygen at 25 °C for 24 h (aerobic oxidation

step). Then **2a** (0.2 mmol), $CaSO_4$ (200 mg), $BnSCH_3$ (2.8 mg) and THF (1.4 mL) were added under a balloon of Ar at the corresponding temp., for 24 h (*aza*-FC step). [b] Isolated yield based on **2a** and determined by weight of the isolated product **3a**. [c] The ee values were determined by chiral HPLC analysis. [d] Stirred at the corresponding temp. for 48 h (*aza*-FC step).

It was found that the temperature had obvious effect on the enantioselectivity. Generally speaking, when the reaction was conducted at a higher temperature, for example, 35 °C, some drop in the enantioselectivity was observed (entry 1). In contrast, when the reaction was conducted at a lower temperature, higher enantioselectivities were observed (entries 1-6). When the reaction was conducted at 10 °C, we can get the best enantioselectivity (91% ee, entry 6). However, when the reaction was conducted at 5 or 0 °C, only 83% and 77% ees were obtained, respectively (entries 7-8). We speculated that at lower temperatures, the *aza*-FC reaction was slow, and during purification by chromatography, some background cyclization occurred to lower the observed enantioselectivity. To demonstrate this issue, we prolonged the reaction time to 48 h. It was found that, for the reaction that occurred at 10 °C, no difference was observed (entry 9 vs. entry 6). However, for the reactions at 5 or 0 °C, obvious increase in enantioselectivities were observed (entries 10-11 vs. entries 7-8).

3.5 STEM and EDS Images of IOC-PI/CB(Au/Pd) **8**

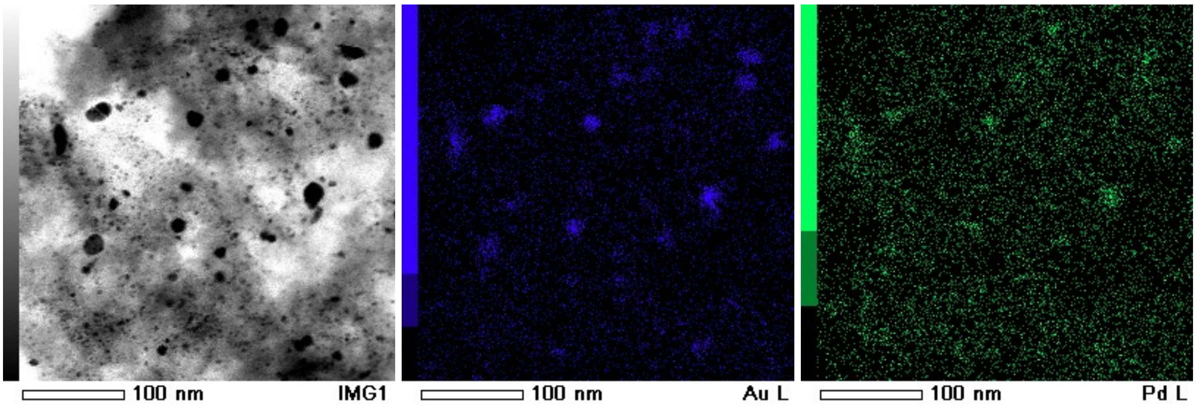

Figure 1S. STEM and EDS Images of IOC-PI/CB(Au/Pd) **8**

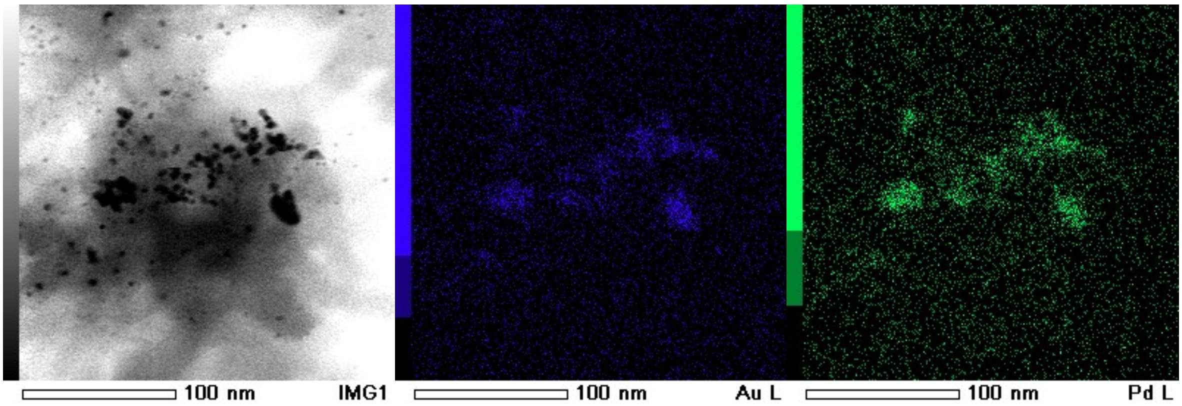

Figure 2S. STEM and EDS Images of the Recovered IOC-PI/CB(Au/Pd) **8**

4. General Procedure for the Asymmetric TOP using the Chiral Bifunctional Catalyst

4.1 General Procedure for the Asymmetric TOP with IOC-PI/CB(Au/Pd) 8

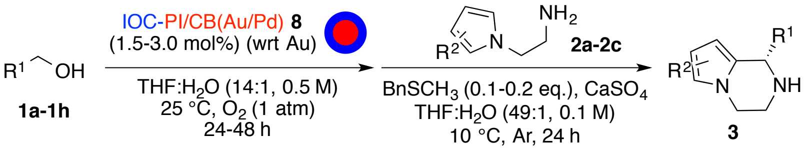

To a Carousel tube was charged benzyl alcohol **1a** (32.4 mg, 0.30 mmol), IOC-PI/CB(Au/Pd) **8** (47.6 mg, 0.003 mmol wrt Au), THF (560 µL) and H_2O (40 µL). The carousel tube was flushed with O_2 gas and the reaction mixture was stirred at 25 ºC for 24 hours under a balloon pressure of O_2. Then, *N*-aminoethylpyrrole **2a** (22.0 mg, 0.2 mmol), $CaSO_4$ (200 mg), $BnSCH_3$ (2.8 mg, 0.02 mmol), and THF (1.4 mL) were added in sequence. The carousel tube was then flushed with Ar gas and the mixture was stirred at 10 ºC for an additional 24 hours. After completion of the asymmetric TOP, the heterogeneous catalyst **8** and $CaSO_4$ were separated via vacuum-filtration and subsequently washed with THF (20 mL). All solvents were removed under reduced pressure and the crude reaction mixture was purified by PTLC using EtOAc as the eluent to provide chiral piperazine **3a** (35.8 mg, 90% yield, 91% ee) as a white solid.

4.2 General Procedure for the Asymmetric TOP with IOC-PI/CB(Au/Pt) 11

During the course of our studies, it was found that the asymmetric TOP could not be performed with IOC-PI/CB(Au/Pd) **8** with benzyl alcohols bearing electron-withdrawing substituents. The problem stem from the inability of **8** to efficiently mediate the aerobic oxidation. Thus, we re-optimized the aerobic oxidation reaction (Table 8S).

Table 8S. Optimization of the Aerobic Oxidation Reaction of 4-Fluorobenzyl Alcohol[a]

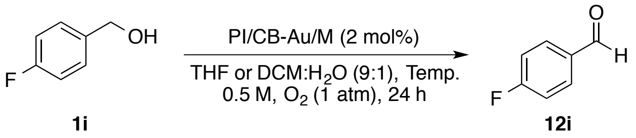

Entry	PI/CB-Au/M	Solvent	Temp. (°C)	Time (h)	Conv. (%)[b]	Yield (%)[b]
1	PI/CB-Au/Pd	$THF:H_2O$	25	24	31	23
2	PI/CB-Au/Pd	$THF:H_2O$	60	24	100	53
3	PI/CB-Au/Pt	$THF:H_2O$	60	24	100	75
4	PI/CB-Au/Pd	$DCM:H_2O$	25	24	85	78
5	***PI/CB-Au/Pt***	***$DCM:H_2O$***	***25***	***24***	***96***	***89***

[a] Unless otherwise noted, reactions were carried out with **1i** (0.3 mmol), **PI/CB-Au/M** (2 mol%, wrt Au) in THF or $DCM:H_2O$ (9:1, 0.6 mL) under a balloon of oxygen for 24 h. [b] Determined by GC analysis, with dodecane as internal standard.

It was found that by changing the solvent from THF to DCM, and the use of PI/CB-Au/Pt as a catalyst enabled the aerobic oxidation of benzyl alcohol 1i in an efficient manner (entry 5). Thus, a new chiral bifunctional heterogeneous catalyst IOC-PI/CB(Au/Pt) **11** was prepared and evaluated for the asymmetric TOP with benzyl alcohols substituted with electron-withdrawing moieties.

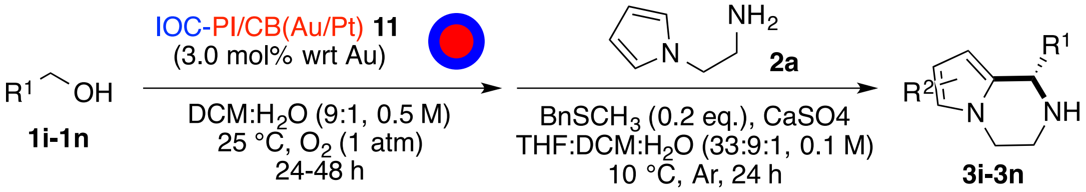

To a Carousel tube was charged (4-fluorophenyl)methanol **1i** (37.8 mg, 0.30 mmol), IOC-PI/CB(Au/Pt) **11** (92.3 mg, 0.006 mmol wrt Au), DCM (540 µL) and H_2O (60 µL). The carousel tube was flushed with O_2 gas and the reaction mixture was stirred at 25 ºC for 24 hours under a balloon pressure of O_2. Then, *N*-aminoethylpyrrole **2a** (22.0 mg, 0.2 mmol), $CaSO_4$ (200 mg), $BnSCH_3$ (5.6 mg, 0.04 mmol), and THF (2.0 mL) were added in sequence. The carousel tube was then flushed with Ar gas and the mixture was stirred at 10 ºC for an additional 24 hours. After completion of the asymmetric TOP, the heterogeneous catalyst **11** and $CaSO_4$ were separated via vacuum-filtration and subsequently washed with THF (20 mL). All solvents were removed under reduced pressure and the crude reaction mixture was purified by PTLC using EtOAc/hexane (1:1) as the eluent to provide chiral piperazine **3i** (39.8 mg, 92% yield, 91% ee) as a white solid.

4.3 Characterization Data

With the exception of compounds **3e-g**, **3l**, all chiral piperazines **3** (with the opposite enantiomer) are known compounds.[4]

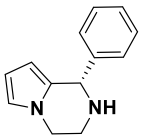

(*S*)-1-Phenyl-1,2,3,4-tetrahydropyrrolo[1,2-*a*]pyrazine (3a)

White solid, 89% yield and 94% ee, determined by chiral HPLC analysis (Chiralcel AD-H, hexane/isopropanol, 97:3 v/v, 0.6 mL/min, 25 °C, UV 254 nm): Retention times: t_r = 22.801 min for (*S*)-isomer (major), t_r = 25.777 min for (*R*)-isomer. **^{1}H NMR** (600 MHz, $CDCl_3$) δ 7.40-7.39 (m, 2H), 7.33 (t, *J* = 7.4 Hz, 2H), 7.30-7.29 (m, 1H), 6.59 (s, 1H), 6.11 (t, *J* = 2.8 Hz, 1H), 5.54 (d, *J* = 1.6 Hz, 1H), 5.08 (s, 1H), 4.10-4.05 (m, 1H), 4.01-3.96 (m, 1H), 3.37-3.33 (m, 1H), 3.28-3.23 (m, 1H), 1.86 (bs, 1H). **^{13}C NMR** (150 MHz, $CDCl_3$) δ 142.9, 130.6, 128.3, 127.7, 118.9, 107.6, 104.8, 59.0, 45.4, 43.1.

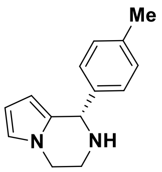

(*S*)-1-(p-Tolyl)-1,2,3,4-tetrahydropyrrolo[1,2-*a*]pyrazine (3b)

White solid, 89% yield and 84% ee, determined by chiral HPLC analysis (Chiralcel AD-H, hexane/isopropanol, 98:2 v/v, 0.6 mL/min, 25 °C, UV 250 nm): Retention times: t_r = 30.777 min for (*S*)-isomer (major), t_r = 37.009 min for (*R*)-isomer. **^{1}H NMR** (600 MHz, $CDCl_3$) δ 7.28 (d, *J* = 7.8 Hz, 2H), 7.15 (d, *J* = 7.7 Hz, 2H), 6.59 (s, 1H), 6.10 (t, *J* = 3.0 Hz, 1H), 5.55 (d, *J* = 1.7 Hz, 1H), 5.05 (s, 1H), 4.10-4.06 (m, 1H), 4.02-3.97 (m, 1H), 3.38-3.34 (m, 1H), 3.28-3.24 (m, 1H), 2.35 (s, 3H), 1.75 (bs, 1H). **^{13}C NMR** (150 MHz, $CDCl_3$) δ 139.9, 137.4, 130.9, 129.0, 128.2, 118.9, 107.6, 104.8, 58.8, 45.4, 43.1, 21.1.

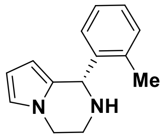

(*S*)-1-(o-Tolyl)-1,2,3,4-tetrahydropyrrolo[1,2-*a*]pyrazine (3c)

White solid, m.p. = 74 ºC 91% yield and 92% ee, $[\alpha]_D^{20}$= -35.6 (c= 0.1, $CHCl_3$) determined by chiral HPLC analysis (Chiralcel AD-H, hexane/isopropanol, 98:2 v/v,

0.6 mL/min, 25 °C, UV 250 nm): Retention times: t_r = 22.725 min for (*S*)-isomer (major), t_r = 23.939 min for (*R*)-isomer. **^{1}H NMR** (600 MHz, $CDCl_3$) δ 7.32 (d, *J* = 7.6 Hz, 1H), 7.21-7.20 (m, 2H), 7.18-7.15 (m, 1H), 6.62 (s, 1H), 6.13 (t, *J* = 3.1 Hz, 1H), 5.53-5.52 (m, 1H), 5.33 (s, 1H), 4.13-4.07 (m, 1H), 4.03-3.99 (m, 1H), 3.41-3.38 (m, 1H), 3.31-3.26 (m, 1H), 2.43 (s, 3H), 1.82 (bs, 1H). **^{13}C NMR** (150 MHz, $CDCl_3$) δ 140.8, 136.3, 130.6, 130.4, 128.5, 127.4, 125.9, 118.8, 107.6, 104.3, 55.9, 45.5, 43.3, 19.3. HRMS (ESI) calcd for $C_{14}H_{17}N_2$ $[M+H]^+$:213.1386; found 213.1389. IR (KBr) ν = 748, 764, 1073, 1293, 1488, 2360, 2953 cm^{-1}.

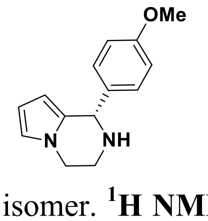

(*S*)-1-(4-Methoxyphenyl)-1,2,3,4-tetrahydropyrrolo[1,2-*a*]pyrazine (3d)

White solid, 80% yield and 80% ee, determined by chiral HPLC analysis (Chiralcel AD-H, hexane/isopropanol, 80:20 v/v, 0.60 mL/min, 25 °C, UV 250 nm): Retention times: t_r = 13.530 min for (*S*)-isomer (major), t_r = 16.402 min for (*R*)-isomer. **^{1}H NMR** (600 MHz, $CDCl_3$) δ 7.31 (d, *J* = 8.7 Hz, 2H), 6.87 (d, *J* = 8.7 Hz, 2H), 6.59 (s, 1H), 6.11 (t, *J* = 2.6 Hz, 1H), 5.54 (d, *J* = 1.7 Hz, 1H), 5.03 (s, 1H), 4.10-4.05 (m, 1H), 3.99-3.97 (m, 1H), 3.81 (s, 3H), 3.38-3.34 (m, 1H), 3.28-3.23 (m, 1H), 1.68 (bs, 1H). **^{13}C NMR** (150 MHz, $CDCl_3$) δ 159.2, 135.1, 131.1, 129.4, 118.9, 113.7, 107.6, 104.8, 58.5, 55.3, 45.4, 43.2.

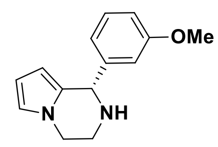

(*S*)-1-(3-Methoxyphenyl)-1,2,3,4-tetrahydropyrrolo[1,2-*a*]pyrazine (3e)

White solid, m.p. = 75 ºC, 83% yield and 93% ee, $[\alpha]_D^{20}$ = -72.5 (c = 0.1, $CHCl_3$) determined by chiral HPLC analysis (Chiralcel AD-H, hexane/isopropanol, 90:10 v/v, 1.0 mL/min, 25 °C, UV 202 nm): Retention times: t_r = 13.133 min for (*S*)-isomer (major), t_r = 18.890 min for (*R*)-isomer. **^{1}H NMR** (600 MHz, $CDCl_3$) δ 7.26-7.24 (m, 1H), 6.99-6.96 (m, 2H), 6.85-6.83 (m, 1H), 6.59 (s, 1H), 6.11 (t, *J* = 3.1 Hz, 1H), 5.59 (m, 1H), 5.07 (s, 1H), 4.09–4.05 (m, 1H), 4.00-3.96 (m, 1H), 3.79 (s, 3H), 3.38-3.35 (m, 1H), 3.28-3.25 (m, 1H), 1.79 (bs, 1H). **^{13}C NMR** (100 MHz, $CDCl_3$) δ 159.7, 144.4, 130.4, 129.3, 120.6, 118.9, 113.7, 113.3, 107.6, 104.9, 59.0, 55.2, 45.4, 43.1. HRMS (ESI) calcd for $C_{14}H_{15}N_2O$ $[M-H]^+$:227.1184; found 227.1188. IR (KBr) ν = 749, 1040, 1265, 1284, 1586, 2360, 2835, 2942 cm^{-1}.

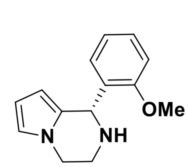

(*S*)-1-(2-Methoxyphenyl)-1,2,3,4-tetrahydropyrrolo[1,2-*a*]pyrazine (3f)

Light yellow solid, m.p. = 69 ºC, $[\alpha]_D^{20}$ = -97.5 (c= 0.1, $CHCl_3$) 85% yield and 91% ee, determined by chiral HPLC analysis (Chiralcel OD-H, hexane/isopropanol, 90:10 v/v, 0.60 mL/min, 25 °C, UV 250 nm): Retention times: t_r = 12.546 min for (*R*)-isomer, t_r = 18.642 min for (*S*)-isomer (major). **^{1}H NMR** (600 MHz, $CDCl_3$) δ 7.26-7.23 (m, 1H), 7.12 (d, *J* = 7.5 Hz, 1H), 6.92-6.86 (m, 2H), 6.60 (d, *J* = 1.4 Hz, 1H), 6.13 (t, *J* = 3.0 Hz, 1H), 5.63 (m, 1H), 5.55 (s, 1H), 4.04-3.98 (m, 2H), 3.85 (s, 3H), 3.28-3.25 (m, 1H), 3.17-3.14 (m, 1H), 1.99 (bs, 1H). **^{13}C NMR** (150 MHz, $CDCl_3$) δ 157.1, 131.2, 129.6, 129.4, 128.7, 120.4, 118.7, 110.5, 107.55, 104.5, 55.5, 52.3, 45.5, 41.8. HRMS (ESI) calcd for $C_{14}H_{17}N_2O$ $[M+H]^+$:229.1335; found 229.1336. IR (KBr) ν = 756, 841, 1027, 1102, 1242, 1293, 1490, 1599, 2835, 2952 cm^{-1}.

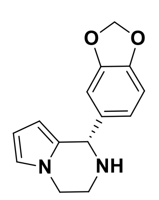

(*S*)-1-(Benzo[*d*][1,3]dioxol-5-yl)-1,2,3,4-tetrahydropyrrolo[1,2-*a*]pyrazine (3g)

White solid, m.p. = 66 ºC, $[\alpha]_D^{20}$ = -15.5 (c = 0.1, $CHCl_3$) 84% yield and 73% ee, determined by chiral HPLC analysis (Chiralcel AD-H, hexane/isopropanol, 98:2 v/v, 0.6 mL/min, 25 °C, UV 254 nm): Retention times: t_r = 60.189 min for (*R*)-isomer, t_r =

63.849 min for (*S*)-isomer (major). **^{1}H NMR** (500 MHz, $CDCl_3$) δ 6.89-6.86 (m, 2H), 6.77 (d, *J* = 7.9 Hz, 1H), 6.59 (dt, *J* = 2.3, 1.3 Hz, 1H), 6.12-6.10 (m, 1H), 5.94 (s, 2H), 5.58-5.57 (m, 1H), 5.01 (s, 1H), 4.10-4.04 (m, 1H), 3.99-3.95 (m, 1H), 3.38-3.33 (m, 1H), 3.28-3.22 (m, 1H). **^{13}C NMR** (125 MHz, $CDCl_3$) δ 147.6, 147.1, 137.0, 130.7, 121.6, 118.9, 108.7, 107.9, 107.6, 104.8, 101.0, 58.8, 45.4, 43.1. HRMS (ESI) calcd for $C_{14}H_{14}N_2O_2$ $[M+H]^+$: 243.1113, found 243.1114. IR(KBr) υ = 552, 710, 796, 944, 1100, 1176, 1360, 1485, 2878, 2922, 3431 cm^{-1}.

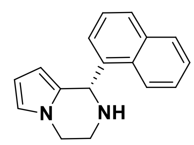

(*S*)-1-(Naphthalen-1-yl)-1,2,3,4-tetrahydropyrrolo[1,2-*a*]pyrazine (3h)

White solid, 85% yield and 93% ee, determined by chiral HPLC analysis (Chiralcel AD-H, hexane/isopropanol, 98:2 v/v, 0.6 mL/min, 25 °C, UV 250 nm): Retention times: t_r = 43.761 min for (*S*)-isomer (major), t_r = 50.844 min for (*R*)-isomer. **^{1}H NMR** (600 MHz, $CDCl_3$) δ 8.21 (d, *J* = 8.1 Hz, 1H), 7.88 (d, *J* = 7.6 Hz, 1H), 7.86 (d, *J* = 8.2 Hz, 1H), 7.50-7.46 (m, 3H), 7.39 (dd, *J* = 11.2, 4.0 Hz, 1H), 6.65 (s, 1H), 6.11 (t, *J* = 2.8 Hz, 1H), 5.87 (s, 1H), 5.55 (dd, *J* = 3.1, 1.2 Hz, 1H), 4.18-4.13 (m, 1H), 4.07-4.03 (m, 1H), 3.41-3.37 (m, 1H), 3.33-3.28 (m, 1H), 1.84 (bs, 1H). **^{13}C NMR** (150 MHz, $CDCl_3$) δ 138.4, 134.1, 131.5, 130.1, 128.8, 128.3, 126.1, 126.0, 125.5, 125.2, 124.0, 118.9, 107.2, 104.9, 55.7, 45.6, 43.0.

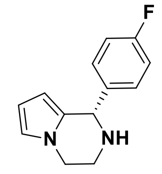

(*S*)-1-(4-Fluorophenyl)-1,2,3,4-tetrahydropyrrolo[1,2-*a*]pyrazine (3i)

White solid, 91% yield and 90% ee, determined by chiral HPLC analysis (Chiralpak OD-H, hexane/isopropanol, 95:5 v/v, 0.6 mL/min, 25 °C, UV 250 nm): Retention times: t_r = 17.957 min for (*S*)-isomer (major), t_r = 22.329 min for (*R*)-isomer. **^{1}H NMR** (500 MHz, $CDCl_3$) δ 7.38-7.36 (m, 2H), 7.01 (t, *J* = 8.6 Hz, 2H), 6.60 (s, 1H), 6.11 (t, *J* = 3.0 Hz, 1H), 5.53-5.52 (m, 1H), 5.07 (s, 1H), 4.10-4.06 (m, 1H), 4.00-3.97 (m, 1H), 3.37-3.34 (m, 1H), 3.29-3.24 (m, 1H), 1.75 (bs, 1H). **^{13}C NMR** (125 MHz, $CDCl_3$) δ 163.2, 161.5, 138.7, 130.5, 129.9, 129.9, 119.1, 115.2, 115.1, 107.7, 104.9, 58.4, 45.4, 43.1.

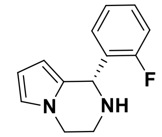

(*S*)-1-(2-Fluorophenyl)-1,2,3,4-tetrahydropyrrolo[1,2-*a*]pyrazine (3j)

White solid, 88% yield and 91% ee, determined by chiral HPLC analysis (Chiralcel AS-H, hexane/isopropanol, 98:2 v/v, 1.0 mL/min, 25 °C, UV 250 nm): Retention times: t_r = 15.920 min for (*S*)-isomer (major), t_r = 17.317 min for (*R*)-isomer. **^{1}H NMR** (500 MHz, $CDCl_3$) δ 7.29-7.25 (m, 2H), 7.09-7.06 (m, 2H), 6.60 (s, 1H), 6.13 (dd, 1H, *J*= 5.0, 5.0 Hz), 5.61 (m, 1H), 5.49 (s, 1H), 4.09-4.04 (m, 1H), 4.02-3.98 (m, 1H), 3.36-3.32 (m, 1H), 3.27-3.22 (m, 1H), 1.86 (bs, 1H). **^{13}C NMR** (125 MHz, $CDCl_3$) δ 161.8, 159.8, 130.0, 129.95, 129.9, 129.2, 129.1, 128.9, 124.0, 124.0, 119.0, 115.4, 115.3, 111.9, 107.7, 104.6, 51.9, 45.4, 42.6.

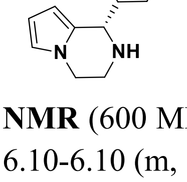

(*S*)-1-(4-chlorophenyl)-1,2,3,4-tetrahydropyrrolo[1,2-*a*]pyrazine (3k)

Colorless oil, 83% yield and 90% ee, determined by chiral HPLC analysis (Chiralcel OD-H, hexane/isopropanol, 80:20 v/v, 0.6 mL/min, 25 °C, UV 250 nm): Retention times: t_r = 11.151 min for (*S*)-isomer (major), t_r = 13.046 min for (*R*)-isomer. **^{1}H NMR** (600 MHz, $CDCl_3$) δ 7.34 (d, 2H, *J*= 12 Hz), 7.31 (d, 2H, *J*= 6 Hz), 6.60 (t, 1H, *J* = 4.1 Hz), 6.10-6.10 (m, 1H), 5.53-5.51 (m, 1H), 5.06 (s, 1H), 4.10-4.05 (m, 1H), 4.00-3.97 (m, 1H), 3.36-3.33 (m, 1H), 3.28-3.25 (m, 1H), 1.77 (bs, 1H). **^{13}C NMR** (150 MHz, $CDCl_3$) δ 141.4, 133.4, 130.1, 129.7, 128.5, 119.1, 107.7, 104.9, 58.4, 45.4, 43.0.

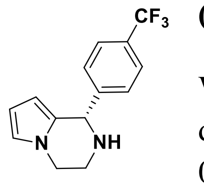

(*S*)-1-(4-(Trifluoromethyl)phenyl)-1,2,3,4-tetrahydropyrrolo[1,2-*a*]pyrazine (3l)

White solid, m.p. = 81 ºC , 82% yield and 86% ee, $[\alpha]_D^{20}$= -36.17 (c = 0.1, $CHCl_3$) determined by chiral HPLC analysis (Chiralcel AD-H, hexane/isopropanol, 99:1 v/v, 0.6 mL/min, 25 °C, UV 250 nm): Retention times: t_r = 36.690 min for (*S*)-isomer (major), t_r = 60.913 min for (*R*)-isomer. **^{1}H NMR** (600 MHz, $CDCl_3$) δ 7.59 (d, *J* = 8.1 Hz, 2H), 7.52 (d, *J* = 7.9 Hz, 2H), 6.61 (d, *J* = 1.8 Hz, 1H), 6.12-6.11 (m, 1H), 5.53 (m, 1H), 5.15 (s, 1H), 4.11-4.05 (m, 1H), 4.01-3.98 (m, 1H), 3.37-3.34 (m, 1H), 3.30-3.26 (m, 1H), 1.89 (bs, 1H). **^{13}C NMR** (150 MHz, $CDCl_3$) δ 146.9, 129.9, 129.6, 128.6, 125.4, 125.33, 123.27, 119.3, 107.8, 105.1, 58.5, 45.4, 42.9. HRMS (ESI) calcd for $C_{14}H_{12}F_3N_2$ $[M+H]^+$:265.0953; found 265.0957. IR (KBr) ν = 606, 710, 763, 1066, 1326, 1620, 2360, 2823, 3239 cm^{-1}.

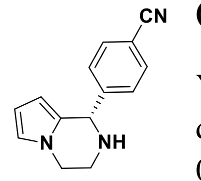

(*S*)-4-(1,2,3,4-Tetrahydropyrrolo[1,2-*a*]pyrazin-1-yl)benzonitrile (3m)

Yellow solid, m.p. = 78 ºC, 89% yield and 95% ee, $[\alpha]_D^{20}$ = -11.30 (c = 0.1, $CHCl_3$) determined by chiral HPLC analysis (Chiralcel AD-H, hexane/isopropanol, 98:2 v/v, 0.6 mL/min, 25 °C, UV 254 nm): Retention times: t_r = 20.570 min for (*R*)-isomer, t_r = 22.074 min for (*S*)-isomer (major). **^{1}H NMR** (400 MHz, $CDCl_3$) δ 7.63 (d, 2H, *J* = 8.2 Hz), 7.53 (d, 2H, *J* = 8.2 Hz), 6.62 (s, 1H), 6.12 (t, *J* = 3.1 Hz, 1H), 5.52-5.51 (m, 1H), 5.15 (s, 1H), 4.12-4.15 (m, 1H), 4.02-3.97 (m, 1H), 3.36-3.24 (m, 2H), 1.89 (bs, 1H). **^{13}C NMR** (100 MHz, $CDCl_3$) δ 142.9, 130.7, 128.4, 128.3, 127.7, 118.9, 107.6, 104.8, 59.1, 45.4, 43.1. HRMS (ESI) calcd for $C_{14}H_{14}N_2$ $[M+H]^+$: 224.1192, found 224.1188. IR(KBr) υ = 639, 680, 728, 944, 1163, 1368, 1465, 2361, 2874, 3063, 3228 cm^{-1}.

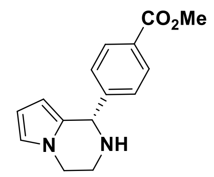

(*S*)-Methyl 4-(1,2,3,4-tetrahydropyrrolo[1,2-*a*]pyrazin-1-yl)benzoate (3n)

White solid, m.p. = 89 °C, 84% yield and 90% ee, $[\alpha]_D^{20}$ = -3.91 (c = 0.1, $CHCl_3$) determined by chiral HPLC analysis (Chiralcel AD-H, hexane/isopropanol, 90:10 v/v, 0.60 mL/min, 25 °C, UV 250 nm): Retention times: t_r = 21.623 min for (*R*)-isomer, t_r = 28.753 min for (*S*)-isomer (major). **^{1}H NMR** (600 MHz, $CDCl_3$) δ 8.01 (d, *J* = 8.3 Hz, 2H), 7.47 (d, *J* = 8.1 Hz, 2H), 6.61 (s, 1H), 6.11 (t, *J* = 3.1 Hz, 1H), 5.53 (m, 1H), 5.2 (s, 1H), 4.11-4.07 (m, 1H), 4.03-3.98 (m, 1H), 3.91 (s, 3H), 3.38-3.34 (m, 1H), 3.29-3.25 (m, 1H), 1.83 (bs, 1H). **^{13}C NMR** (150 MHz, $CDCl_3$) δ 166.9, 147.9, 129.7, 129.6, 128.3, 119.2, 107.8, 105.0, 58.6, 52.1, 45.3, 43.0. HRMS (ESI) calcd for $C_{15}H_{17}N_2O_2$ $[M+H]^+$: 257.1285; found 257.1279. IR (KBr) ν = 738, 767, 1019, 1115, 1280, 1436, 1610, 1720, 2810, 2952 cm^{-1}.

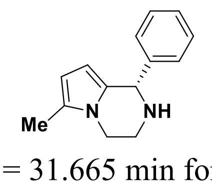

(*S*)-6-Methyl-1-phenyl-1,2,3,4-tetrahydropyrrolo[1,2-*a*]pyrazine (3o)

91% yield and 85% ee, determined by chiral HPLC analysis (Chiralcel AD-H, hexane/isopropanol, 97:3 v/v, 0.6 mL/min, 25 °C, UV 254 nm): Retention times: t_r = 31.665 min for (*R*)-isomer, t_r = 41.309 min for (*S*)-isomer (major). **^{1}H NMR** (600 MHz, $CDCl_3$) δ 7.40 (d, *J* = 7.2 Hz, 2H), 7.34-7.29 (m, 3H), 5.81 (d, *J* = 2.4 Hz, 1H), 5.43 (d, *J* = 2.8 Hz, 1H), 5.05 (s, 1H), 3.84-3.82 (m, 2H), 3.40-3.38 (m, 1H), 3.28-3.25 (m, 1H), 2.21 (s, 3H), 1.88 (bs, 1H). **^{13}C NMR** (125 MHz, $CDCl_3$) δ 142.9, 129.9, 128.3, 127.6, 126.9, 105.3, 104.0, 59.3, 43.1, 42.8, 11.5.

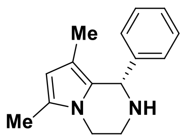

(*S*)-6,8-Dimethyl-1-phenyl-1,2,3,4-tetrahydropyrrolo[1,2-*a*]pyrazine (3p)

83% yield and 70% ee, determined by chiral HPLC analysis (Chiralpak AD-H, hexane/isopropanol, 97:3 v/v, 0.6 mL/min, 25 °C, UV 254 nm): Retention times: t_r = 26.174 min for (*R*)-isomer, t_r = 35.222 min for (*S*)-isomer (major). **^{1}H NMR** (400 MHz, $CDCl_3$) δ 7.31-7.26 (m, 5H), 5.72 (s, 1H), 5.12 (s, 1H), 3.83-3.72 (m, 2H), 3.22-3.16 (m, 1H), 3.14-3.08 (m, 1H), 2.20 (s, 3H), 1.55 (s, 3H). **^{13}C NMR** (100 MHz, $CDCl_3$) δ 143.1, 128.3, 127.3, 126.1, 123.6, 113.1, 107.4, 56.9, 42.8, 40.7, 11.4, 11.3.

4.4 Recovery and Reuse Studies

The recovery and reuse studies were performed for the tandem aerobic oxidation-asymmetric *aza*-FC reaction between **1a** and **2a** (Table 9S) using the following procedure.

At the end of the reaction, the solution/suspension was carefully vacuum filtered with 8 mm filter paper (made for the Kiriyama funnel) to separate the insoluble materials from the organic compounds. The collected solids were then washed several times with THF and DCM. Next, the separated solids were placed in 300 mL of water and sonicated for 20 minutes (**to dissolve $CaSO_4$**). This treated solid was filtered and washed three times with water, THF, and DCM. The solid material was then treated with aqueous H_2O_2 in MeOH and stirred overnight (**to oxidize $BnSCH_3$**). The resulting solid was then filtered and washed several time with water, THF, and DCM, and the recovered catalyst was heated at 170 °C for 5 h under an atmosphere of H_2. Finally, the recovered catalyst was transferred to a new Carousel tube for the next run.

Table 9S. Recovery and Reuse of IOC-PI/CB(Au/Pd) **8**

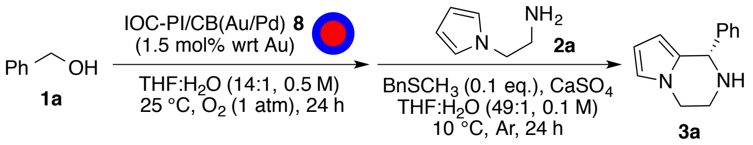

Run	Yield **3a** (%)[a]	Ee **3a** (%)[b]	Leaching (**Au**/**Pd**/**P**)[c]	Detection Limit (**Au**/**Pd**/**P**)[c]
1	92	89	ND/0.49%/ND	0.006/0.013/0.026 ppm
2	91	88	ND/0.24%/ND	
3	90	88	ND/0.19%/ND	
4	87	87	ND/0.21%/ND	0.01/0.014/0.015 ppm
5	88	88	ND/0.35%/ND	
6	85	84	ND/0.78%/ND	0.0075/0.011/0.032 ppm

[a] Yield was determined by isolation of 50% v/v of a crude sample. [b] The ee values were determined by chiral HPLC analysis. [c] Leaching was determined by ICP analysis from 50% v/v of a crude sample. ND = below the detection limit of the ICP equipment.

It should be noted that the scale of the reaction was adjusted according to the amount of the IOC-PI/CB(Au/Pd) **8** recovered from each run.

The Au/Pd/P leaching during the recovery and reuse studies was determined using the following procedure.

The filtrate obtained from each run was concentrated and diluted with 10 mL of THF. Then 50% v/v of the crude THF solution (5 mL) was then passed through a membrane filter (0.25 or 0.45 μm) into a new test tube. The solvent was then removed under reduced pressure. The solid obtained in the test tube was then heated to 200 °C and 1.0 mL of concentrated H_2SO_4 was added. After further heating for 15 min, drops of concentrated HNO_3 were added at regular intervals (45~60 min) until the resulting solution was lightly coloured (yellow or orange, but not brown) and clear. Brown fumes should not be observed at this point when additional HNO_3 is added. If brown fumes are still observed, the addition of HNO_3 at regular intervals should be continued. To the obtained cooled clear solution, 1.0 mL of aqua regia was added and the resulting mixture was shaken lightly at first and then more vigorously. It was then diluted to 50 mL with water and the resulting diluted solution was filtered into a vial to remove any remaining solids. The clear solution was then subjected to ICP analysis.

5. Copies of the ^{1}H and ^{13}C NMR Spectra

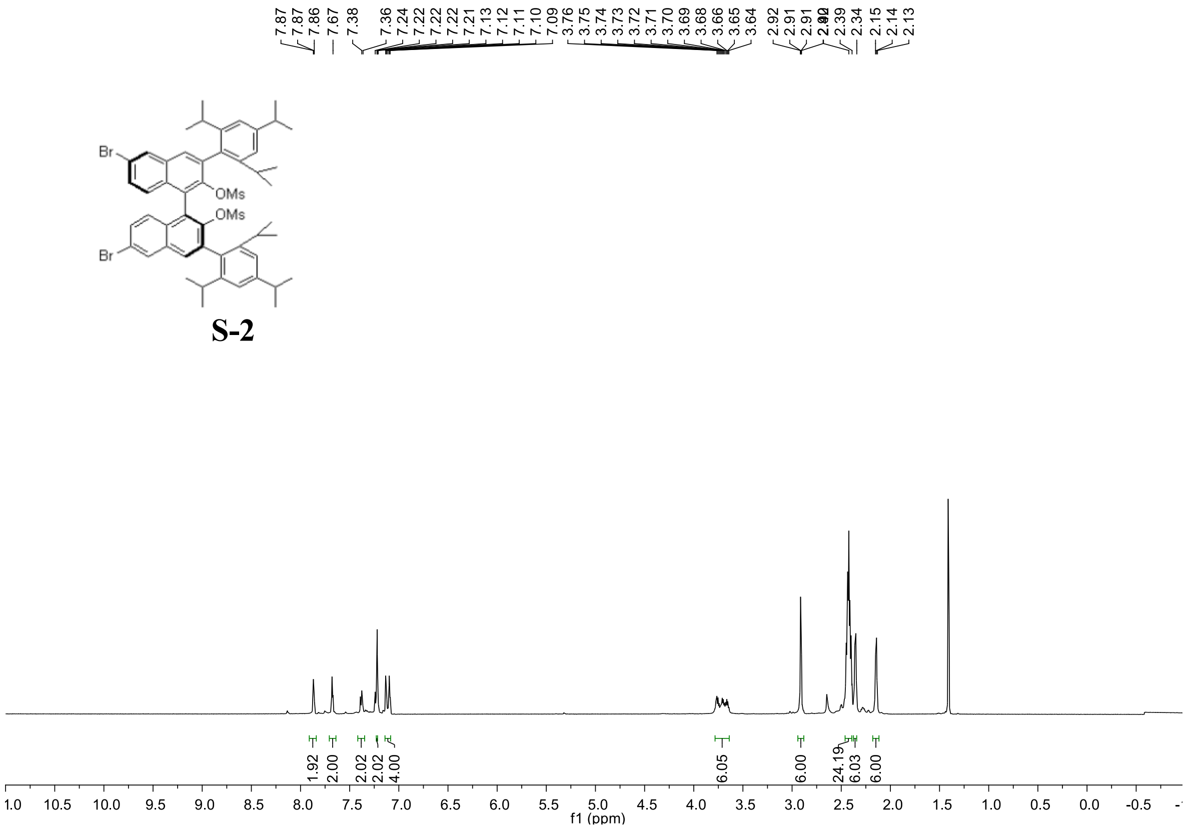

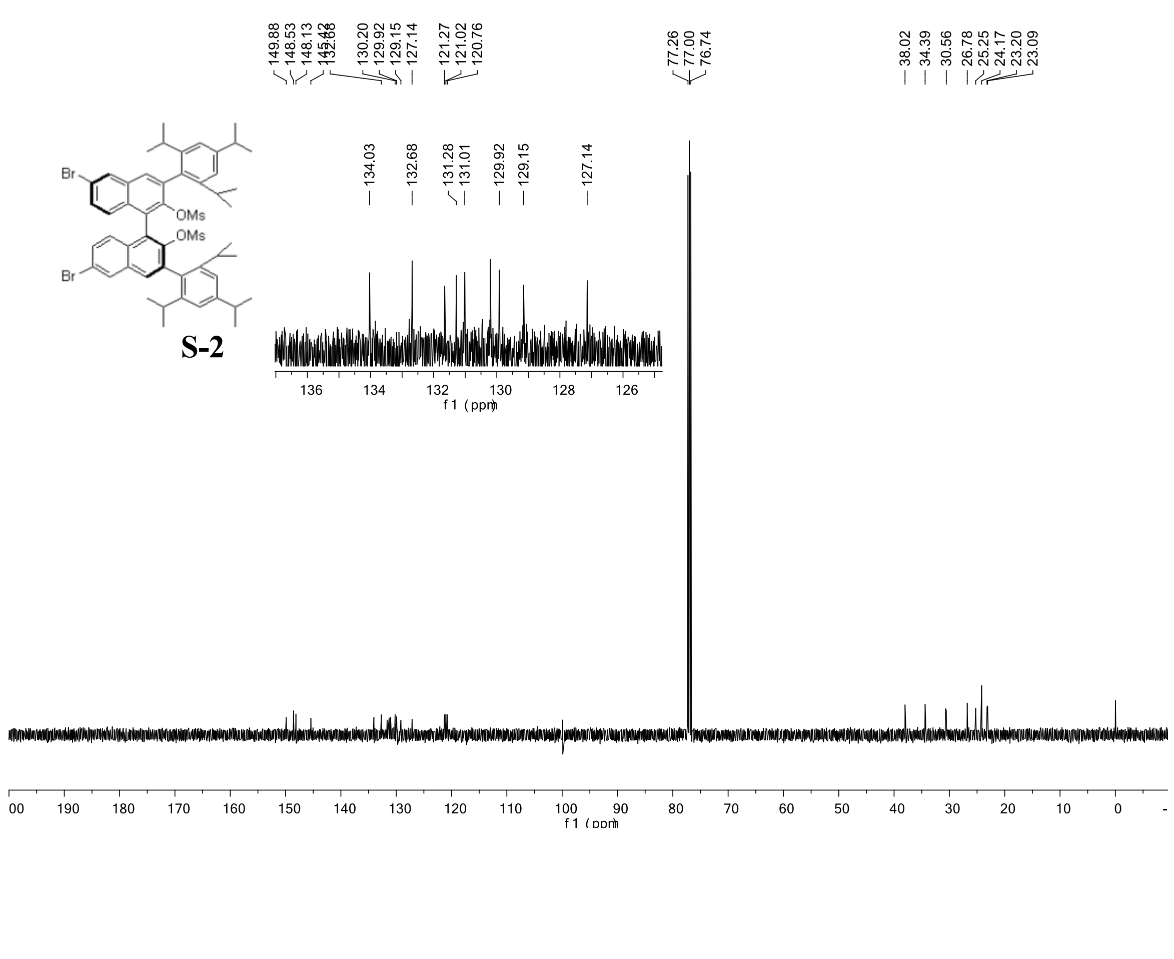

CHG372, CDCl3

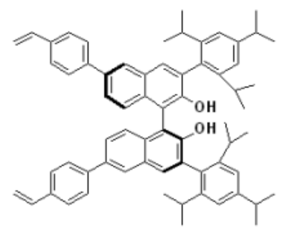

S-3

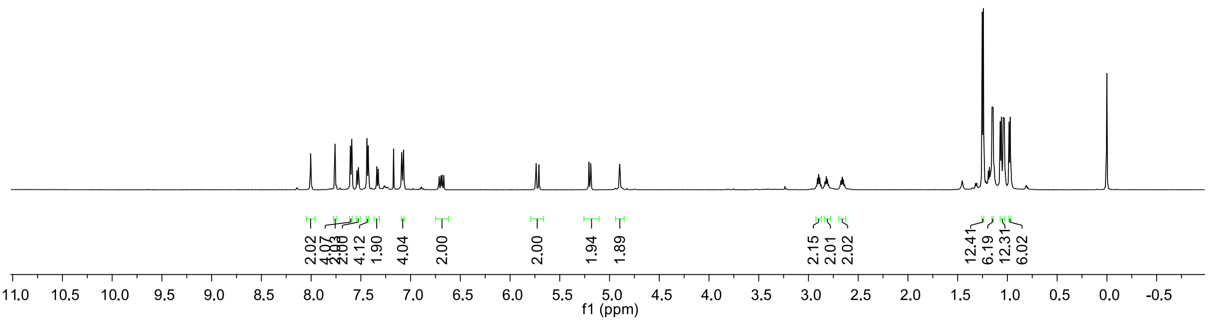

CHG372 CDC3

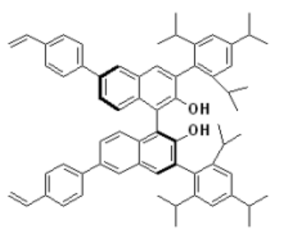

S-3

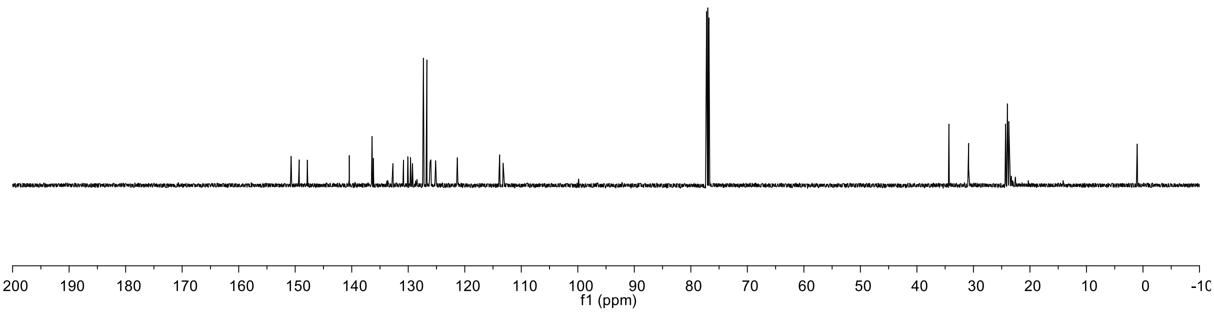

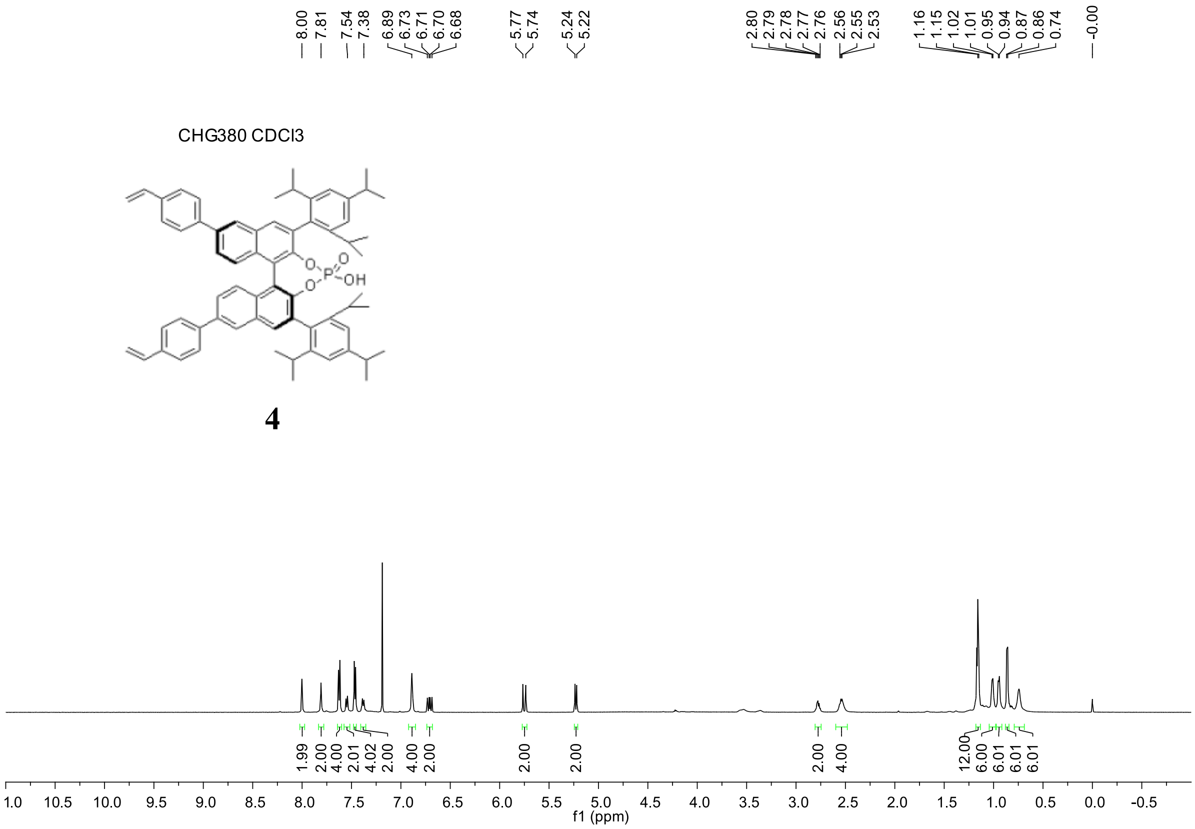
8.00
7.81
7.54
7.38
6.89
6.73
6.71
6.70
6.68
5.77
5.74
5.24
5.22
2.80
2.79
2.78
2.77
2.76
2.56
2.55
2.53
1.16
1.15
1.02
1.01
0.95
0.94
0.87
0.86
0.74
-0.00
CHG380 CDCl3
4
1.99
2.00
4.00
2.01
4.02
2.00
4.00
2.00
2.00
2.00
2.00
4.00
12.00
6.00
6.01
6.01
6.01
f1 (ppm)

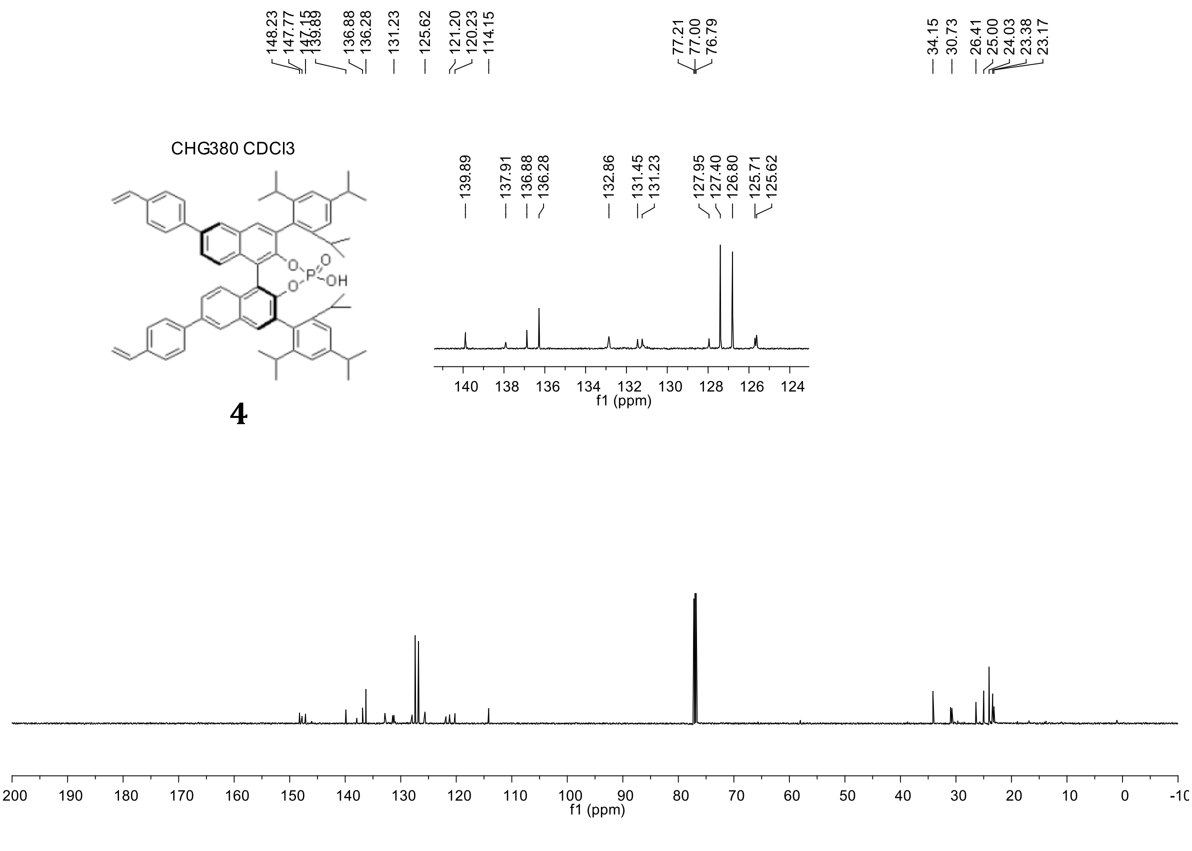
148.23
147.77
147.15
139.89
136.88
136.28
131.23
125.62
121.20
120.23
114.15
77.21
77.00
76.79
34.15
30.73
26.41
25.00
24.03
23.38
23.17
CHG380 CDCl3
4
139.89
137.91
136.88
136.28
132.86
131.45
131.23
127.95
127.40
126.80
125.71
125.62
f1 (ppm)

CHG380 CDCl3

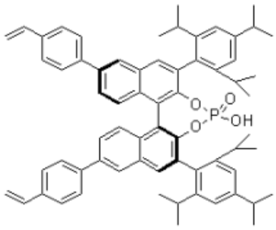

4

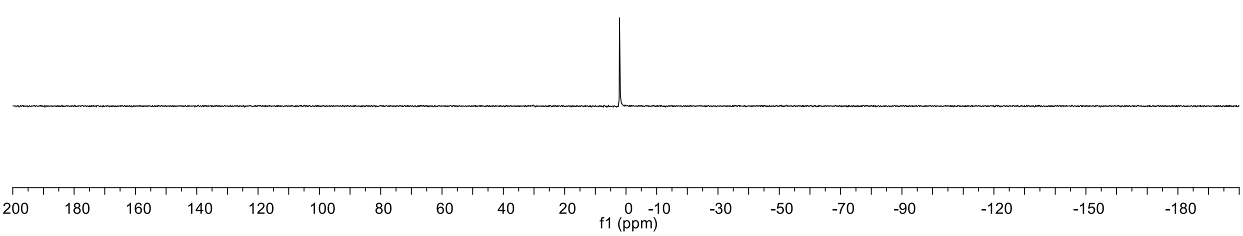

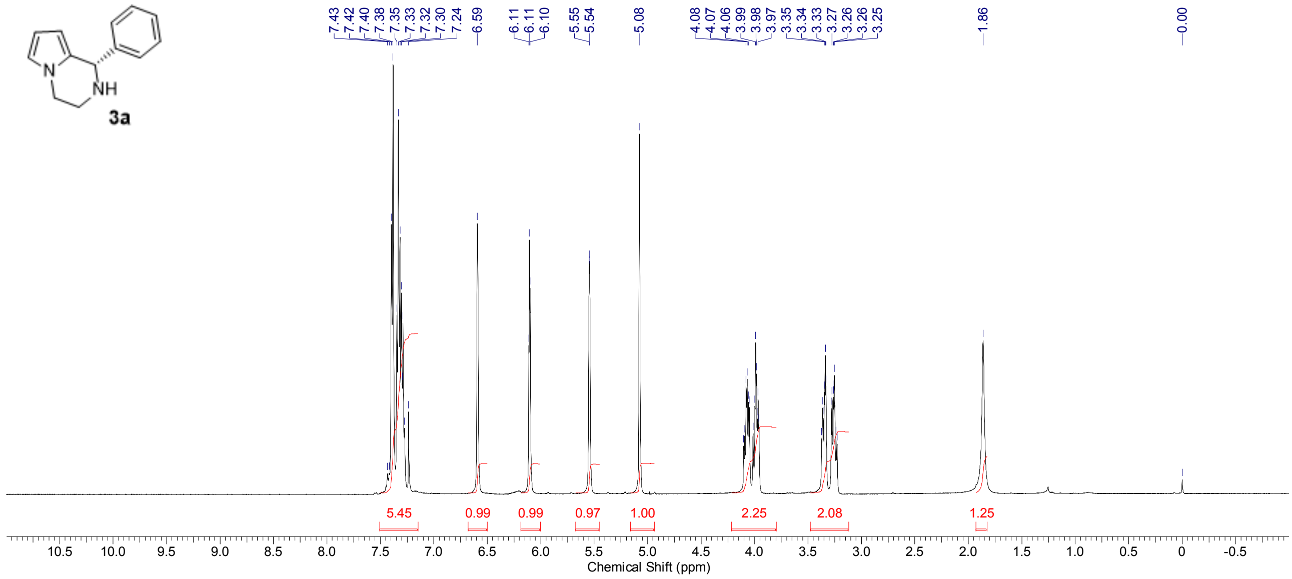

N
NH
3a
7.43
7.42
7.40
7.38
7.35
7.33
7.32
7.30
7.24
6.59
6.11
6.11
6.10
5.55
5.54
5.08
4.08
4.07
4.06
3.99
3.98
3.97
3.35
3.34
3.33
3.27
3.26
3.26
3.25
1.86
0.00
5.45
0.99
0.99
0.97
1.00
2.25
2.08
1.25
10.5 10.0 9.5 9.0 8.5 8.0 7.5 7.0 6.5 6.0 5.5 5.0 4.5 4.0 3.5 3.0 2.5 2.0 1.5 1.0 0.5 0 -0.5
Chemical Shift (ppm)

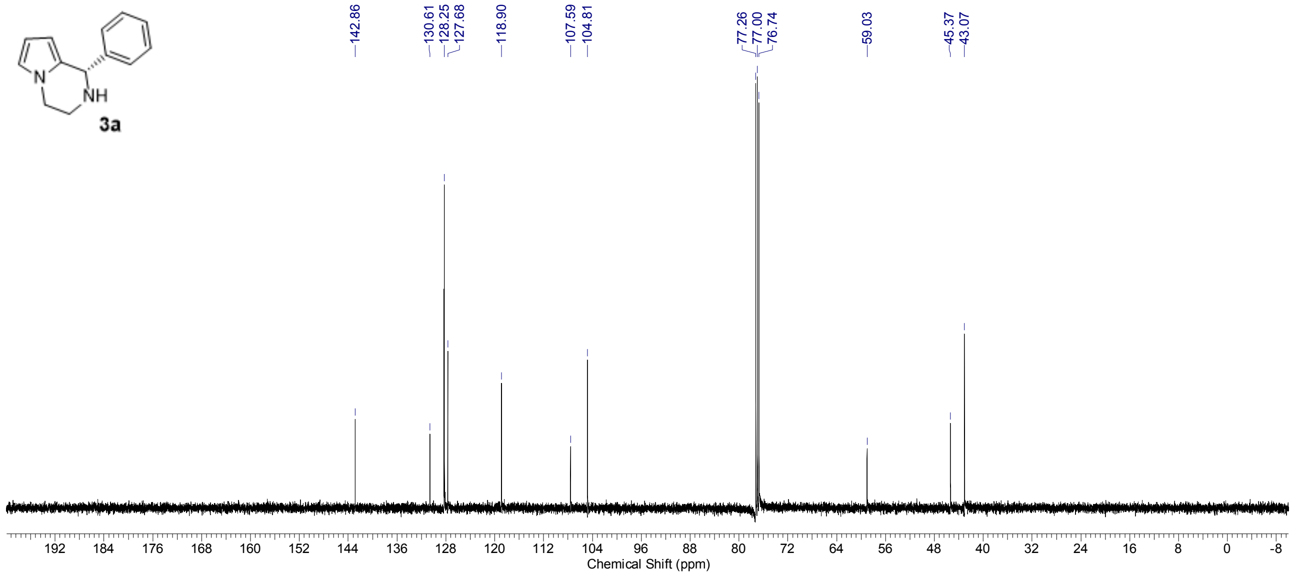

N
NH
3a
142.86
130.61
128.25
127.68
118.90
107.59
104.81
77.26
77.00
76.74
59.03
45.37
43.07
192 184 176 168 160 152 144 136 128 120 112 104 96 88 80 72 64 56 48 40 32 24 16 8 0 -8
Chemical Shift (ppm)

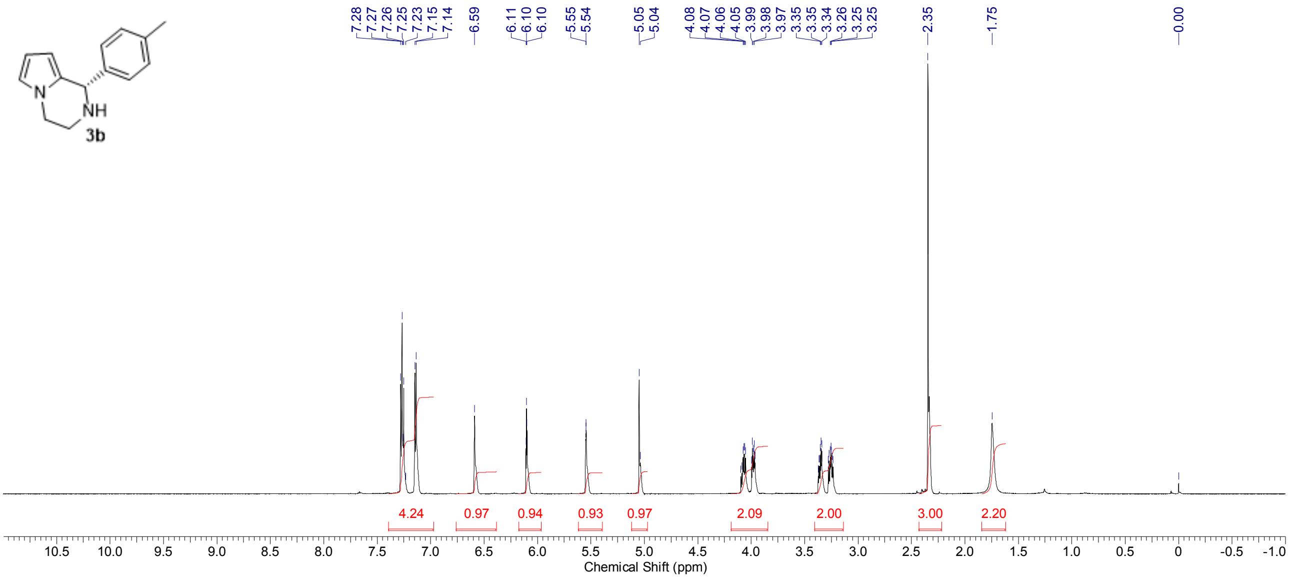
7.28
7.27
7.26
7.25
7.23
7.15
7.14
6.59
6.11
6.10
6.10
5.55
5.54
5.05
5.04
4.08
4.07
4.06
4.05
3.99
3.98
3.97
3.35
3.35
3.34
3.26
3.25
3.25
2.35
1.75
0.00
4.24
0.97
0.94
0.93
0.97
2.09
2.00
3.00
2.20
Chemical Shift (ppm)
NH
N
3b

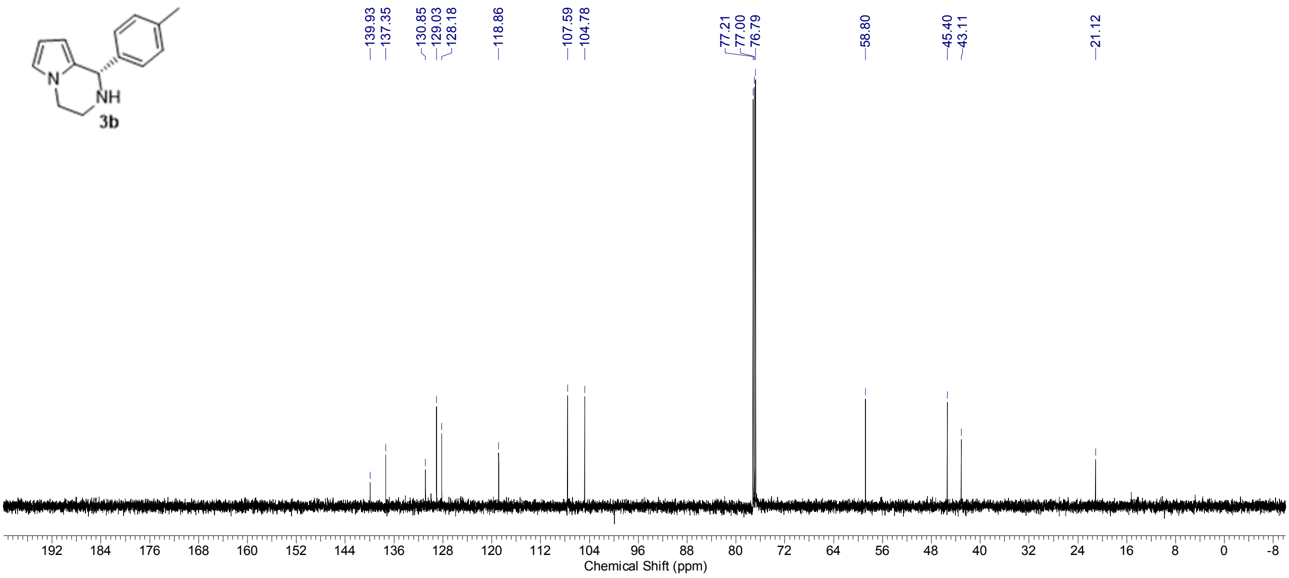
139.93
137.35
130.85
129.03
128.18
118.86
107.59
104.78
77.21
77.00
76.79
58.80
45.40
43.11
21.12
Chemical Shift (ppm)
NH
N
3b

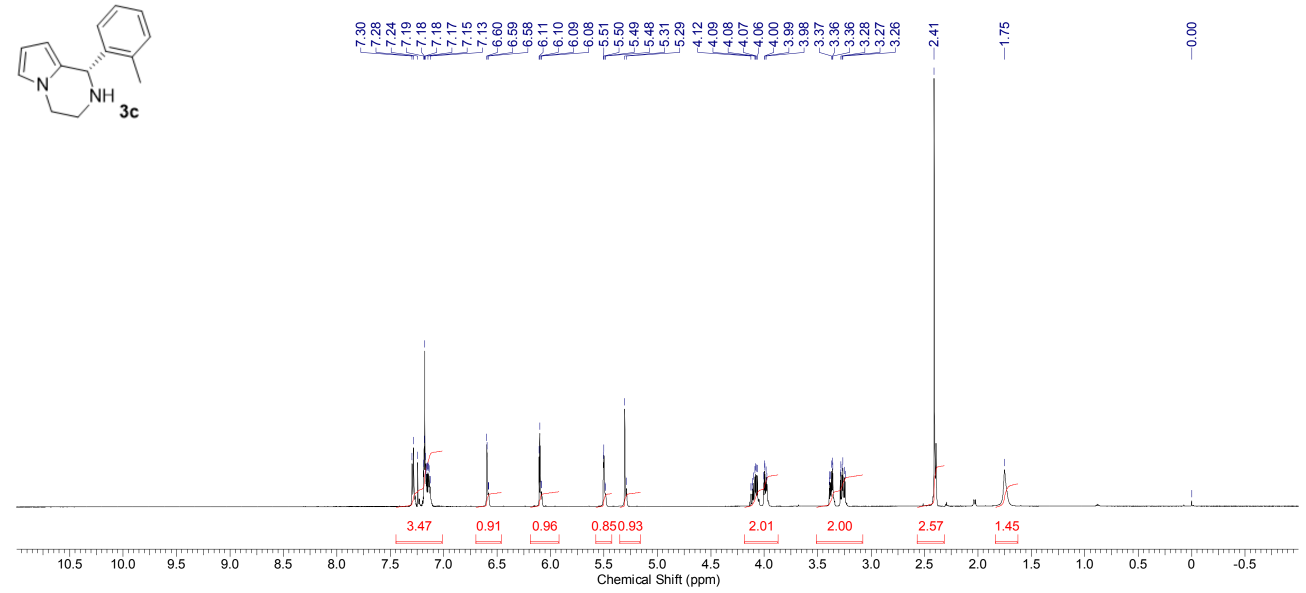

NH
N
3c
7.30 7.28 7.24 7.19 7.18 7.17 7.15 7.13 6.60 6.59 6.58 6.11 6.10 6.09 6.08 5.51 5.50 5.49 5.48 5.31 5.29 4.12 4.09 4.08 4.07 4.06 4.00 3.99 3.98 3.37 3.36 3.36 3.28 3.27 3.26 2.41 1.75 0.00
3.47 0.91 0.96 0.85 0.93 2.01 2.00 2.57 1.45
Chemical Shift (ppm)

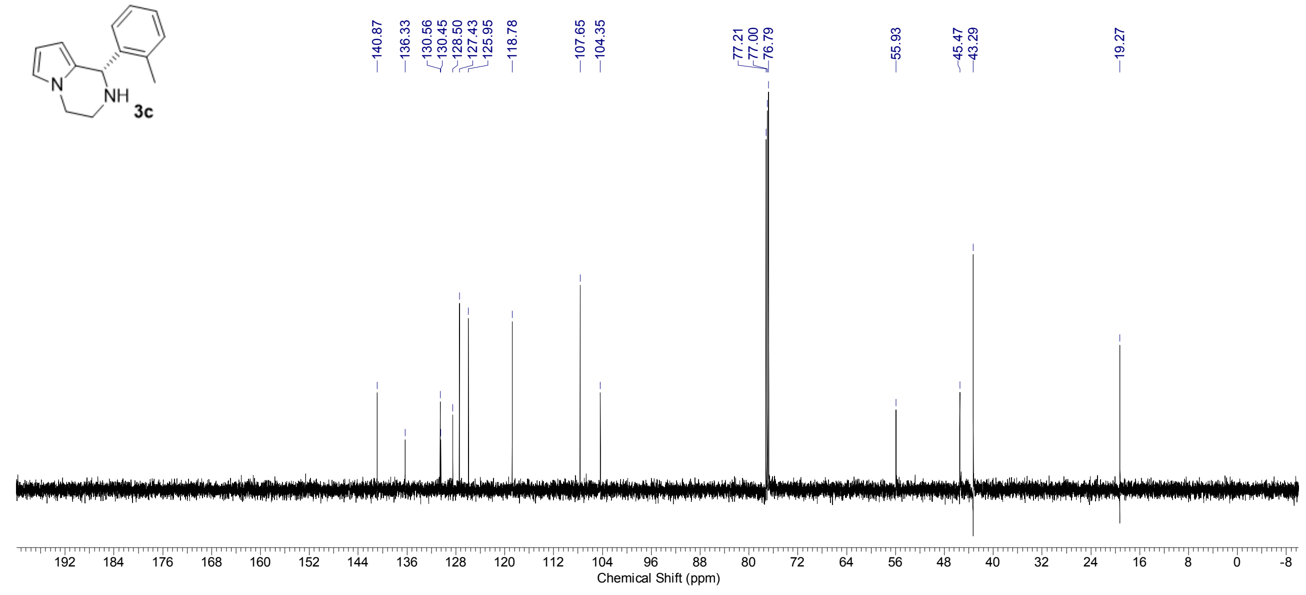

NH
N
3c
140.87 136.33 130.56 130.45 128.50 127.43 125.95 118.78 107.65 104.35 77.21 77.00 76.79 55.93 45.47 43.29 19.27
Chemical Shift (ppm)

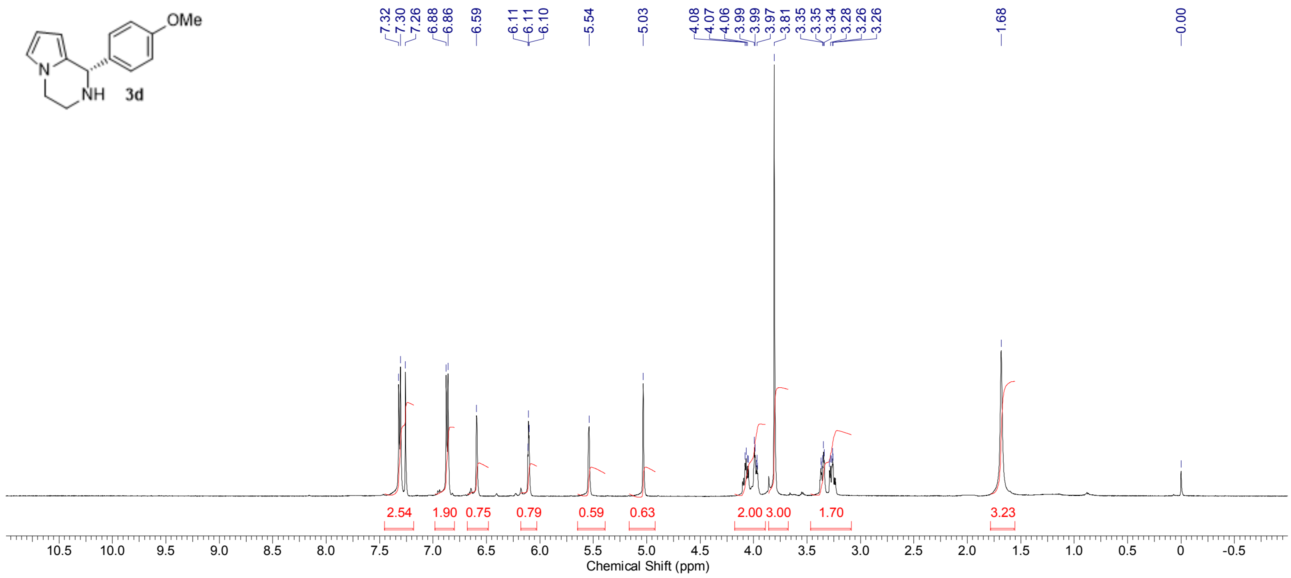
OMe
N
NH
3d
7.32
7.30
7.26
6.88
6.86
6.59
6.11
6.11
6.10
5.54
5.03
4.08
4.07
4.06
3.99
3.99
3.97
3.81
3.35
3.35
3.34
3.28
3.26
3.26
1.68
0.00
2.54 1.90 0.75 0.79 0.59 0.63 2.00 3.00 1.70 3.23
10.5 10.0 9.5 9.0 8.5 8.0 7.5 7.0 6.5 6.0 5.5 5.0 4.5 4.0 3.5 3.0 2.5 2.0 1.5 1.0 0.5 0 -0.5
Chemical Shift (ppm)

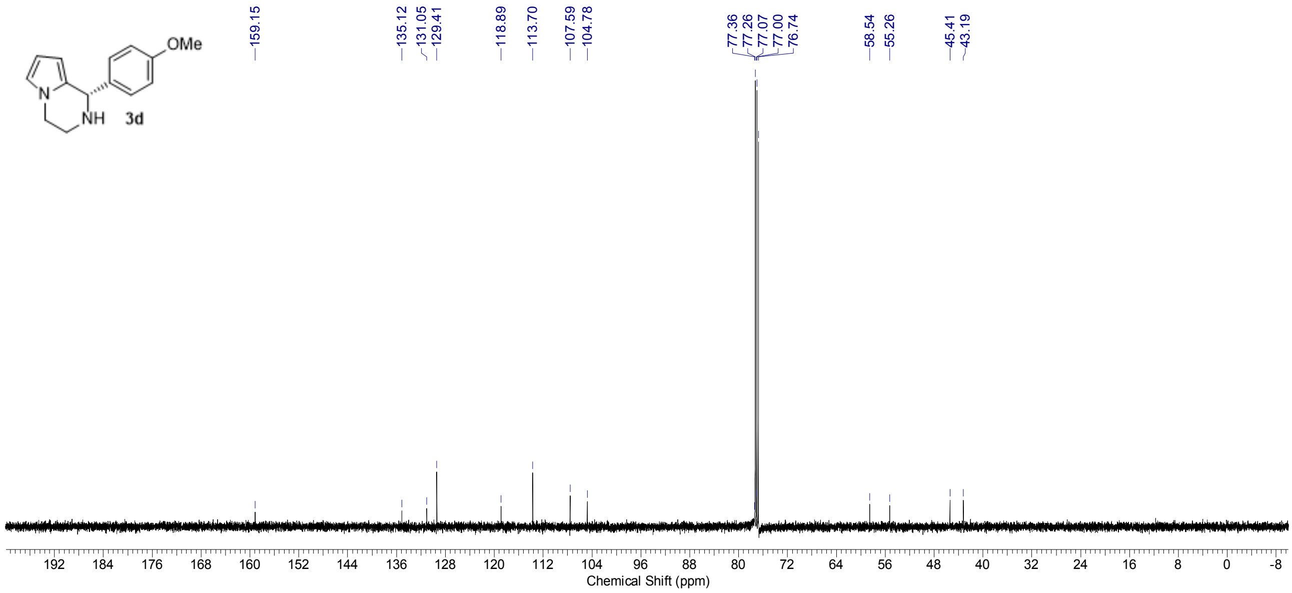
OMe
N
NH
3d
159.15
135.12
131.05
129.41
118.89
113.70
107.59
104.78
77.36
77.26
77.07
77.00
76.74
58.54
55.26
45.41
43.19
192 184 176 168 160 152 144 136 128 120 112 104 96 88 80 72 64 56 48 40 32 24 16 8 0 -8
Chemical Shift (ppm)

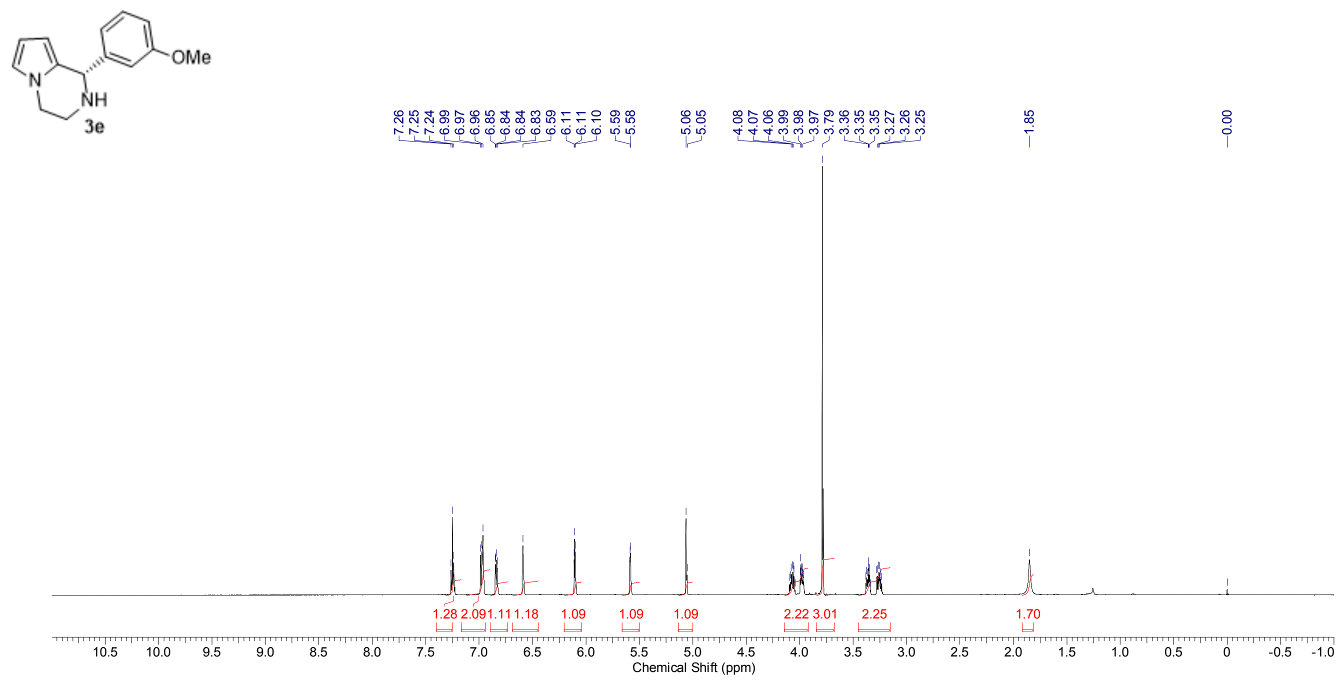
OMe
N
NH
3e
7.26 7.25 7.24 6.99 6.97 6.96 6.85 6.84 6.84 6.83 6.59 6.11 6.11 6.10 5.59 5.58 5.06 5.05 4.08 4.07 4.06 3.99 3.98 3.97 3.79 3.36 3.35 3.35 3.27 3.26 3.25 1.85 0.00
1.28 2.09 1.11 1.18 1.09 1.09 1.09 2.22 3.01 2.25 1.70
Chemical Shift (ppm)

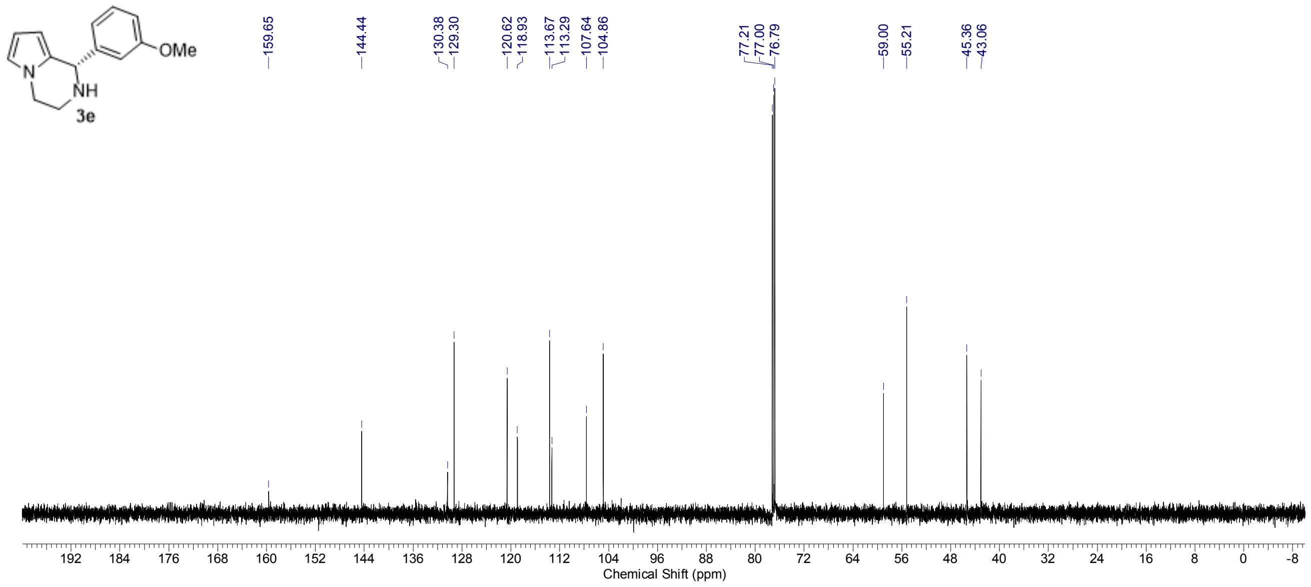
OMe
N
NH
3e
159.65 144.44 130.38 129.30 120.62 118.93 113.67 113.29 107.64 104.86 77.21 77.00 76.79 59.00 55.21 45.36 43.06
Chemical Shift (ppm)

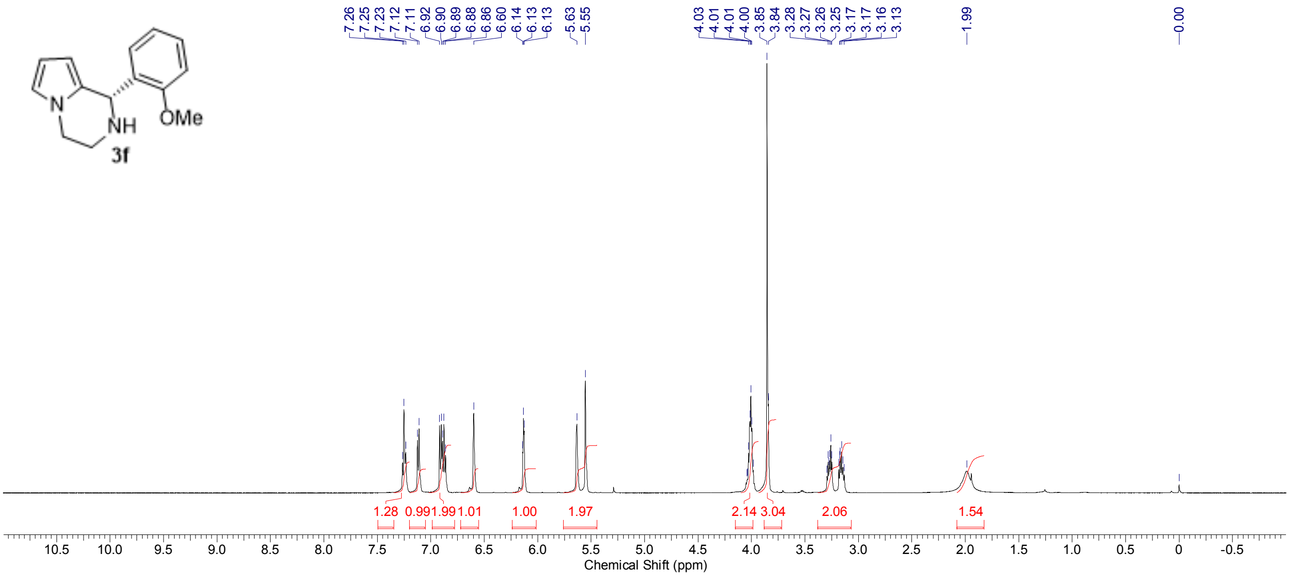
7.26
7.25
7.23
7.12
7.11
6.92
6.90
6.89
6.88
6.86
6.60
6.14
6.13
6.13
5.63
5.55
4.03
4.01
4.01
4.00
3.85
3.84
3.28
3.27
3.26
3.25
3.17
3.16
3.13
1.99
0.00
N
NH
OMe
3f
1.28
0.99
1.99
1.01
1.00
1.97
2.14
3.04
2.06
1.54
Chemical Shift (ppm)

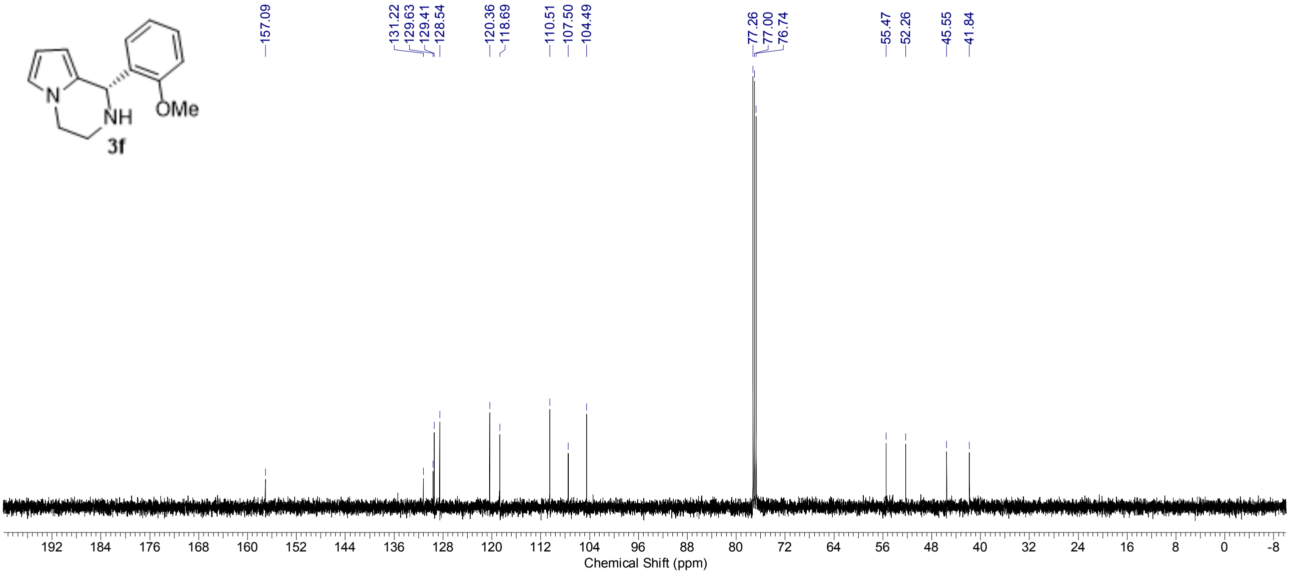
157.09
131.22
129.63
129.41
128.54
120.36
118.69
110.51
107.50
104.49
77.26
77.00
76.74
55.47
52.26
45.55
41.84
N
NH
OMe
3f
Chemical Shift (ppm)

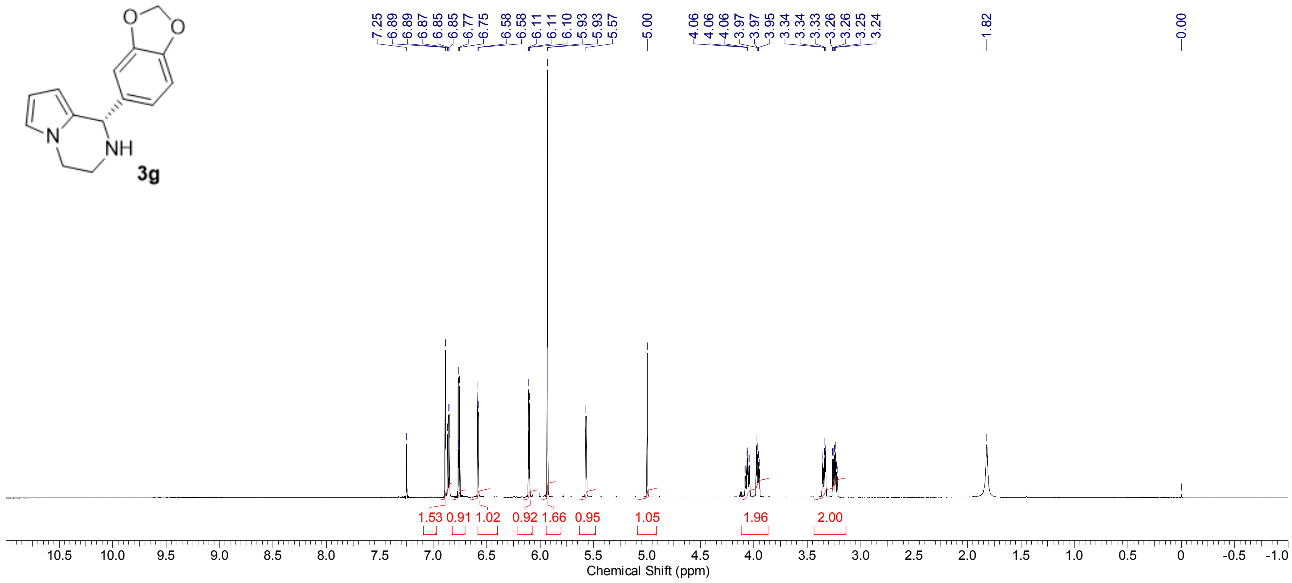

3g
7.25
6.89
6.89
6.87
6.85
6.85
6.77
6.75
6.58
6.58
6.11
6.11
6.10
5.93
5.93
5.57
5.00
4.06
4.06
4.06
3.97
3.97
3.95
3.34
3.34
3.33
3.26
3.26
3.25
3.24
1.82
0.00
1.53 0.91 1.02 0.92 1.66 0.95 1.05 1.96 2.00
Chemical Shift (ppm)

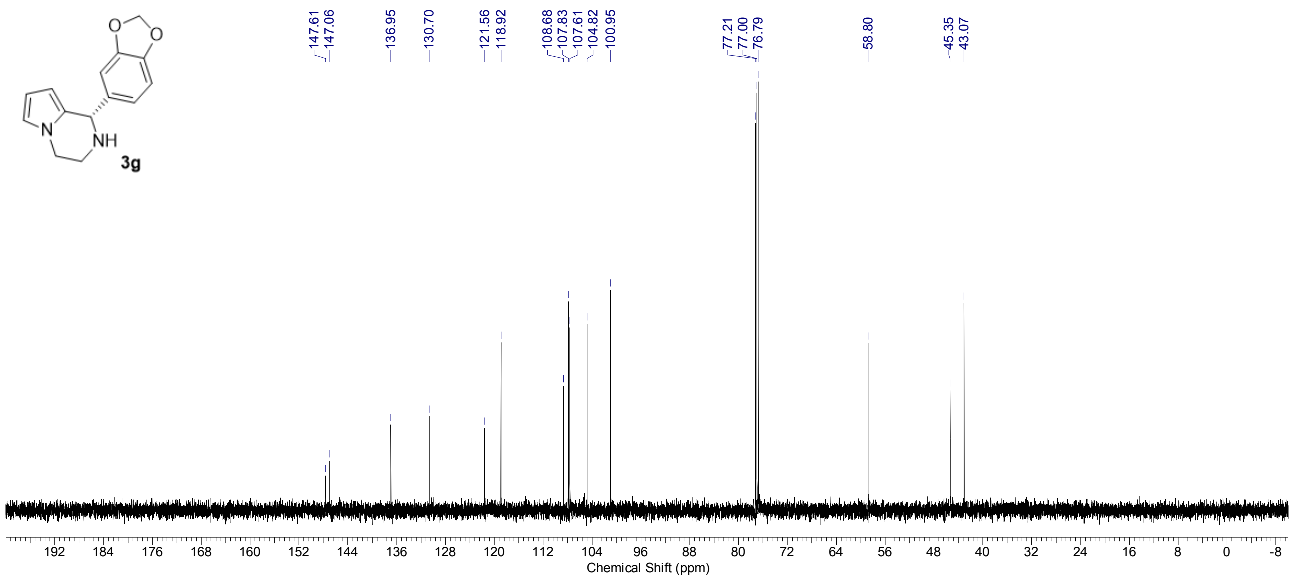

3g
147.61
147.06
136.95
130.70
121.56
118.92
108.68
107.83
107.61
104.82
100.95
77.21
77.00
76.79
58.80
45.35
43.07
Chemical Shift (ppm)

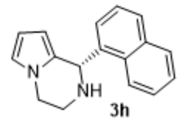

3h

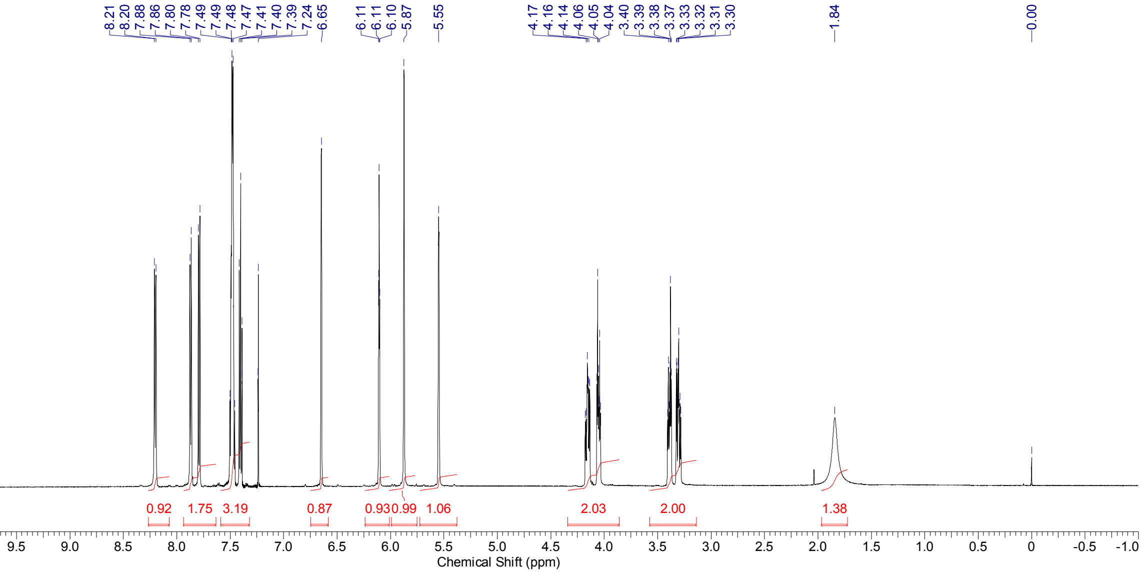

8.21
8.20
7.88
7.86
7.80
7.78
7.49
7.49
7.48
7.47
7.41
7.40
7.39
7.24
6.65
6.11
6.11
6.10
5.87
5.55
4.17
4.16
4.14
4.06
4.05
4.04
3.40
3.39
3.38
3.37
3.33
3.32
3.31
3.30
1.84
0.00
0.92
1.75
3.19
0.87
0.93
0.99
1.06
2.03
2.00
1.38
Chemical Shift (ppm)

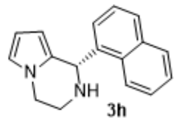

3h

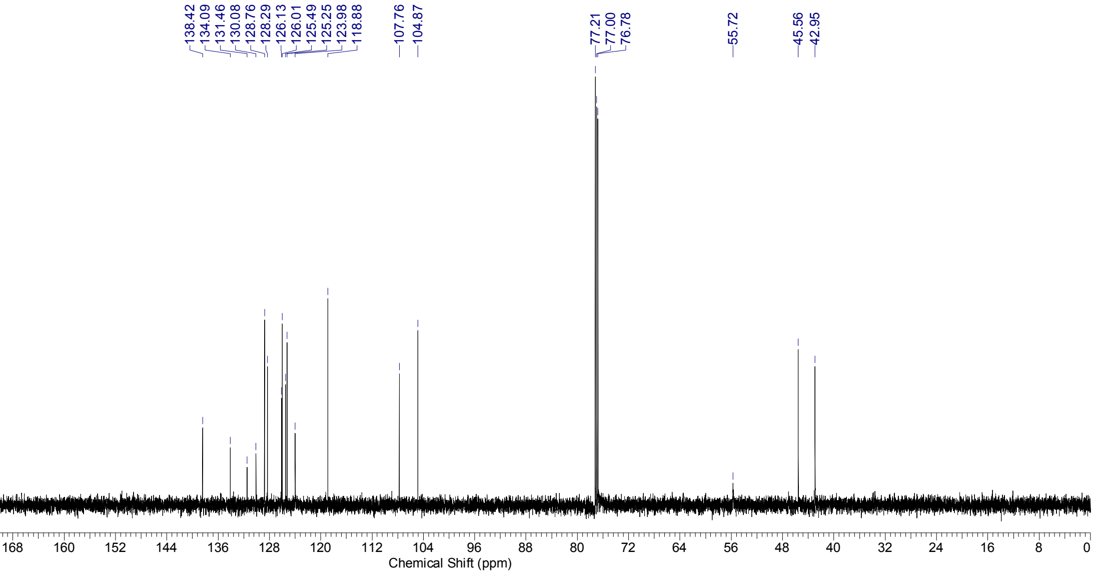

138.42
134.09
131.46
130.08
128.76
128.29
126.13
126.01
125.49
125.25
123.98
118.88
107.76
104.87
77.21
77.00
76.78
55.72
45.56
42.95
Chemical Shift (ppm)

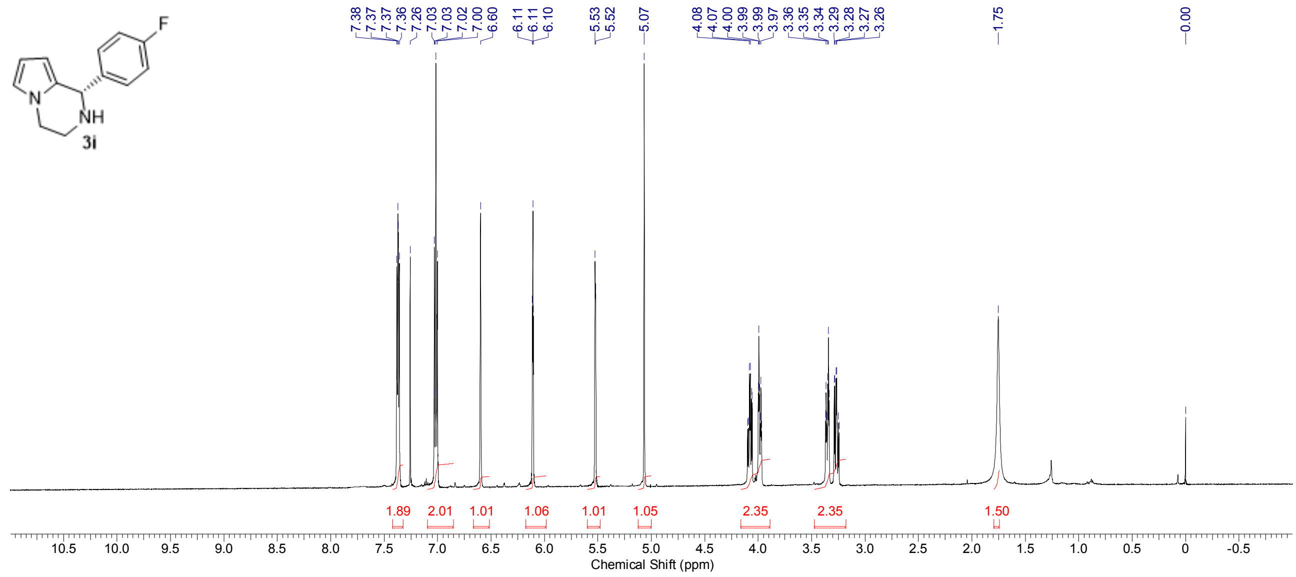
3i
7.38
7.37
7.37
7.36
7.26
7.03
7.03
7.02
7.00
6.60
6.11
6.11
6.10
5.53
5.52
5.07
4.08
4.07
4.00
3.99
3.99
3.97
3.36
3.35
3.34
3.29
3.28
3.27
3.26
1.75
0.00
1.89
2.01
1.01
1.06
1.01
1.05
2.35
2.35
1.50
Chemical Shift (ppm)

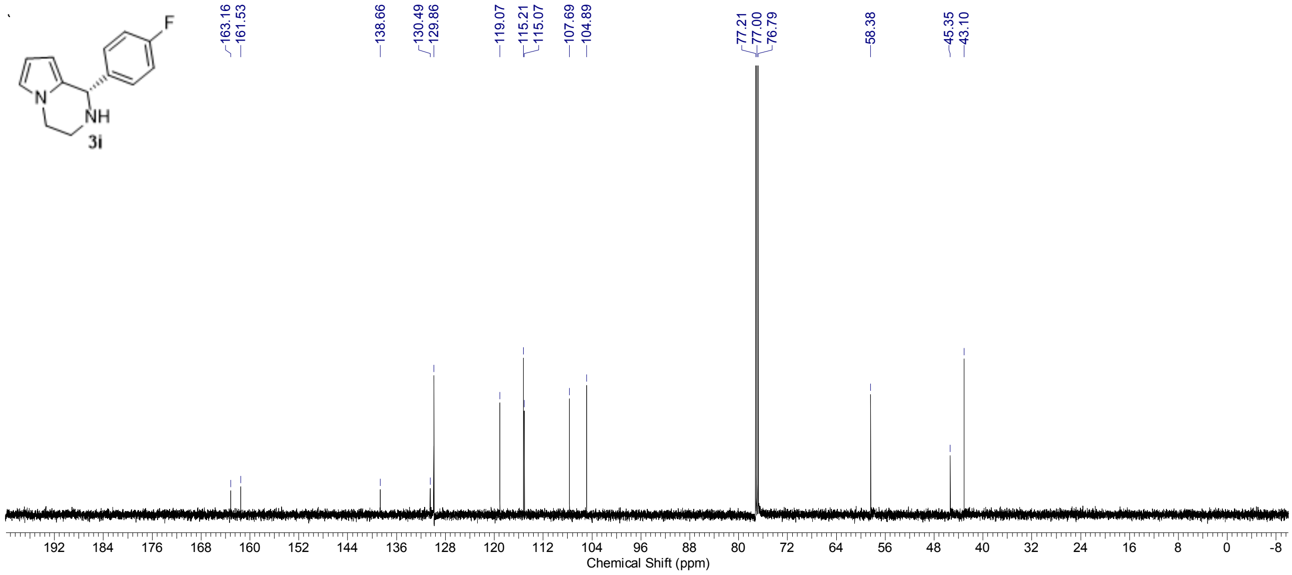
3i
163.16
161.53
138.66
130.49
129.86
119.07
115.21
115.07
107.69
104.89
77.21
77.00
76.79
58.38
45.35
43.10
Chemical Shift (ppm)

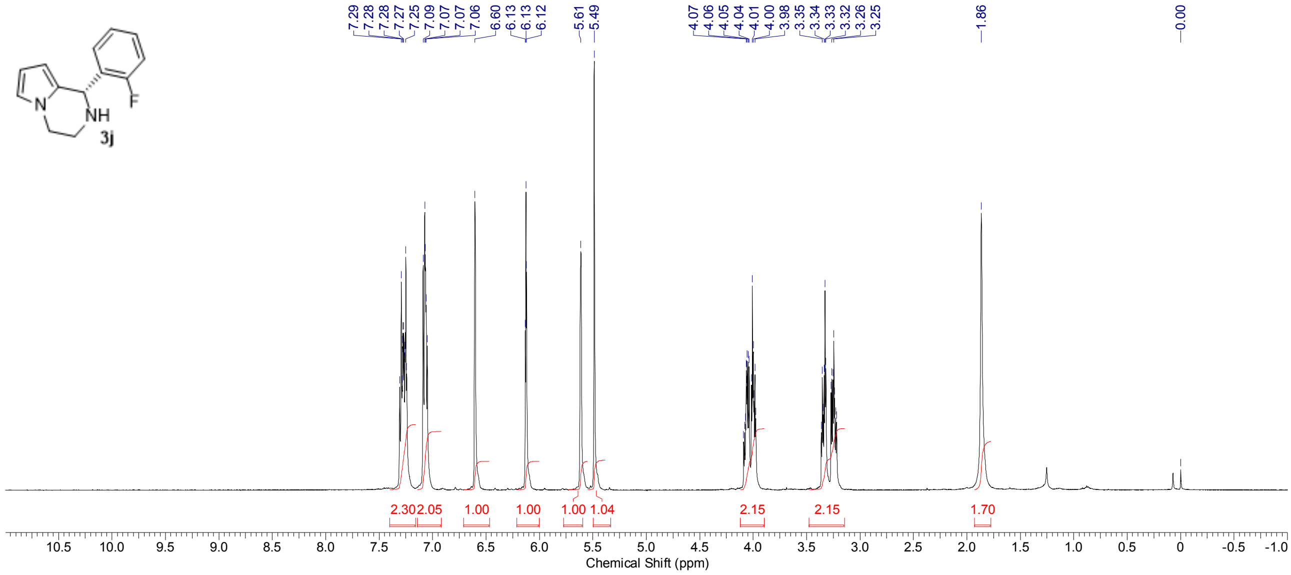
7.29
7.28
7.28
7.27
7.25
7.09
7.07
7.07
7.06
6.60
6.13
6.13
6.12
5.61
5.49
4.07
4.06
4.05
4.04
4.01
4.00
3.98
3.35
3.34
3.33
3.32
3.26
3.25
1.86
0.00
NH
F
N
3j
2.30
2.05
1.00
1.00
1.00
1.04
2.15
2.15
1.70
10.5 10.0 9.5 9.0 8.5 8.0 7.5 7.0 6.5 6.0 5.5 5.0 4.5 4.0 3.5 3.0 2.5 2.0 1.5 1.0 0.5 0 -0.5 -1.0
Chemical Shift (ppm)

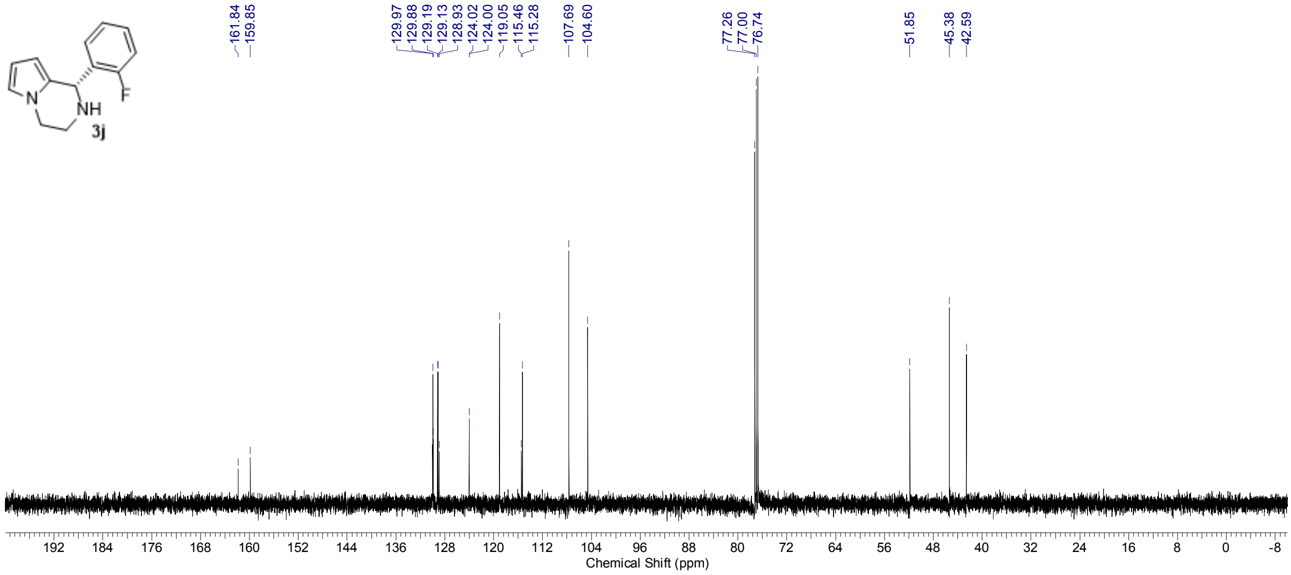
161.84
159.85
129.97
129.88
129.19
129.13
128.93
124.02
124.00
119.05
115.46
115.28
107.69
104.60
77.26
77.00
76.74
51.85
45.38
42.59
NH
F
N
3j
192 184 176 168 160 152 144 136 128 120 112 104 96 88 80 72 64 56 48 40 32 24 16 8 0 -8
Chemical Shift (ppm)

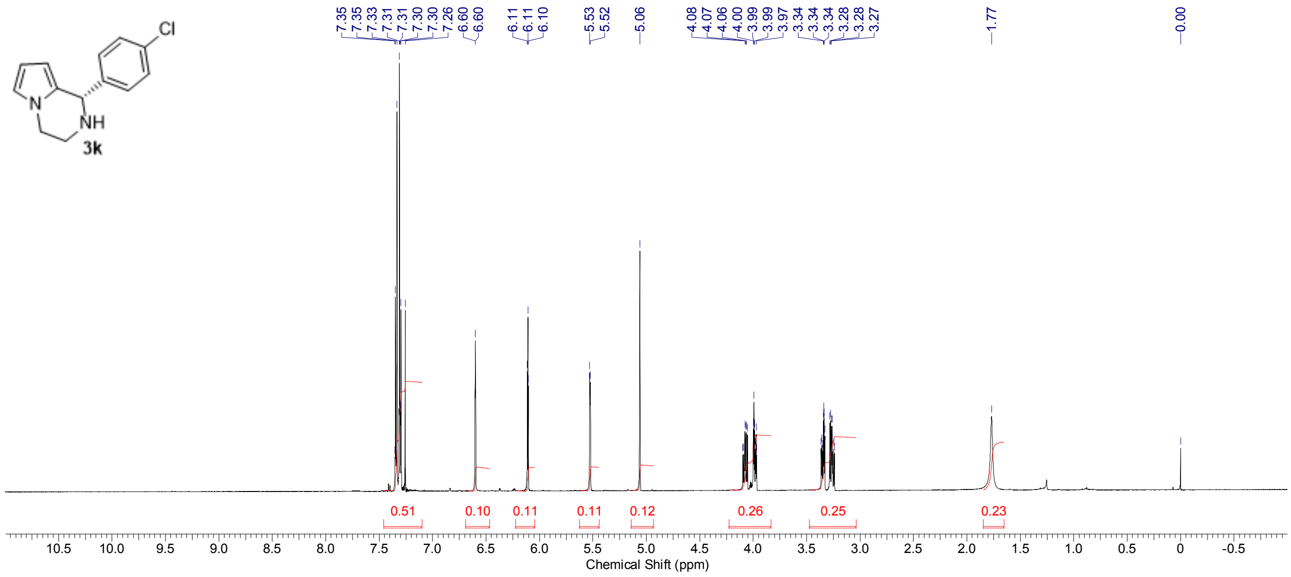
Cl
N
NH
3k
7.35
7.35
7.33
7.31
7.31
7.30
7.30
7.26
6.60
6.60
6.11
6.11
6.10
5.53
5.52
5.06
4.08
4.07
4.06
4.00
3.99
3.99
3.97
3.34
3.34
3.34
3.28
3.28
3.27
1.77
0.00
0.51
0.10
0.11
0.11
0.12
0.26
0.25
0.23
Chemical Shift (ppm)

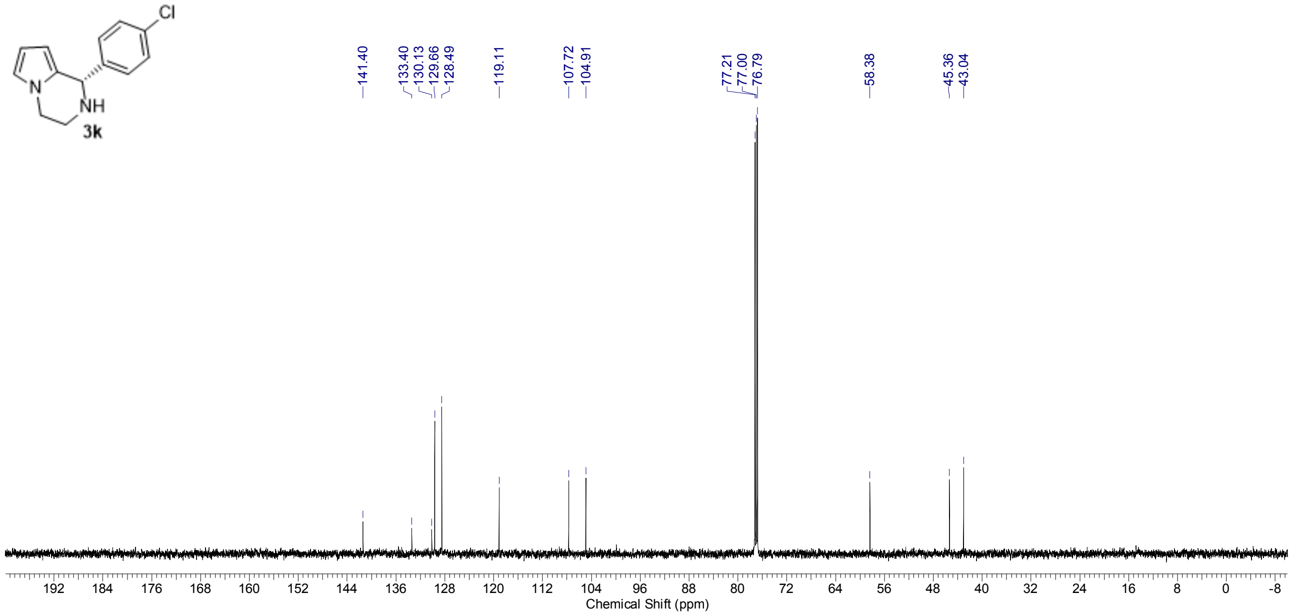
Cl
N
NH
3k
141.40
133.40
130.13
129.66
128.49
119.11
107.72
104.91
77.21
77.00
76.79
58.38
45.36
43.04
Chemical Shift (ppm)

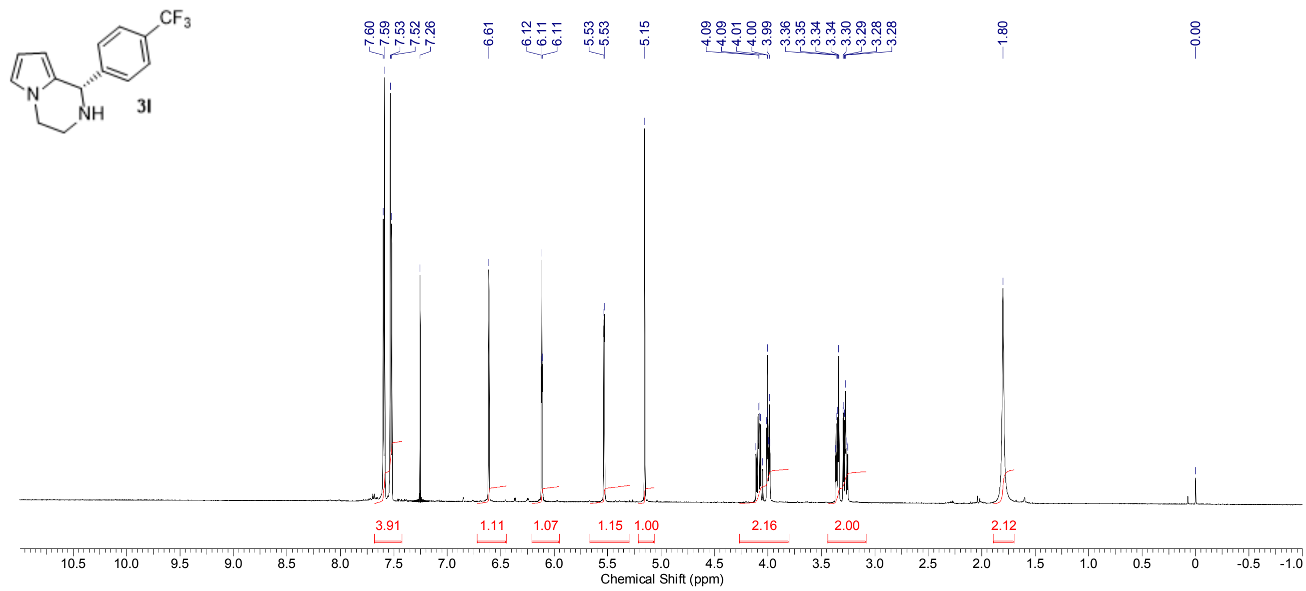
CF3
N
NH
3l
7.60
7.59
7.53
7.52
7.26
6.61
6.12
6.11
6.11
5.53
5.53
5.15
4.09
4.09
4.01
4.00
3.99
3.36
3.35
3.34
3.34
3.30
3.29
3.28
3.28
1.80
0.00
3.91
1.11
1.07
1.15
1.00
2.16
2.00
2.12
10.5 10.0 9.5 9.0 8.5 8.0 7.5 7.0 6.5 6.0 5.5 5.0 4.5 4.0 3.5 3.0 2.5 2.0 1.5 1.0 0.5 0 -0.5 -1.0
Chemical Shift (ppm)

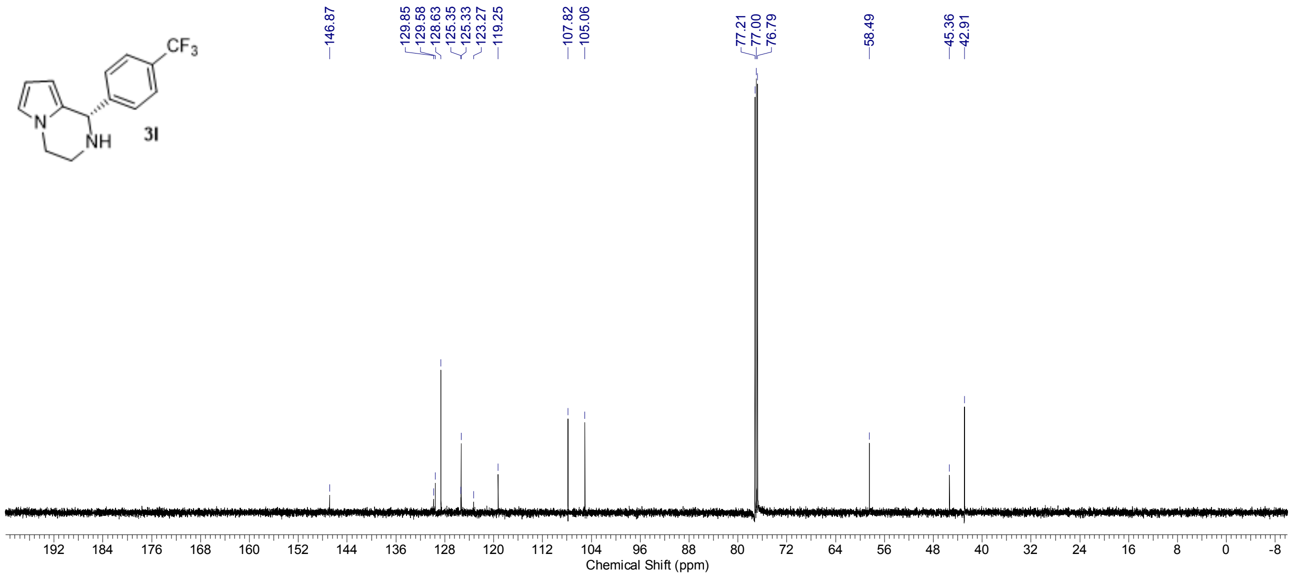
CF3
N
NH
3l
146.87
129.85
129.58
128.63
125.35
125.33
123.27
119.25
107.82
105.06
77.21
77.00
76.79
58.49
45.36
42.91
192 184 176 168 160 152 144 136 128 120 112 104 96 88 80 72 64 56 48 40 32 24 16 8 0 -8
Chemical Shift (ppm)

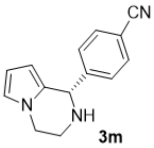

3m

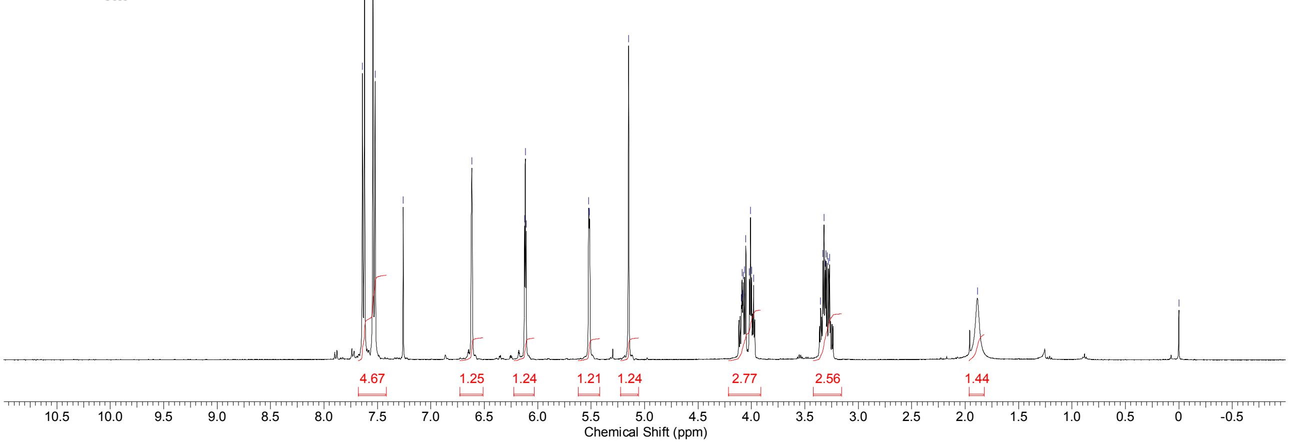
7.64
7.62
7.54
7.52
7.26
6.62
6.12
6.12
6.11
5.52
5.52
5.51
5.15
4.09
4.08
4.07
4.05
4.02
4.01
4.00
3.33
3.32
3.30
3.29
3.28
3.27
1.89
0.00
4.67
1.25
1.24
1.21
1.24
2.77
2.56
1.44
Chemical Shift (ppm)

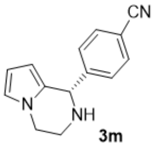

3m

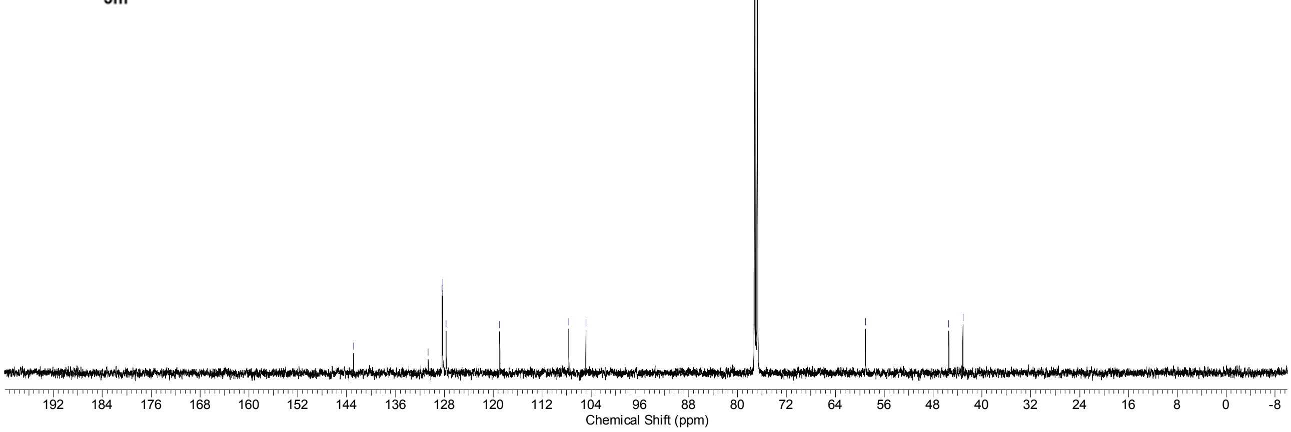
142.87
130.65
128.36
128.27
127.72
118.94
107.62
104.83
77.26
77.00
76.75
59.09
45.42
43.11
Chemical Shift (ppm)

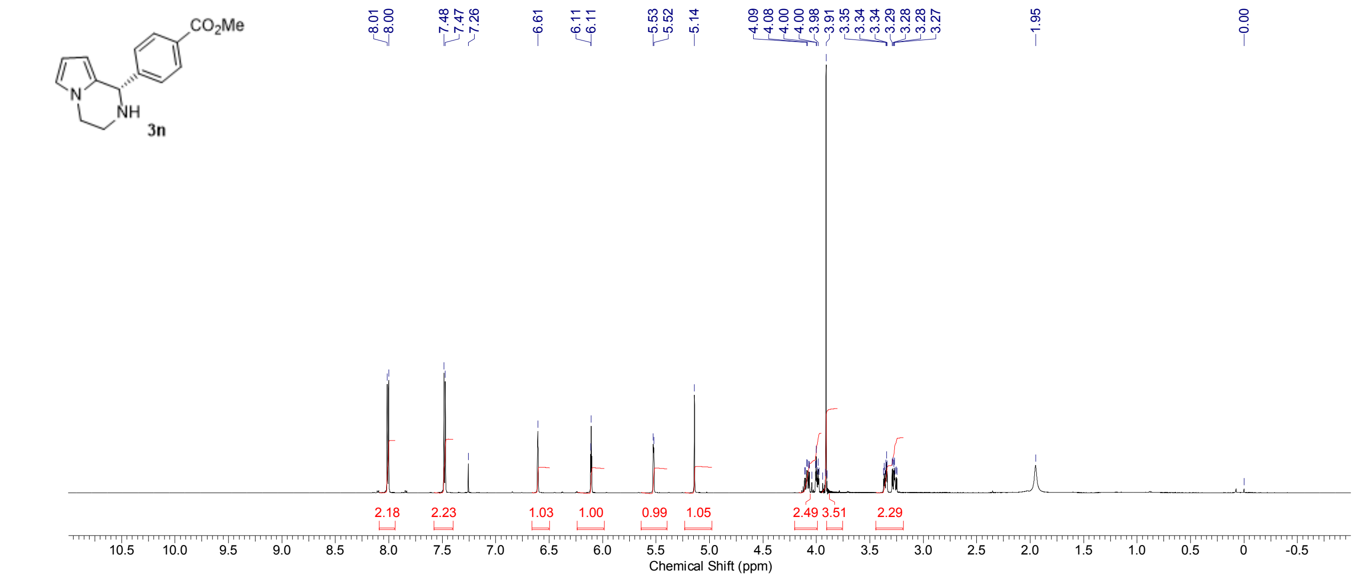
CO2Me
NH
3n
8.01
8.00
7.48
7.47
7.26
6.61
6.11
6.11
5.53
5.52
5.14
4.09
4.08
4.00
4.00
3.98
3.91
3.35
3.34
3.34
3.29
3.28
3.28
3.27
1.95
0.00
2.18
2.23
1.03
1.00
0.99
1.05
2.49
3.51
2.29
Chemical Shift (ppm)

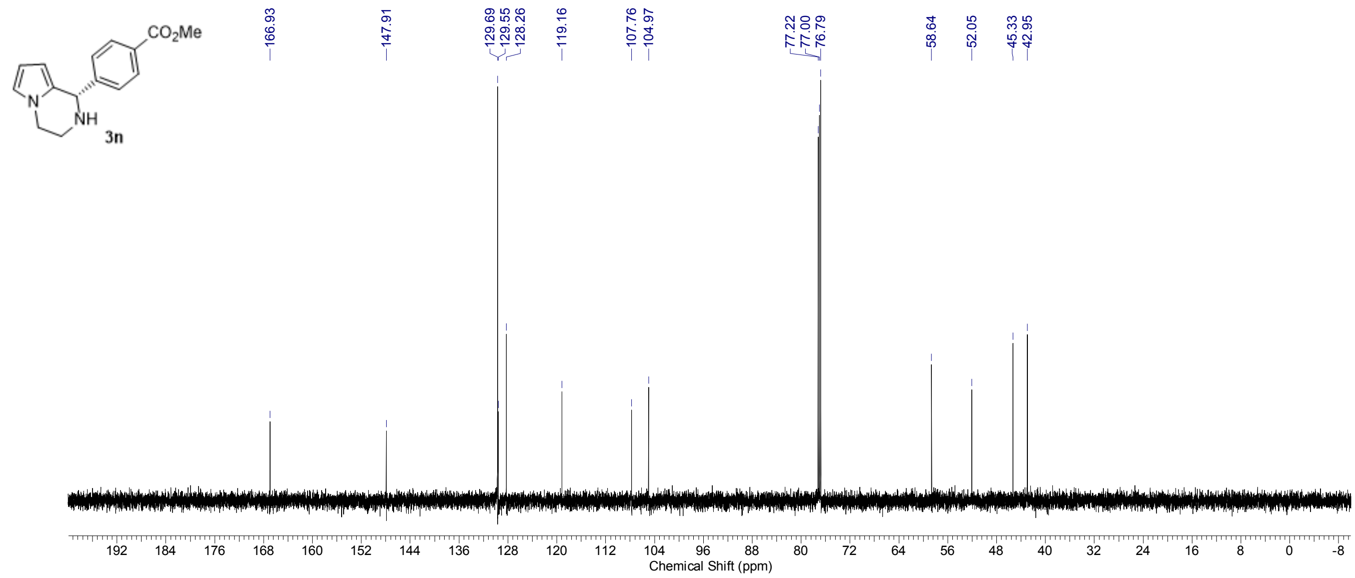
CO2Me
NH
3n
166.93
147.91
129.69
129.55
128.26
119.16
107.76
104.97
77.22
77.00
76.79
58.64
52.05
45.33
42.95
Chemical Shift (ppm)

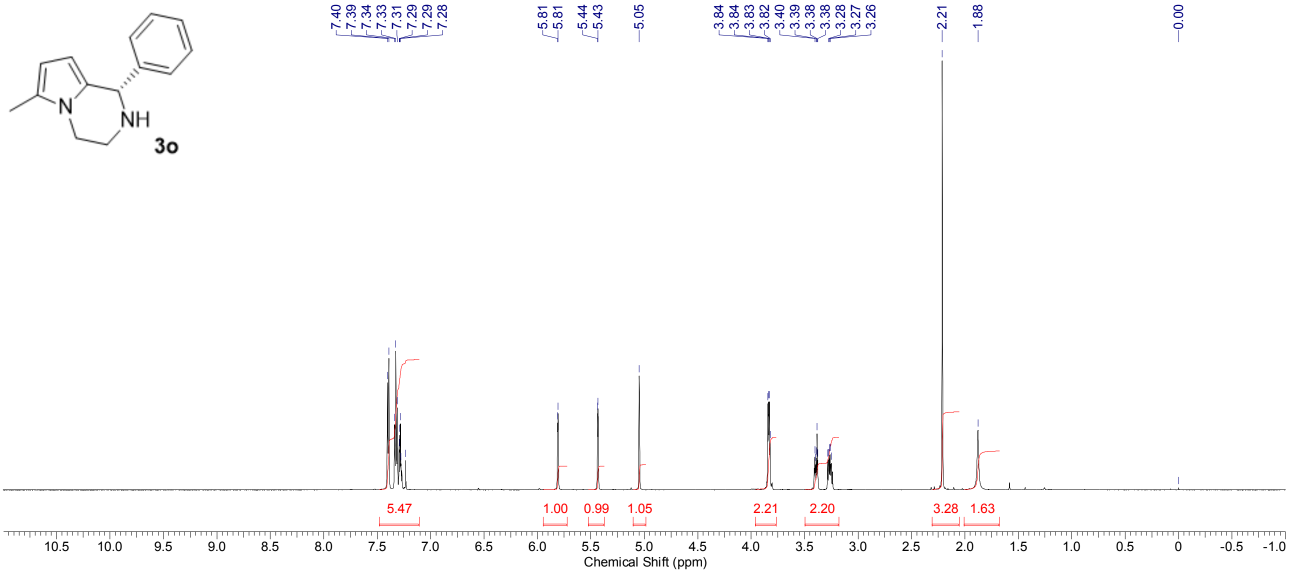
NH
3o
7.40 7.39 7.34 7.33 7.31 7.29 7.29 7.28
5.81 5.81 5.44 5.43
5.05
3.84 3.84 3.83 3.82 3.40 3.39 3.38 3.38 3.28 3.27 3.26
2.21
1.88
0.00
5.47 1.00 0.99 1.05 2.21 2.20 3.28 1.63
Chemical Shift (ppm)

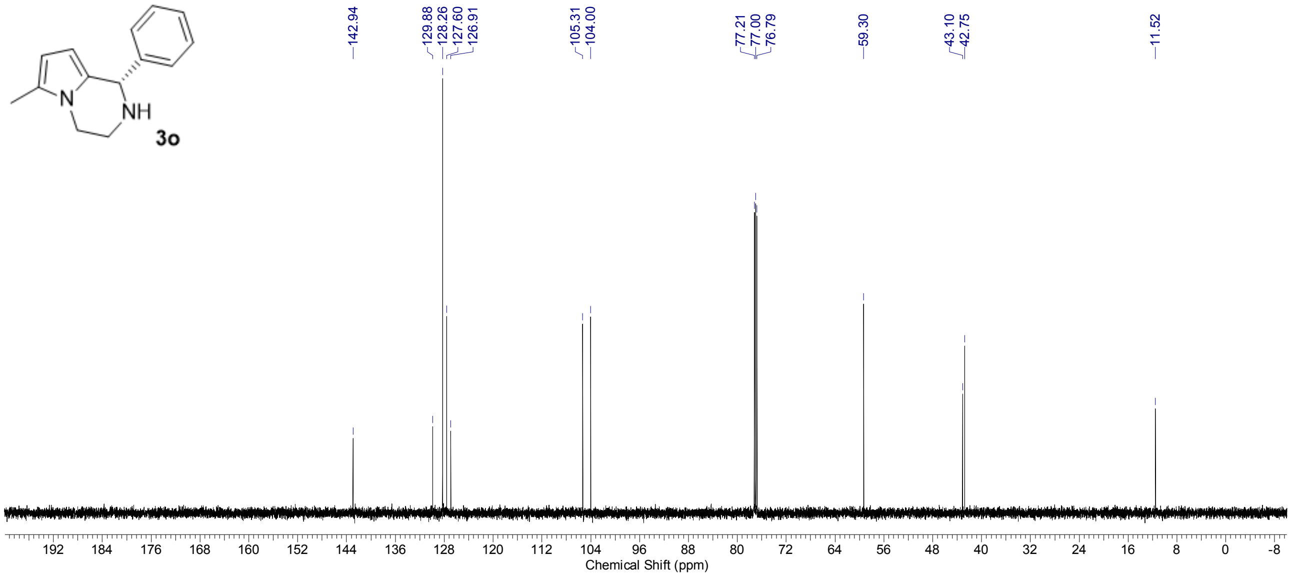
NH
3o
142.94
129.88 128.26 127.60 126.91
105.31 104.00
77.21 77.00 76.79
59.30
43.10 42.75
11.52
Chemical Shift (ppm)

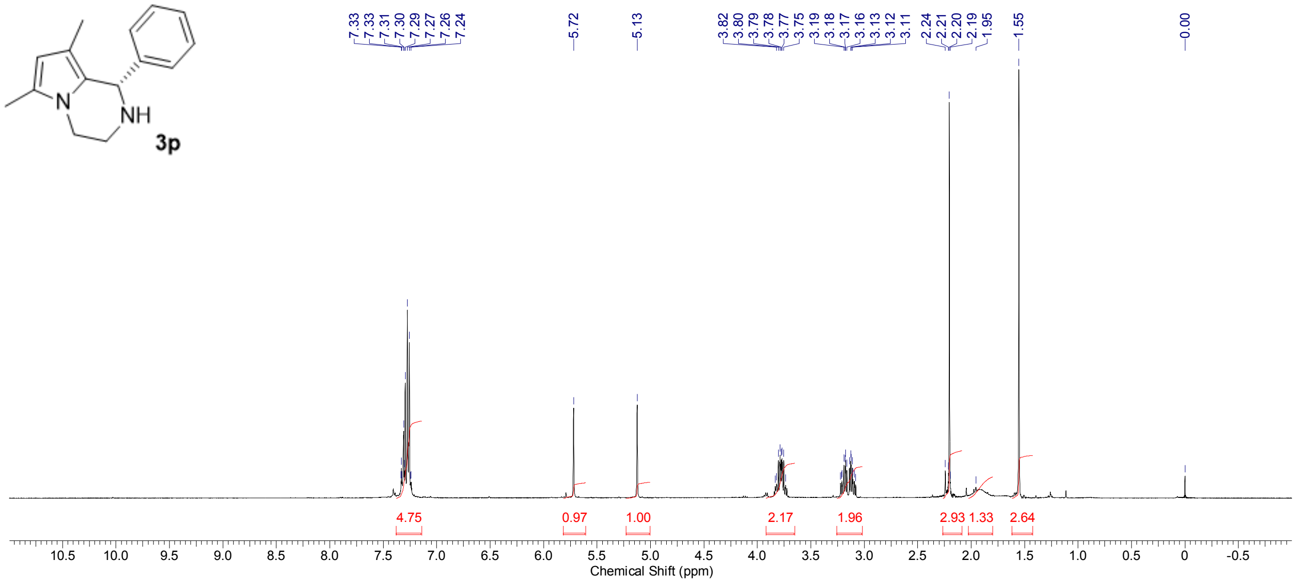
N
NH
3p
7.33 7.33 7.31 7.30 7.29 7.27 7.26 7.24
5.72
5.13
3.82 3.80 3.79 3.78 3.77 3.75 3.19 3.18 3.17 3.16 3.13 3.12 3.11
2.24 2.21 2.20 2.19 1.95
1.55
0.00
4.75
0.97
1.00
2.17
1.96
2.93 1.33 2.64
10.5 10.0 9.5 9.0 8.5 8.0 7.5 7.0 6.5 6.0 5.5 5.0 4.5 4.0 3.5 3.0 2.5 2.0 1.5 1.0 0.5 0 -0.5
Chemical Shift (ppm)

d

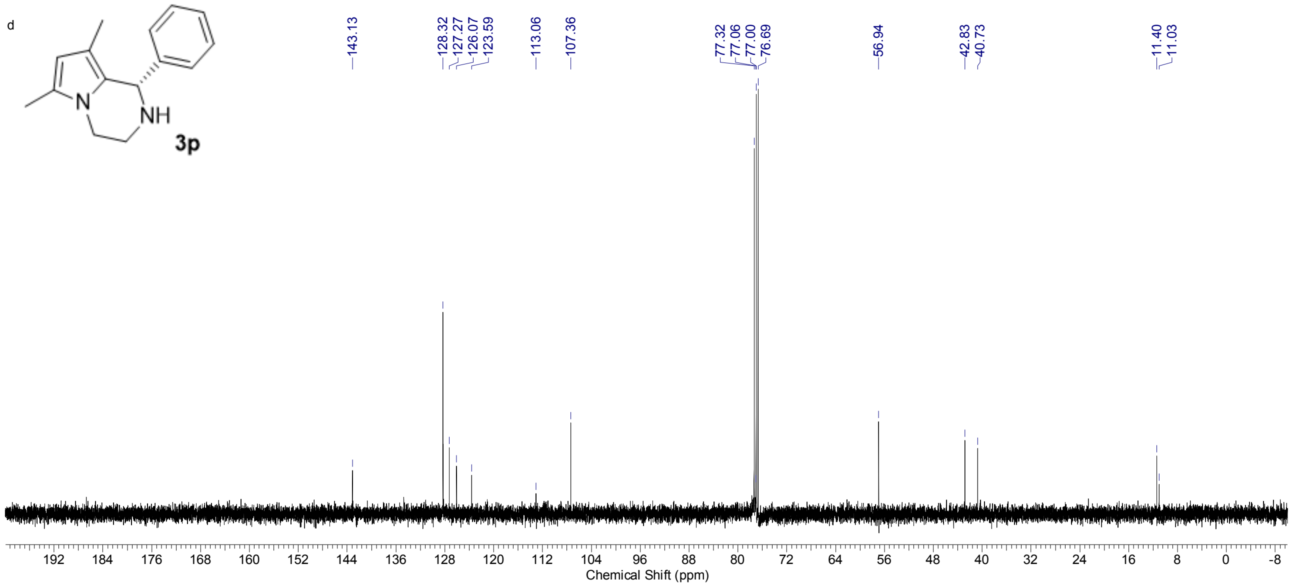
N
NH
3p
143.13
128.32 127.27 126.07 123.59
113.06
107.36
77.32 77.06 77.00 76.69
56.94
42.83 40.73
11.40 11.03
192 184 176 168 160 152 144 136 128 120 112 104 96 88 80 72 64 56 48 40 32 24 16 8 0 -8
Chemical Shift (ppm)

6. Copies of the HPLC Chromatograms

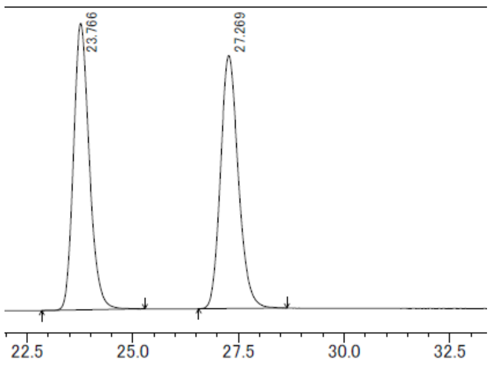

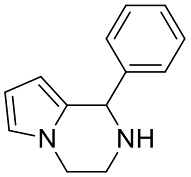

rac-3a

Peak#	Ret. Time	Area	Height	Area%
1	23.766	1400524	53907	50.052
2	27.269	1397601	47639	49.948

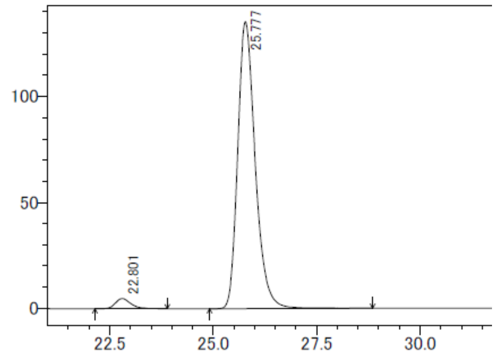

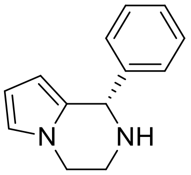

3a

Peak#	Ret. Time	Area	Height	Area%
1	22.801	123682	4808	3.012
2	25.777	3982402	135351	96.988

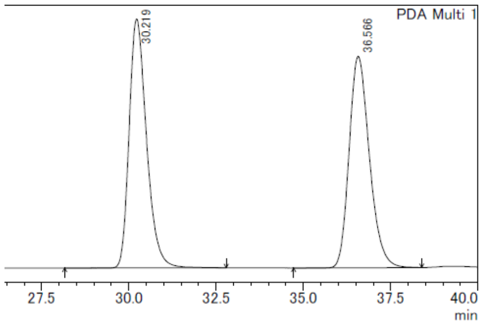

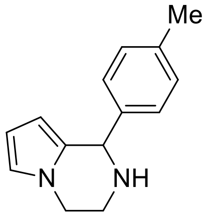

rac-3b

PDA Ch1 250nm 4nm

Peak#	Ret. Time	Area	Height	Area%
1	30.219	7797601	224692	50.266
2	36.566	7715141	190908	49.734

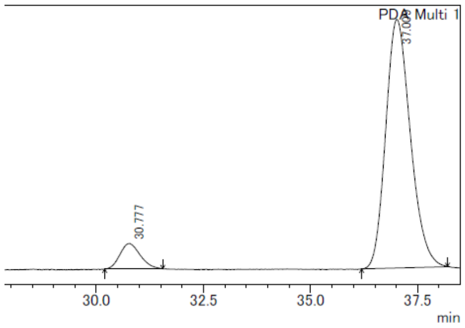

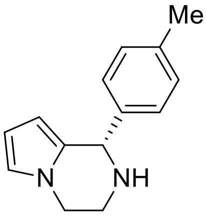

3b

PDA Ch1 250nm 4nm

Peak#	Ret. Time	Area	Height	Area%
1	30.777	61165	1863	7.772
2	37.009	725876	18131	92.228

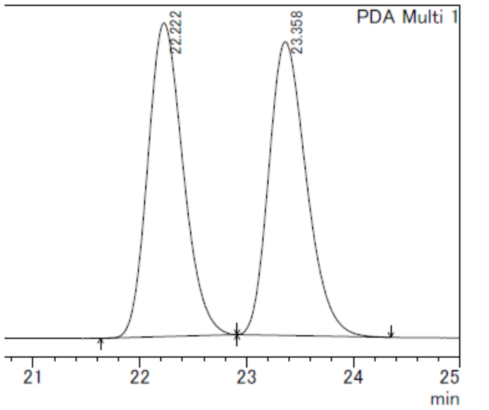

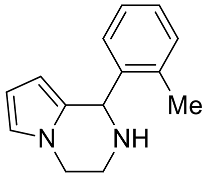

rac-3c

PDA Ch1 250nm 4nm

Peak#	Ret. Time	Area	Height	Area%
1	22.222	2203777	95666	50.120
2	23.358	2193202	89623	49.880

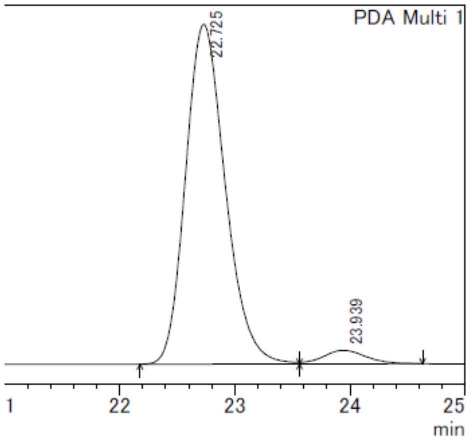

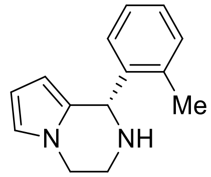

3c

PDA Ch1 250nm 4nm

Peak#	Ret. Time	Area	Height	Area%
1	22.725	2392838	99912	95.831
2	23.939	104100	4014	4.169

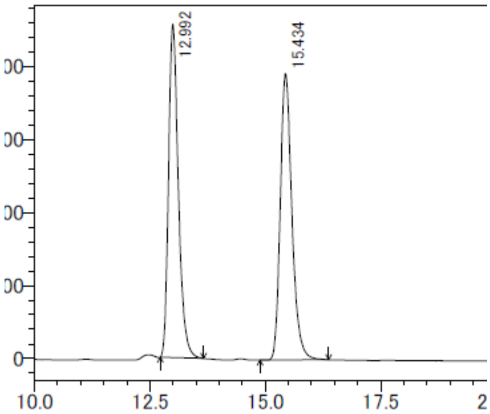

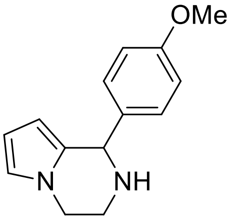

rac-3d

PDA Ch1 250nm 4nm

Peak#	Ret. Time	Area	Height	Area%
1	12.992	6768719	457016	49.527
2	15.434	6897992	392623	50.473

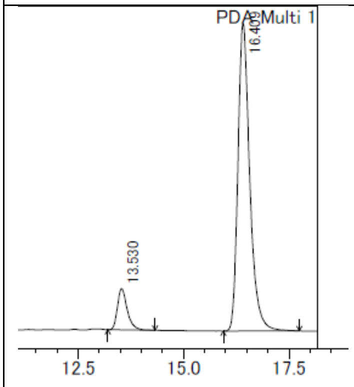

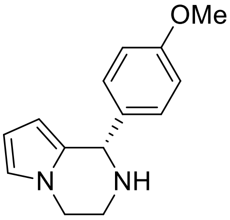

3d

PDA Ch1 250nm 4nm

Peak#	Ret. Time	Area	Height	Area%
1	13.530	254030	15789	10.001
2	16.409	2285998	117299	89.999

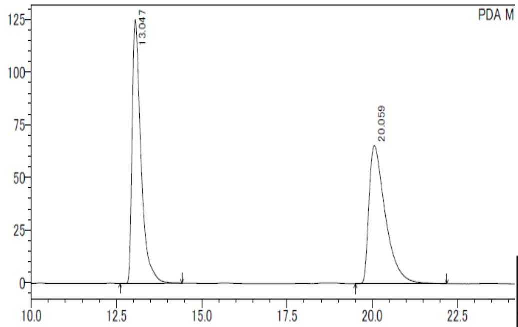

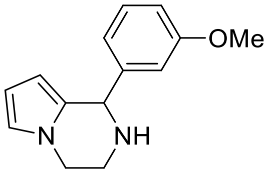

rac-3e

PDA Ch1 250nm 4nm

Peak#	Ret. Time	Area	Height	Area%
1	13.047	2259152	125180	50.653
2	20.059	2200887	65514	49.347

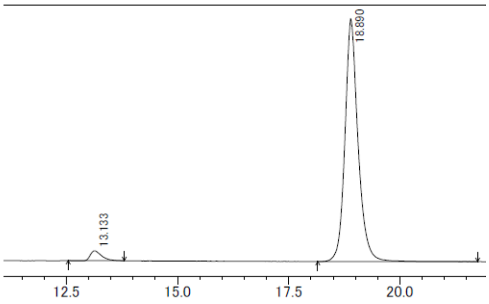

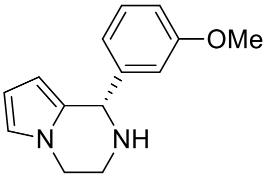

3e

PDA Ch1 250nm 4nm

Peak#	Ret. Time	Area	Height	Area%
1	13.133	1056854	61097	3.284
2	18.890	31123554	1520042	96.716

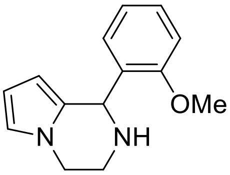

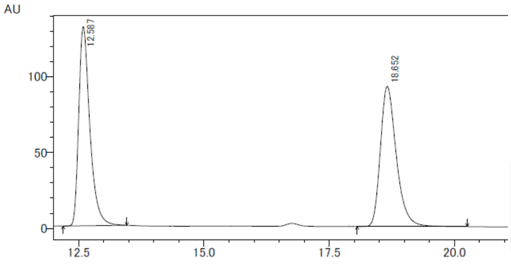

rac-3f

PDA Ch1 250nm 4nm

Peak#	Ret. Time	Area	Height	Area%
1	12.587	2027441	131213	49.838
2	18.652	2040626	92458	50.162

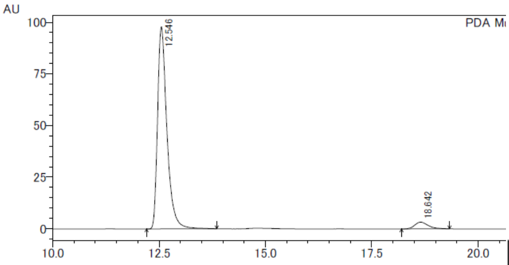

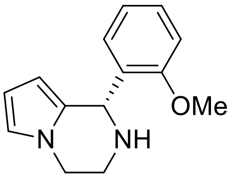

3f

PDA Ch1 250nm 4nm

Peak#	Ret. Time	Area	Height	Area%
1	12.546	1498386	97885	95.415
2	18.642	72010	3258	4.585

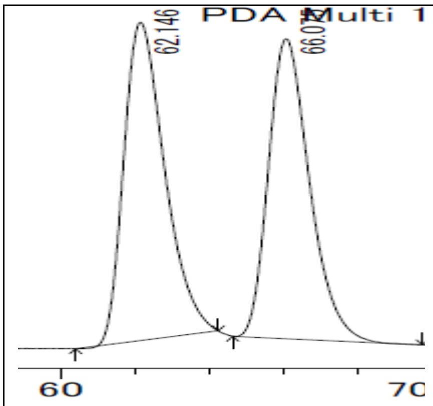

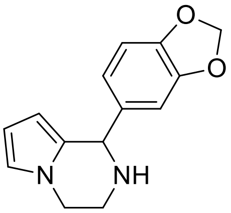

rac-3g

PDA Ch1 250nm 4nm

Peak#	Ret. Time	Area	Height	Area%
1	62.146	2220500	28962	51.293
2	66.075	2108565	27302	48.707

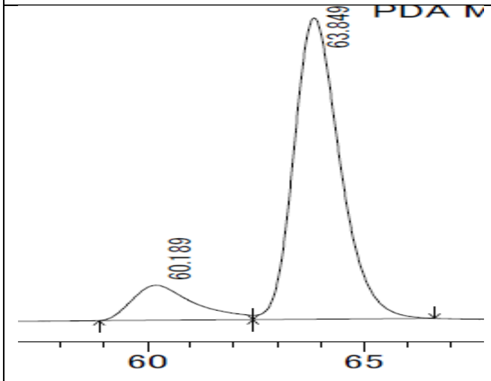

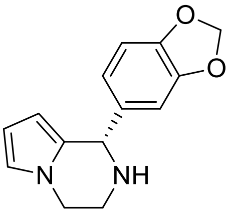

3g

PDA Ch1 250nm 4nm

Peak#	Ret. Time	Area	Height	Area%
1	60.189	671917	6755	13.435
2	63.849	4329320	58218	86.565

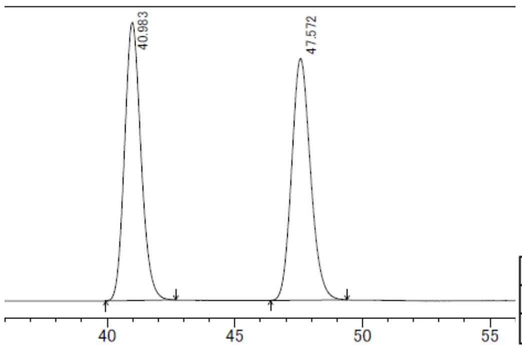

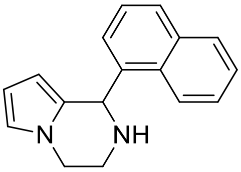

rac-3h

PDA Ch1 250nm 4nm

Peak#	Ret. Time	Area	Height	Area%
1	40.983	4271449	95349	50.015
2	47.572	4268915	82842	49.985

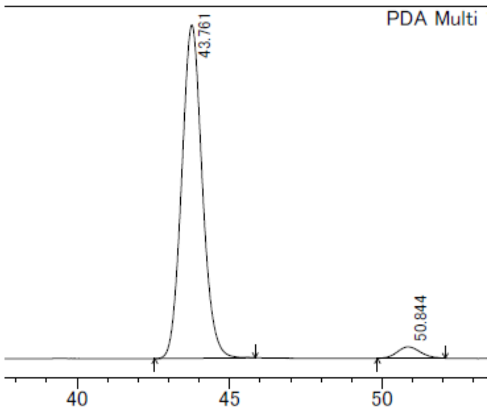

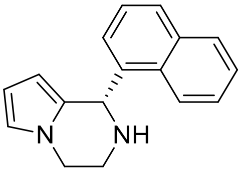

3h

PDA Ch1 250nm 4nm

Peak#	Ret. Time	Area	Height	Area%
1	43.761	9425265	200967	96.298
2	50.844	362347	6906	3.702

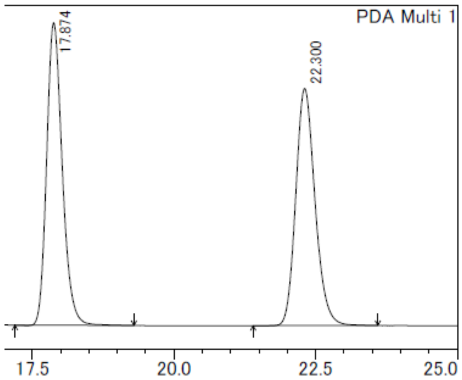

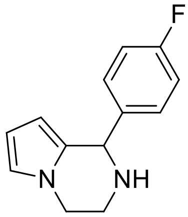

rac-3i

PDA Ch1 250nm 4nm

Peak#	Ret. Time	Area	Height	Area%
1	17.874	5324813	277913	50.846
2	22.300	5147692	217868	49.154

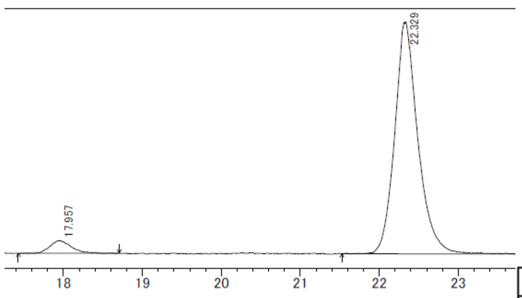

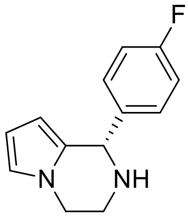

3i

PDA Ch1 250nm 4nm

Peak#	Ret. Time	Area	Height	Area%
1	17.957	902502	49137	4.691
2	22.329	18334801	895993	95.309

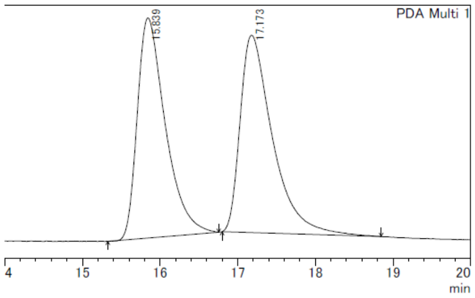

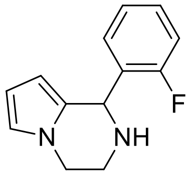

rac-3j

PDA Ch1 250nm 4nm

Peak#	Ret. Time	Area	Height	Area%
1	15.839	627845	24985	49.184
2	17.173	648667	22392	50.816

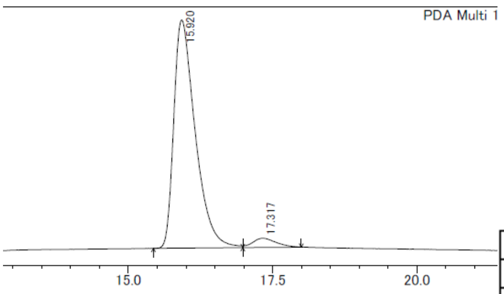

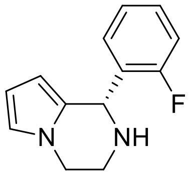

3j

PDA Ch1 250nm 4nm

Peak#	Ret. Time	Area	Height	Area%
1	15.920	1286018	49838	95.742
2	17.317	57190	2030	4.258

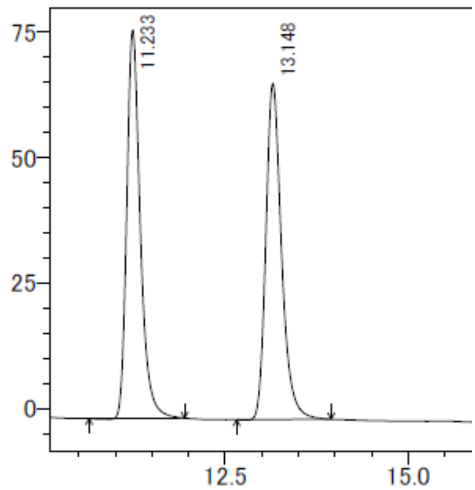

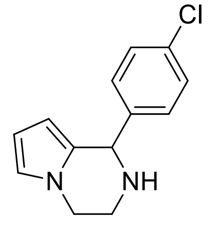

rac-3k

PDA Ch1 250nm 4nm

Peak#	Ret. Time	Area	Height	Area%
1	11.233	989918	77307	49.858
2	13.148	995559	66992	50.142

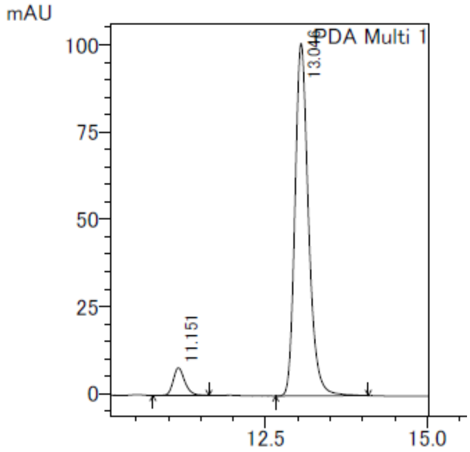

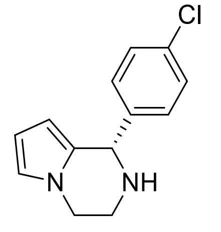

3k

PDA Ch1 250nm 4nm

Peak#	Ret. Time	Area	Height	Area%
1	11.151	98108	7944	6.262
2	13.046	1468611	100956	93.738

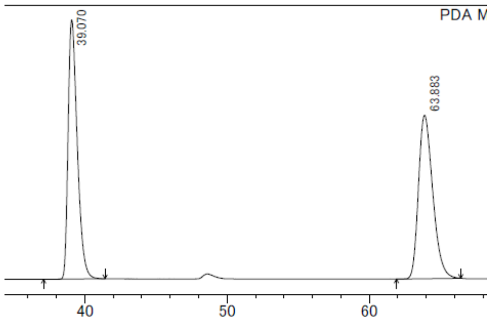

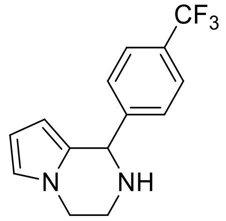

rac-3l

PDA Ch1 250nm 4nm

Peak#	Ret. Time	Area	Height	Area%
1	39.070	9843235	219246	50.551
2	63.883	9628802	138260	49.449

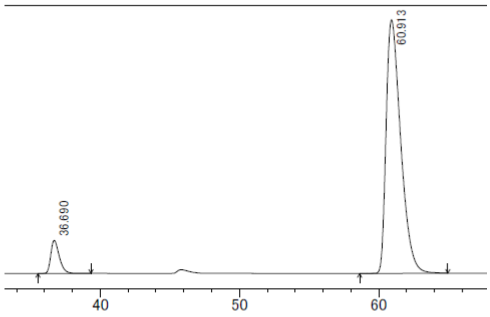

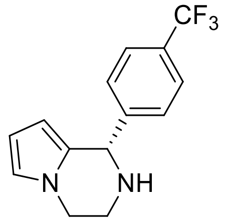

3l

PDA Ch1 250nm 4nm

Peak#	Ret. Time	Area	Height	Area%
1	36.690	4363259	102596	7.113
2	60.913	56981355	784132	92.887

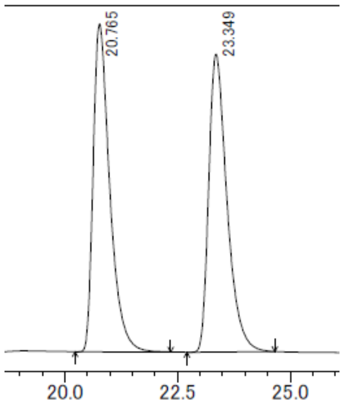

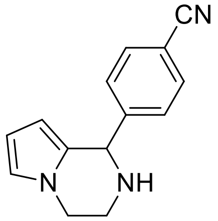

rac-3m

PDA Ch1 250nm 4nm

Peak#	Ret. Time	Area	Height	Area%
1	20.765	8621724	336274	50.044
2	23.349	8606604	305166	49.956

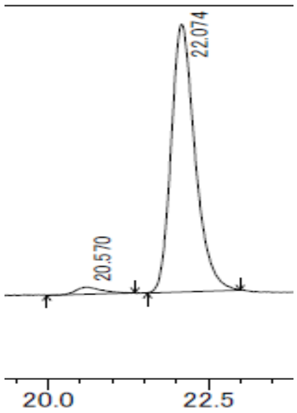

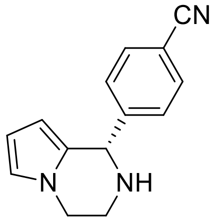

3m

PDA Ch1 250nm 4nm

Peak#	Ret. Time	Area	Height	Area%
1	20.570	7914	290	2.698
2	22.074	285406	11012	97.302

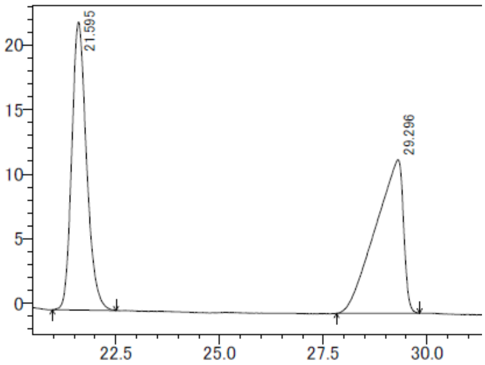

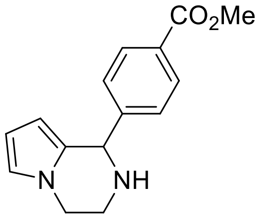

rac-3n

PDA Ch1 250nm 4nm

Peak#	Ret. Time	Area	Height	Area%
1	21.595	553750	22308	49.766
2	29.296	558953	11892	50.234

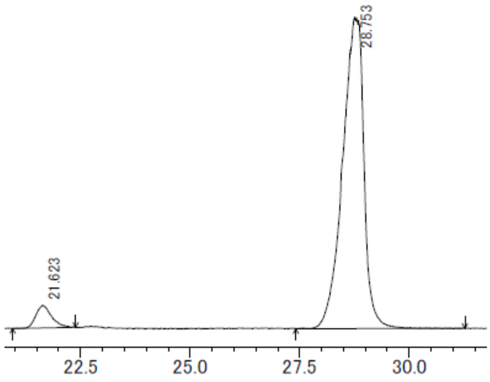

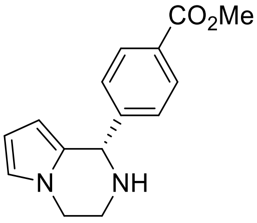

3n

PDA Ch1 250nm 4nm

Peak#	Ret. Time	Area	Height	Area%
1	21.623	3039523	121516	5.104
2	28.753	56508110	1672996	94.896

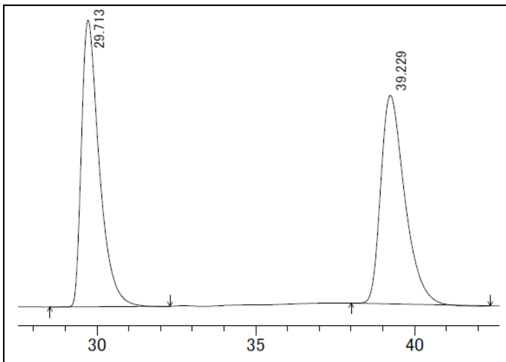

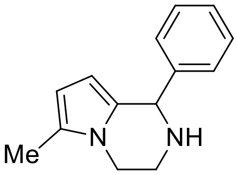

rac-3o

PDA Ch1 220nm 4nm

Peak#	Ret. Time	Area	Height	Area%
1	29.713	8252910	212235	50.395
2	39.229	8123663	154486	49.605

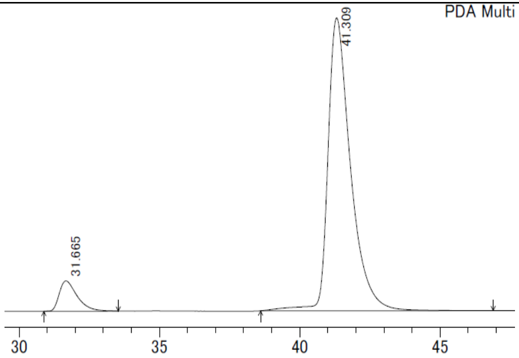

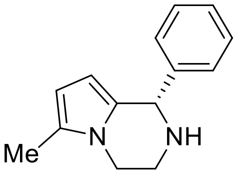

3o

PDA Ch1 220nm 4nm

Peak#	Ret. Time	Area	Height	Area%
1	31.665	1455225	32852	7.537
2	41.309	17851270	317908	92.463

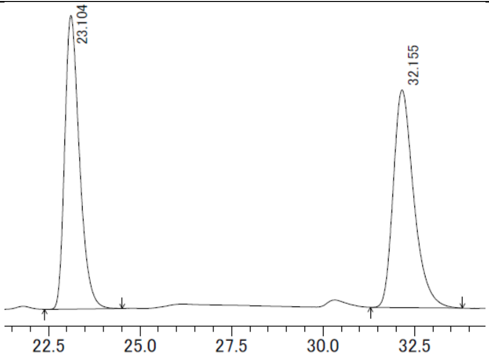

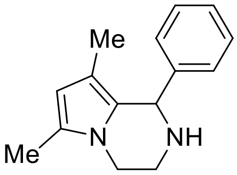

rac-3p

PDA Ch1 220nm 4nm

Peak#	Ret. Time	Area	Height	Area%
1	23.104	3976361	141563	49.194
2	32.155	4106601	104958	50.806

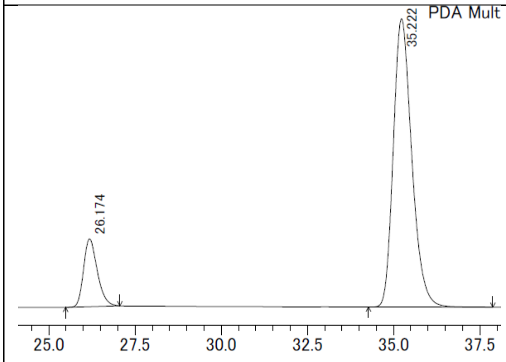

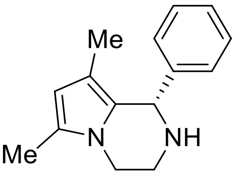

3p

PDA Ch1 220nm 4nm

Peak#	Ret. Time	Area	Height	Area%
1	26.174	4823120	174512	14.847
2	35.222	27662318	742478	85.153

7. References

1. T. Akiyama, Y. Honma, J. Itoh, K. Fuchibe, *Adv. Synth. Catal.* 2008, **350**, 399.
2. H. Mizuno, J. Takaya, N. Iwasawa, *J. Am. Chem. Soc.* 2011, **133**, 1251.
3. W.-J. Yoo, H. Miyamura, S. Kobayashi, *J. Am. Chem. Soc.* 2011, **133**, 3095.
4. Y. He, M. Lin, Z. Li, X. Liang, G. Li, J. C. Antilla, *Org. Lett.* 2011, **13**, 4490.
